# C-C motif receptor 2 is a core profibrotic factor in uremic cardiomyopathy

**DOI:** 10.1242/dmm.052395

**Published:** 2026-02-03

**Authors:** Jing-Fu Bao, Bao-Cheng Guo, Jia-Ju Mo, Hongguo Zhu, Nan Jia, Fanfan Hou, Youhua Liu, Aiqing Li

**Affiliations:** ^1^ State Key Laboratory of Organ Failure Research, National Clinical Research Center for Kidney Disease, Nanfang Hospital, Southern Medical University, 510515 Guangzhou, China; ^2^ Guangdong Provincial Institute of Nephrology, Guangdong Provincial Key Laboratory of Renal Failure Research, Guangdong Provincial Clinical Research Center for Kidney Disease, 510005 Guangzhou, China; ^3^ Department of Nephrology, The Fourth Affiliated Hospital of Guangzhou Medical University, 511300 Guangzhou, China

**Keywords:** Rodent model, Uremic cardiomyopathy, Transcriptome, Fibrosis, C-C chemokine receptor 2

## Abstract

Uremic cardiomyopathy (UC) represents a leading cause of mortality in patients with chronic kidney disease (CKD), characterized by left ventricular hypertrophy (LVH) and fibrosis. The underlying mechanisms of UC pathogenesis remain incompletely understood. This study developed two methods to simulate human UC – modified nephrectomy (MNx) and adenine-normal combinational diet – and compared these approaches across several rodent strains. Transcriptomic analysis was performed on left ventricular tissues from these models. The analysis revealed global changes in UC, including dysregulated cell cycle processes, enhanced extracellular matrix remodeling and metabolic abnormalities, while also highlighting molecular distinctions between MNx- and adenine-induced UC. Notably, this study identified C-C-motif receptor 2 (CCR-2) as a novel potential antifibrotic target in UC. CCR-2 blockade substantially reversed fibrosis without affecting LVH. The mechanism through which CCR-2 inhibition suppresses cardiac fibrosis development in UC appears to involve the promotion of cardiac residual macrophage expansion. These findings establish a central role for CCR-2 in cardiac fibrosis and suggest CCR-2 inhibition as a promising therapeutic target for UC.

## Teaser

**Summary:** This study provides a systematic comparison of various rodent models of uremic cardiomyopathy and the first comprehensive transcriptomic landscape of uremic cardiomyopathy.

## INTRODUCTION

Chronic kidney disease (CKD) represents a significant global public health challenge (GBD Chronic Kidney Disease Collaboration, 2020), with cardiovascular complications emerging as the primary cause of mortality in patients with CKD (Wang and Shapiro, 2019). Uremic cardiomyopathy (UC) constitutes the most prevalent characteristic of cardiovascular alterations in CKD (Wang and Shapiro, 2019), manifesting primarily as left ventricular hypertrophy (LVH) and fibrosis. UC leads to more severe cardiovascular complications, including diastolic dysfunction, heart failure, arrhythmia and sudden death (Nakamura and Sadoshima, 2018), establishing it as a critical therapeutic target in patients with CKD. Multiple factors contribute to UC development, including hemodynamic overload, renin-angiotensin system overactivation, autonomic nerve system dysfunction, microinflammation, oxidative stress, uremic toxin accumulation, renal anemia and phosphorus metabolism abnormalities (Wang and Shapiro, 2019). However, therapeutic strategies for UC remain elusive, primarily due to insufficient understanding of its molecular mechanisms. Elucidating the global molecular changes in cardiac response to CKD may reveal novel therapeutic approaches to improve patient outcomes.

A model of UC is a prerequisite for investigating the pathogenesis of UC. Previous studies showed that 5/6 nephrectomy (5/6 Nx) is unable to induce a stable UC model and significant hyperphosphatemia (Bufi and Korstanje, 2022; Hamzaoui et al., 2020; Hofman-Bang et al., 2010; Moench et al., 2021; Soppert et al., 2022; Wollenhaupt et al., 2022; Yamazaki-Nakazawa et al., 2014), a condition frequently observed in patients with CKD and strongly associated with cardiovascular risk (Vervloet and van Ballegooijen, 2018). Adenine intake represents another standard method for establishing CKD models, requiring no surgical intervention and resulting in lower mortality rates (Chen et al., 2021; Diwan et al., 2018; Kieswich et al., 2018; Yokozawa et al., 1986). Although single-adenine diet induces LVH and hyperphosphatemia in Sprague Dawley (SD) rats (Kashioulis et al., 2018), this effect is not observed in mice (Chen et al., 2021; Kieswich et al., 2018). Notably, the C57BL/6J strain, which is amenable to genetic manipulation, shows relative resistance to CKD and UC (Hamzaoui et al., 2020; Leelahavanichkul et al., 2010; Liang and Liu, 2023), despite numerous studies establishing UC models in this strain. For instance, Wollenhaupt et al. (2022) compared cardiac changes in 5/6 Nx- and adenine-induced models using C57BL/6J or C57BL/6N mice, yet neither significant cardiac remodeling nor cardiac dysfunction was detected. Collectively, 5/6 Nx and adenine diet can be used to cause CKD, but the cardiac responses to these approaches vary considerably (Soppert et al., 2022). Thus, developing a stable rodent UC model that closely mimics human UC remains necessary.

This study compared cardiac changes across several rodent CKD models induced by 5/6 Nx and two novel approaches – namely, modified nephrectomy (MNx) and adenine-normal combinational diet (Fig. S1) – and selected two models demonstrating significant UC phenotypes for transcriptomic analysis. The analysis examined molecular changes in UC in various processes, particularly cell cycle processes, energetic metabolism and inflammation/extracellular matrix remodeling, which are all associated with UC phenotypes. Additionally, the study compared molecular changes between the two UC models and identified hub genes in networks of the aforementioned pathophysiological processes. Finally, the functions of hub genes in the inflammation/extracellular matrix remodeling network were verified, identifying a potential anti-inflammatory antifibrotic target for UC.

## RESULTS

### MNx shortens time consumption for UC model establishment (model 1 and 2)

#### Model 1

Male SD rats are frequently employed in establishing the nephrectomy-induced model of CKD (Adam et al., 2022). Male SD rats with 5/6 Nx exhibited significant increases in relative wall thickness (RWT) and left ventricular (LV) weight after 12 weeks post-surgery, accompanied by unchanged left ventricular ejection fraction (LVEF), increased LV weight/heart weight ratio and myocardial fibrosis (Fig. S2A-C). The nephrectomized rats exhibited reduced body weight, progressive elevation in blood pressure, increased serum creatinine and urea nitrogen, tubular dilation, glomerular hypertrophy and interstitial fibrosis (Fig. S2D-F). However, hyperphosphatemia, a common condition in patients with CKD strongly linked to cardiovascular risk (Vervloet and van Ballegooijen, 2018), was not observed (Fig. S2D). These findings indicate that 5/6 Nx requires a minimum of 12 weeks to establish the UC model and lacks certain important cardiovascular-related features of CKD.

#### Model 2

To reduce the duration, we expanded the resection area (Fig. S3A), which may accelerate UC progression. Compared to male SD rats with 5/6 Nx, male SD rats with MNx showed progressive increases in LV thickness, LV weight and heart weight, with significant LVH observed by the fifth week post-MNx, as evidenced by increased LV thickness, LV weight, and expression of *Nppa* and *Nppb* (Fig. S3B,C, Fig. S4A-C, Table S3). Interestingly, an increased RWT without significant heart weight elevation could be found in the first and third weeks after surgery (Fig. S3C), although this could be attributable to increased LV wall thickness and decreased LV diameter, which aim to maintain LV wall tension in response to elevated blood pressure (Fig. S3D). Additionally, rats with MNx demonstrated increased cardiomyocyte cross-sectional area, LV interstitial fibrosis and cardiomyocyte apoptosis by the fifth week post-surgery (Fig. S4D-F). These rats also exhibited progressive growth retardation, impaired renal function and elevated blood pressure (Fig. S3D, Fig. S5B-E,G,H). Notably, the rats with MNx developed hyperphosphatemia by the fifth week post-surgery without high-phosphorus diet supplementation (Fig. S5F). Despite greater renal function decline being observed in rats with MNx than in rats with 5/6 Nx, mortality rates in rats with MNx were only marginally higher than those in rats with 5/6 Nx, although not significantly (Fig. S5A). These results demonstrate that MNx not only reduces the time required for UC model establishment but also induces hyperphosphatemia in male SD rats.

### Female SD rats are susceptible to MNx (model 3)

#### Model 3

Previous research indicates that females demonstrate resistance to CKD progression (Cobo et al., 2016); therefore, this study aimed to determine whether MNx could induce UC in female SD rats. Given that LVH manifested in male rats at week 5 post-surgery, echocardiographic assessment commenced at this timepoint (Fig. S4A). Although nephrectomized rats exhibited unchanged LVEF and RWT due to increased diastolic LV diameter, the LV weight to heart weight ratio showed significant elevation. Correspondingly, these rats demonstrated upregulated *Nppa*/*Nppb* expression (Fig. S4B,C, Table S3). The cardiac tissue also exhibited enlarged cardiomyocytes, substantial myocardial fibrosis and increased TdT-mediated dUTP nick-end labeling (TUNEL)^+^ cardiomyocytes (Fig. S4D-F). Compared to males, females experienced higher mortality within 3 weeks post-surgery (Fig. S5A). Nephrectomized females displayed reduced body weight, significant renal impairment, hyperphosphatemia and hypertension. Notably, females exhibited milder renal injury severity compared to males (Fig. S5B-H). These findings confirm that female SD rats can effectively serve as UC models through MNx.

### Male C57Bl/6J and BALB/c mice are not suitable for MNx-induced UC (models 4 and 5)

C57BL/6 and BALC/c represent the most commonly utilized strains in disease modeling (Collins et al., 2007), and we evaluated whether MNx could induce UC in these strains.

#### Model 4

Previous studies demonstrated that male C57BL/6J mice with 5/6 Nx exhibited mild renal injury without developing significant UC (Hamzaoui et al., 2020; Leelahavanichkul et al., 2010). This study investigated whether MNx, which induces more severe renal injury than 5/6 Nx, would result in UC in C57BL/6J mice. Echocardiographic assessment began at week 5 post-MNx (Fig. S6A). However, 12 weeks of continuous monitoring revealed no LVH or altered LVEF in nephrectomized mice (Fig. S6B-E, Table S4). Most mortalities occurred within 2 weeks post-nephrectomy (Fig. S7A), contrasting with male SD rats (Fig. S5A), suggesting acute renal injury rather than CKD progression as the primary cause of death. In the 12th week after surgery, nephrectomized mice exhibited reduced body weight, mild renal injury and marginally increased systolic pressure (Fig. S7B-G). For renal pathology, these mice demonstrated only tubular dilation and glomerular hypertrophy, with minimal interstitial fibrosis (Fig. S7H).

#### Model 5

MNx was performed on male BALB/c mice following the same protocol (Fig. S6A). Similarly, no significant LVH phenotype emerged within 12 weeks post-surgery (Fig. S6B-F, Table S4). Comparable to C57BL/6J mice, most BALB/c mice mortalities occurred within 3 weeks post-nephrectomy (Fig. S7A). Nephrectomized mice exhibited decreased body weight, impaired renal function and mild hypertension (Fig. S7B-G). Renal tissue alterations in BALB/c mice paralleled those observed in C57BL/6J mice (Fig. S7H).

These findings indicate that using MNx in C57BL/6J and BALB/c mice is unsuitable for UC simulation.

### MNx induces UC in male and female CD-1 mice within 5 weeks (models 6 and 7)

#### Model 6

Previous research identified CD-1 as the most susceptible strain to 5/6 Nx-induced CKD (Leelahavanichkul et al., 2010). By week 5 post-MNx (Fig. S8A), male nephrectomized mice demonstrated significant UC phenotypes, including increased LV wall thickness, elevated LV weight to heart weight ratio, upregulated *Nppa*/*Nppb* expression, enlarged cardiomyocytes, LV interstitial fibrosis and increased TUNEL^+^ cardiomyocytes (Fig. S8B-F), accompanied by unchanged LVEF (Table S5). Mortality increased markedly following UC onset (Fig. S9A), with nephrectomized mice exhibiting reduced body weight, substantial renal impairment, hyperphosphatemia and hypertension at week 5 post-surgery (Fig. S9B-H).

#### Model 7

In the fifth week after MNx (Fig. S8A), nephrectomized female CD-1 mice also exhibited significant UC (Fig. S8C-F), with unchanged LVEF (Table S5). Compared to males, females demonstrated lower mortality, particularly during weeks 5-10 post-surgery (Fig. S9A). In addition, nephrectomized females exhibited reduced body weight, renal impairment, hyperphosphatemia and hypertension (Fig. S9B-H). Notably, females displayed less severe renal impairment compared to males.

Collectively, MNx induces phenotypes of CKD and UC in both male and female CD-1 mice, with all subjects exhibiting significant hyperphosphatemia. Therefore, using MNx to induce UC in CD-1 mice effectively reflects the cardiac alterations observed in patients with CKD.

### Balancing renal impairment and nutrition intake is required for adenine-induced UC

The adenine diet represents an alternative approach for UC model development (Leelahavanichkul et al., 2010). However, previous research indicates that although a single-adenine diet can induce CKD, it fails to cause LVH in mice (Chen et al., 2021; Kieswich et al., 2018). We examined whether a 0.25% adenine diet could induce UC in the strain most susceptible to renal injury. Interestingly, a 5-week 0.25% adenine diet (Fig. S10A) caused significant renal injury (Fig. S10H), but mice exhibited no LVH and, instead, developed a smaller left ventricle (Fig. S10B-D, Table S6). Additionally, the 5-week 0.25% adenine diet resulted in marked reduction in body weight and serum albumin (Fig. S10E,F), attributable to decreased food intake (Fig. S10G). Given that nutritional status significantly influences LVH development (Nakamura and Sadoshima, 2018), achieving balance between renal injury and nutritional status became essential, leading to our approach of combining adenine and normal diets to induce UC.

Significantly, brief adenine diet administration, such as a 2-week 0.25% adenine diet (Fig. S11A), induced only temporary renal injury without progression (Fig. S11B-G), confirming previous findings (Okada et al., 1999). Consequently, we developed three feeding protocols to investigate methods for inducing irreversible renal injury (Fig. S12A). By the seventh week, mice receiving the 5-week 0.25% adenine diet demonstrated significant body weight reduction, kidney atrophy, renal impairment, hyperphosphatemia and renal fibrosis (Fig. S12B-I), indicating irreversible renal damage in male CD-1 mice. Notably, only the 5-week adenine diet induced hypertension (Fig. S12J), contrasting with nephrectomized rats that developed hypertension within the first week post-surgery (Fig. S3D).

#### Model 8

As anticipated, transitioning from a 0.25% adenine diet to a normal diet (Fig. S13A) increased body weight and average food intake (Fig. S15A,B), although male mice on the adenine diet began dying in the ninth week (Fig. S15C). LVH without altered LVEF emerged in the seventh week, with elevated cardiac *Nppa/Nppb* expression (Fig. S13B,C, Table S7). Additionally, enlarged cardiomyocytes, cardiac fibrosis and increased TUNEL^+^ cardiomyocytes were observed (Fig. S13D-F). Male CD-1 mice exhibited kidney atrophy, renal dysfunction, hyperphosphatemia, hypertension and renal fibrosis (Fig. S15D-K). Importantly, nutritional status improved compared to that with the adenine-only diet (Fig. S15L).

#### Model 9

We examined whether this protocol could establish UC in female CD-1 mice (Fig. S14A). The transition from 0.25% adenine diet to normal diet restored body weight and average food intake in female CD-1 mice (Fig. S14B,C). However, the adenine group showed no significant LVH even in the ninth week (Fig. S14D-F, Table S8). No mortality occurred during the 10-week feeding period (Fig. S14G). Female CD-1 mice on the 0.25% adenine diet developed kidney atrophy, moderately elevated serum creatine and urea nitrogen, increased systolic blood pressure and renal fibrosis, but neither hyperphosphatemia nor hyperuricemia (Fig. S14H-N). This indicated the requirement for increased adenine concentration.

Subsequently, we implemented a combined 0.3% adenine and normal diet protocol (Fig. S13A). Body weight and average food intake increased significantly following normal diet introduction but decreased in the seventh week (Fig. S15A,B). The adenine groups began experiencing mortality in the eighth week (Fig. S15C). By the seventh week, the adenine group displayed kidney atrophy, impaired renal function, hyperphosphatemia, hypertension, renal fibrosis and mild hypoalbuminemia (Fig. S15D-L). Notably, significant UC phenotypes emerged in the seventh week (Fig. S13B-F, Table S7). Thus, the combination of a 5-week 0.3% adenine diet followed by a 2-week normal diet proved necessary for UC induction in female CD-1 mice.

### Mice with MNx have more prominent phenotypes of UC compared to mice with an adenine diet

Based on the previous nine UC models (Fig. S1), we further compared the phenotypes of mice with MNx- and adenine-induced UC. Notably, although model 6 and model 8 both demonstrated similar levels of body weight reduction, renal impairment, hyperphosphatemia, serum albumin reduction, hypertension and renal fibrosis (Fig. S16F-K; representative images of renal fibrosis shown in Fig. S9H and Fig. S15K) in the seventh week (Fig. S16A), the LVH phenotypes in model 8 were less pronounced than those in model 6. Both models exhibited increased RWT, ratio of LV weight and heart weight, and *Nppa*/*Nppb* expression, although the RWT and *Nppa* expression in model 8 were comparatively lower than those in model 6 (Fig. S16B,C; representative B-mode images shown in Fig. S8B and Fig. S13B). Correspondingly, although both models displayed increased cardiomyocyte cross-section area, model 8 exhibited smaller cardiomyocytes than did model 6 (Fig. S16D; representative images shown in Fig. S8D and Fig. S13D). Moreover, both models demonstrated unchanged LVEF and similar degrees of LV interstitial fibrosis (Fig. S16E and Table S9; representative images of LV fibrosis shown in Fig. S8E and Fig. S13E).

### Major changes of LV tissues in UC

**Fig. 1.**
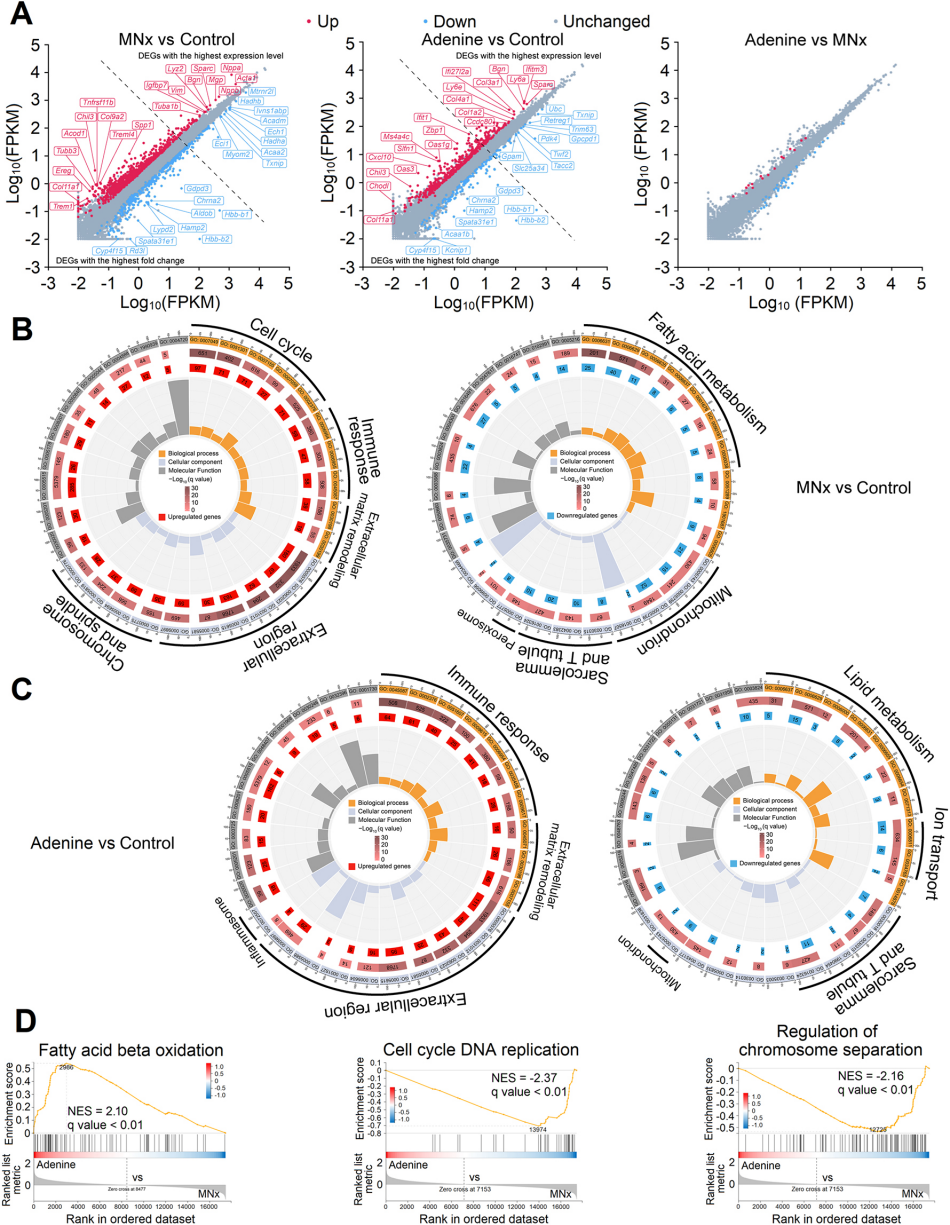
**Global changes of the left ventricle in modified nephrectomy (MNx)- and adenine-induced uremic cardiomyopathy (UC).** (A) Volcano plot of upregulated and downregulated differentially expressed genes (DEGs). FPKM, fragments per kilobase of transcript per million mapped reads. (B,C) Gene Ontology circle graphs showing the main changes in biological process, cellular component and molecular function of MNx-induced UC (B) and adenine-induced UC (C). (D) Gene set enrichment analysis (GSEA) identified the primary differences between MNx- and adenine-induced UC. NES, normalized enrichment score. Control (control group), *n*=5; MNx (modified nephrectomy group), *n*=5; Adenine (0.25% adenine diet group), *n*=5.

The analysis revealed 789 upregulated and 345 downregulated differentially expressed genes (DEGs) in mice with MNx, and the top ten upregulated and downregulated DEGs with the highest fold change and expression level are presented in Fig. 1A. The corresponding proteins of upregulated DEGs in MNx-induced UC were primarily located in the extracellular region, chromosome and spindle, and these proteins were associated with the cell cycle, immune response and extracellular matrix remodeling. Regarding downregulated DEGs in model 6, the corresponding proteins were predominantly located in the mitochondrion, sarcolemma, T-tubule and peroxisome, and these DEGs were primarily associated with fatty acid (FA) metabolism (Fig. 1B).

In comparison to mice with MNx, mice with the adenine diet exhibited fewer DEG changes. Model 8 showed 432 upregulated and 155 downregulated DEGs. The top ten upregulated and downregulated DEGs with the highest fold change and expression level are illustrated in Fig. 1A. Gene Ontology (GO)-cellular component enrichment indicated that the corresponding proteins of upregulated DEGs were primarily located in the extracellular matrix and inflammasome, correlating mainly with immune response and extracellular matrix remodeling. Similarly to mice with MNx, in mice with the adenine diet, downregulated DEGs were primarily associated with lipid metabolism and ion transport, located in the mitochondrion, sarcolemma and T-tubule (Fig. 1C). Notably, the q-values of downregulated DEGs enrichment in model 8 exceeded 0.05, indicating less significant enrichment.

Despite fewer DEGs between model 6 and model 8 (Fig. 1A), the models exhibited 730 and 183 unique DEGs, respectively (Fig. S17B,C). The unique DEGs in model 6 were primarily associated with the cell cycle, T-cell activation and lipid metabolism, whereas those in model 8 primarily correlated with innate immunity (Fig. S17B,C). Similarly, gene set enrichment analysis (GSEA) revealed significantly upregulated FA metabolism-related biological processes and downregulated cell cycle-related processes in model 8 compared to those in model 6 (Fig. 1D; Table S10). Collectively, these findings preliminarily suggest that, despite similar UC phenotypes, the molecular changes in these models differ substantially.

### Cell cycle-related process in UC

**Fig. 2.**
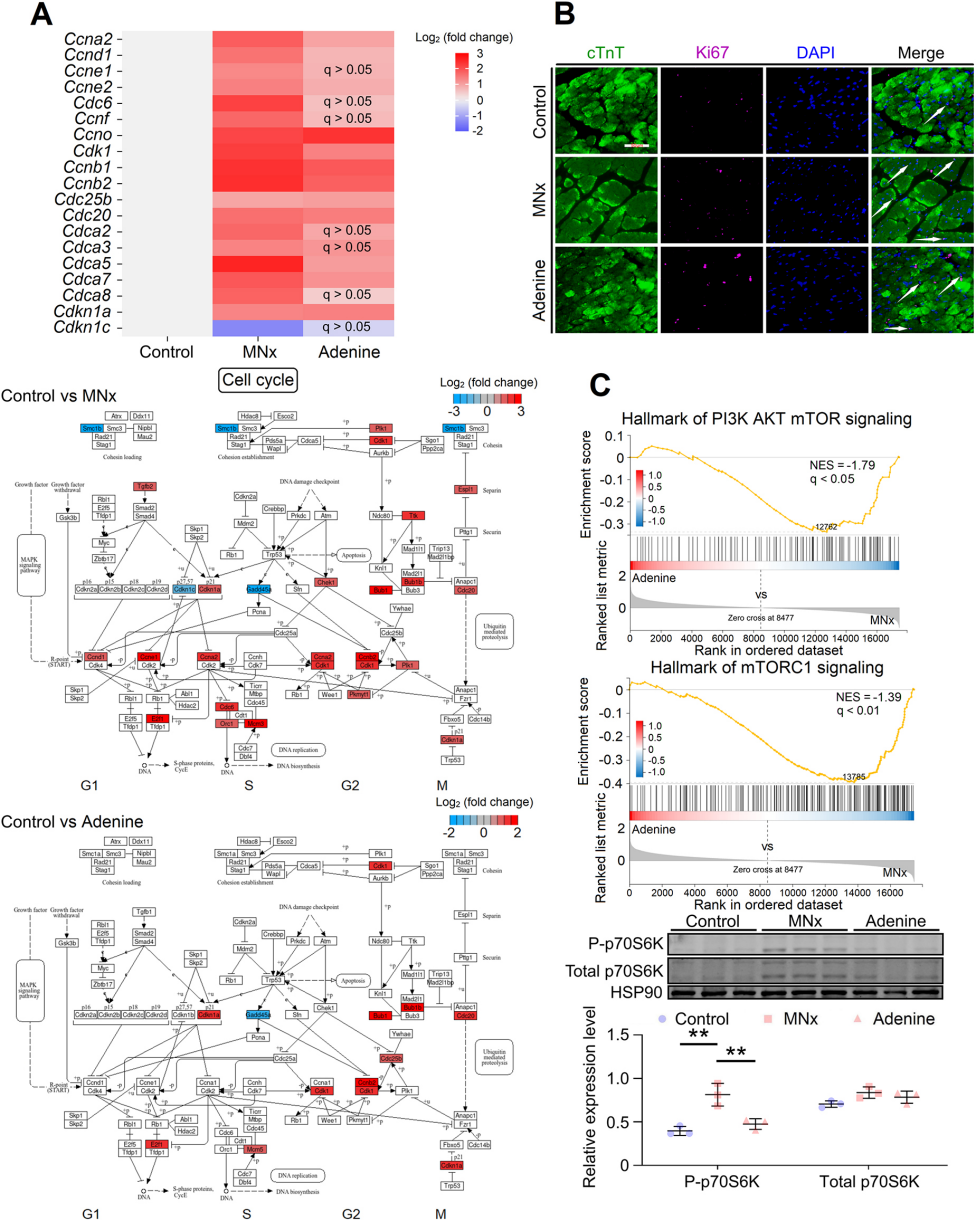
**Analysis of cell cycle processes in UC.** (A) DEGs related to cyclins and cell division cycle associated proteins (top), and the distribution of these DEGs in the cell cycle pathway (bottom). (B) Immunofluorescence showed increased expression of Ki67 in nuclei of cardiomyocytes in UC. White arrows indicate colocalization of Ki67 and cardiomyocyte nuclei (scale bar: 50 μm). (C) As shown by GSEA and western blotting, compared to mice with MNx-induced UC, mice with adenine-induced UC had downregulated PI3K-AKT-mTOR and mTORC1 signaling (for western blotting, *n*=3 for each group). Control, *n*=5; MNx, *n*=5; Adenine, *n*=5. One-way ANOVA followed by Tukey's multiple comparisons test was used. **P<0.01.

Transcriptomic analysis revealed elevated expression of cell cycle- and mitosis-related genes, including *Ccna*, *Ccnb*, *Ccne*, *Ccnd*, *Cdca* and *Cdc*, which were broadly distributed throughout the cell cycle process (Fig. 2A). Quantitative PCR (qPCR) also showed upregulation of cell cycle-related genes, particularly in MNx-induced UC (Fig. S18A), indicating that cardiomyocytes may re-enter the cell cycle during UC progression. Indeed, immunofluorescence demonstrated increased Ki67 expression in nuclei of cardiac troponin T^+^ (cTnT^+^) cardiomyocytes in UC models (Fig. 2B). Notably, *Cdkn1a*, encoding p21, exhibited significant upregulation (Fig. 2A), potentially causing inhibition of cyclin-dependent kinase and mitosis in cardiomyocytes (Li et al., 2020).

Notably, cell cycle-related genes showed minimal changes in model 8 (Fig. 2A), as confirmed by qPCR (Fig. S18A). GSEA revealed that LV tissues in model 8 exhibited downregulated hallmarks of proliferation-related phosphatidylinositol-3-hydroxykinase (PI3K) and mammalian target of rapamycin complex 1 (mTORC1) signaling compared to model 6, while western blot analysis demonstrated downregulated expression of phosphorylated (P)-p70S6K, a downstream target of mTORC1, in model 6 (Fig. 2C). The adenine diet significantly elevated serum adenosine levels (Fig. S18B), and previous research has shown that adenine can activate adenosine 5′-monophosphate-activated protein kinase (AMPK), subsequently inhibiting PI3K-mTORC1 signaling and inducing cell cycle arrest (Steinberg and Hardie, 2023; Su et al., 2020; Young et al., 2015). These findings partially explain the milder LVH phenotypes observed in model 8 than those in model 6 (Fig. S16B-E).

### Metabolic remodeling in UC

**Fig. 3.**
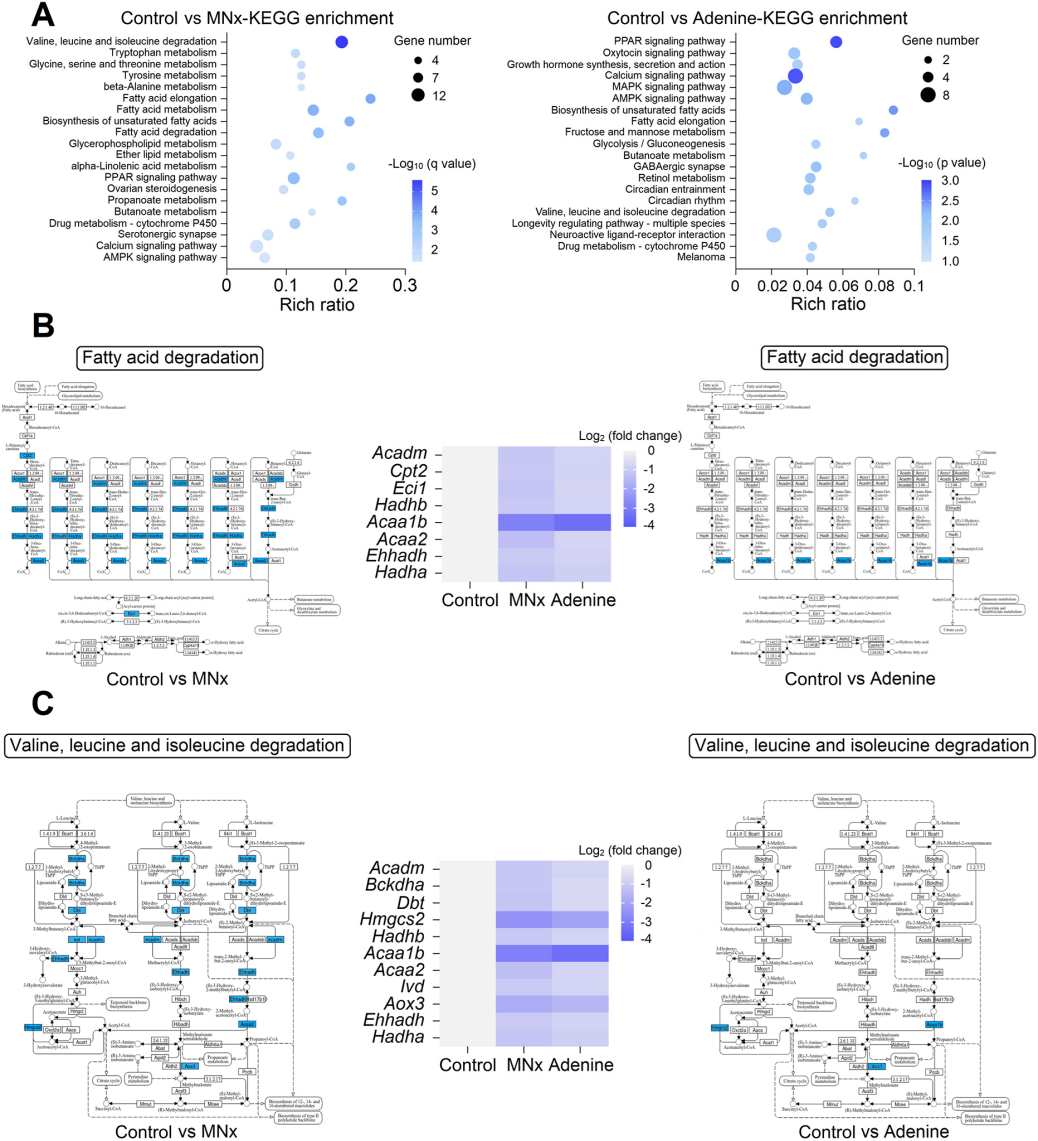
**Analysis of branched-chain amino acid (BCAA) and fatty acid (FA) metabolism.** (A) Kyoto Encyclopedia of Genes and Genomes (KEGG) pathway enrichment of downregulated DEGs in MNx- and adenine-induced UC. (B,C) Downregulated DEGs in FA (B) and BCAA (C) degradation pathways. Control, *n*=5; MNx, *n*=5; Adenine, *n*=5.

Given the clustering of downregulated energetic metabolism-related genes (Fig. 1B,C), further analysis of downregulated DEGs was conducted using Kyoto Encyclopedia of Genes and Genomes (KEGG) enrichment. In model 6, downregulated DEGs were associated with branched-chain amino acid (BCAA) degradation, FA degradation, FA elongation, glycerophospholipid metabolism and amino acid (AA) metabolism (Fig. 3A). In contrast, mice on an adenine diet showed no significant enrichment pathway with q<0.05 in downregulated DEGs, with only decreased FA synthesis, glycolysis and BCAA degradation observable at *P*<0.05 level (Fig. 3A), indicating minor metabolic remodeling in model 8. Specifically, DEGs in model 8 showed fewer changes in FA degradation (Fig. 3B), BCAA degradation (Fig. 3C), FA elongation and glycerophospholipid metabolism (Fig. S19B) than those in model 6 (Fig. S19B), with qPCR confirming the reduced downregulation of genes related to FA degradation and BCAA degradation (Fig. S19A).

**Fig. 4.**
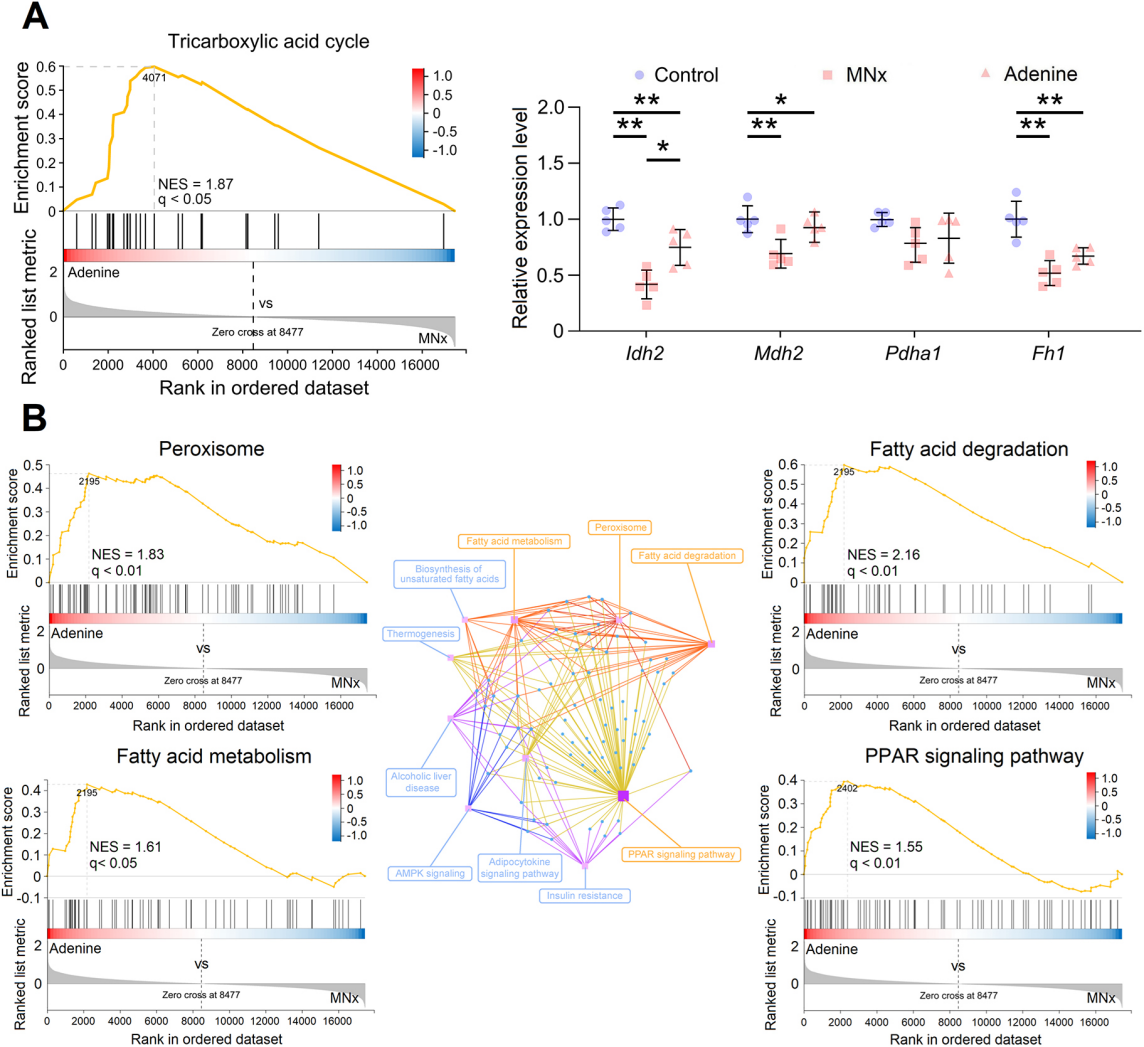
**Analysis of tricarboxylic acid (TCA) and FA metabolism.** (A) Compared to MNx-induced UC, upregulated TCA processes can be found in adenine-induced UC (left). Quantitative PCR confirmed less reduction in expression of TCA-related genes in adenine-induced UC compared to that in MNx-induced UC (right). (B) The network of KEGG pathways indicated the critical role of PPAR signaling in FA metabolism (center), and GSEA indicated upregulated peroxisome, FA degradation, FA metabolism and PPAR signaling in adenine-induced UC compared to MNx-induced UC. Control, *n*=5; MNx, *n*=5; Adenine, *n*=5. One-way ANOVA followed by Tukey's multiple comparisons test was used. *P<0.05, **P<0.01.

The tricarboxylic acid cycle (TCA), central to energetic metabolism, was compared between model 6 and model 8 through GSEA. Mice with MNx-induced UC exhibited downregulated TCA processes compared to those with adenine-induced UC, with qPCR detecting fewer downregulated TCA-related genes in adenine-induced UC (Fig. 4A). Thus, model 8 demonstrated minor metabolic changes compared to model 6. Significantly, GSEA identified upregulated peroxisome proliferators-activated receptor (PPAR) signaling, FA degradation, FA metabolism and peroxisome in model 8 compared to model 6, with these KEGG pathways being closely interconnected (Fig. 4B). As adenine appears to enhance PPAR activity through AMPK activation (Steinberg and Hardie, 2023; Su et al., 2020), it may enhance FA metabolism by upregulating PPAR signaling.

Beyond BCAA metabolism, mice with UC exhibited alterations in glycine, serine, threonine, tyrosine and tryptophan metabolism. Consistent with previous observations, the alterations in AA metabolism were less pronounced in model 8 than those in model 6, as confirmed by qPCR (Figs S20-S22).

### Ion transport in UC

CKD functions as a trigger for proarrhythmic remodeling (Ding et al., 2021; King et al., 2023). Consequently, we examined the expression of ion transporters in LV tissues derived from UC. As illustrated in Fig. S23A, both model 6 and model 8 exhibited reduced cardiac conduction processes, and these models demonstrated widespread alterations in ion transporter expression and their regulators, including decreased expression of *Tbx5*, *Kcnj5*, *Kcnj2* and *Scnb4*, and elevated expression of proarrhythmic *Kcna5* (Fig. S23B) (Tamargo et al., 2009). KEGG network analysis of these cardiac conduction-related genes revealed several pathways potentially related to altered cardiac conduction, including mitogen-activated protein kinase (MAPK), calcium, oxytocin, cAMP and adrenergic signaling (Fig. S23C), suggesting that arrhythmia in CKD can result from disruptions in these pathways.

### LV inflammation in UC

**Fig. 5.**
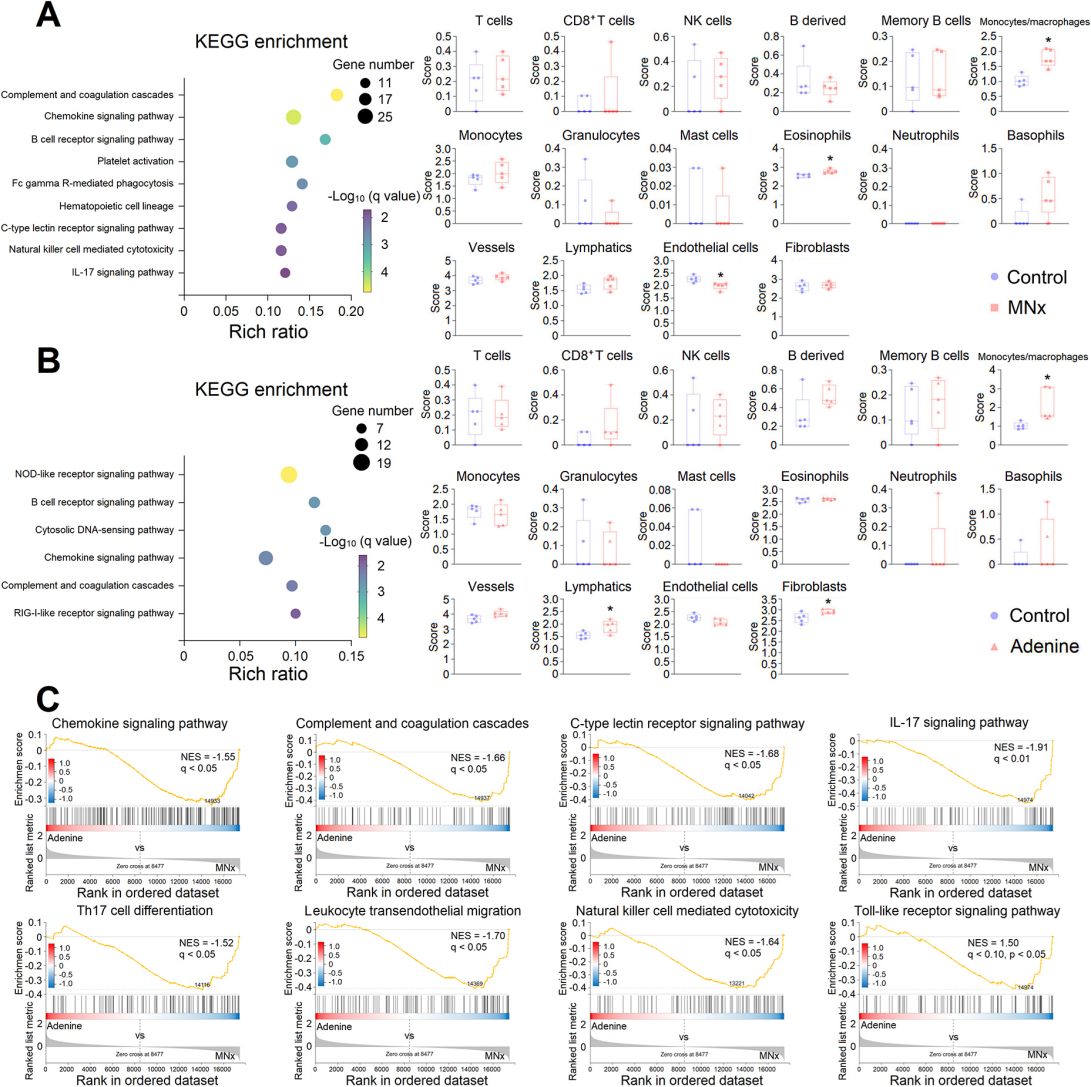
**Analysis of inflammation.** (A) KEGG pathway (immune response) enrichment of upregulated DEGs, and immune infiltration analysis of MNx-induced UC. (B) KEGG pathway (immune response) enrichment of upregulated DEGs, and immune infiltration analysis of adenine-induced UC. (C), GSEA showed downregulated inflammation-related pathway in adenine-induced UC compared to MNx-induced UC. Control, *n*=5; MNx, *n*=5; Adenine, *n*=5. Multiple unpaired *t*-tests were used. *P<0.05.

LV inflammation emerges as another characteristic in model 6 and model 8 (Fig. 1B,C), with immune response-related genes predominantly clustered in upregulated DEGs. Consequently, we conducted KEGG (immune response) enrichment analysis in upregulated DEGs. Compared to the control, mice with MNx exhibited upregulated pathways related to complement, B-cell receptor signaling, C-type lectin receptor signaling and interleukin 17 (IL-17) signaling, while mice treated with adenine demonstrated upregulated pathways related to NOD-like and RIG-I-like receptor signaling, which participate in innate immune response (Fig. 5A,B), aligning with previous results (Fig. S17B,C). Immune infiltration analysis revealed increased monocytes/macrophages, elevated eosinophils and decreased endothelial cells in LV tissues of model 6, while model 8 demonstrated increased infiltration of monocytes/macrophages, lymphatics and fibroblasts (Fig. 5A,B). GSEA indicated that, compared to model 6, model 8 has downregulated chemokine signaling, IL-17 signaling, T-helper 17 (Th17) cell differentiation, leukocyte transendothelial migration and Toll-like receptor signaling (Fig. 5C). We randomly selected several genes related to these inflammatory processes for qPCR verification, confirming that model 8 exhibits downregulated chemotactic process, leukocyte transendothelial migration, IL-17 signaling and Toll-like receptor signaling, while showing upregulated RIG-I-like receptor signaling (Fig. S24). The adenine derivative, adenosine, suppresses Toll-like receptor signaling (Power Coombs et al., 2011), production of proinflammatory factors (Haskó and Pacher, 2012) and leukocyte transendothelial migration (Linden and Cekic, 2012). Therefore, adenine-induced UC may present a distinct cardiac inflammatory response.

### Integrated analysis of UC based on protein-protein interaction (PPI)

**Fig. 6.**
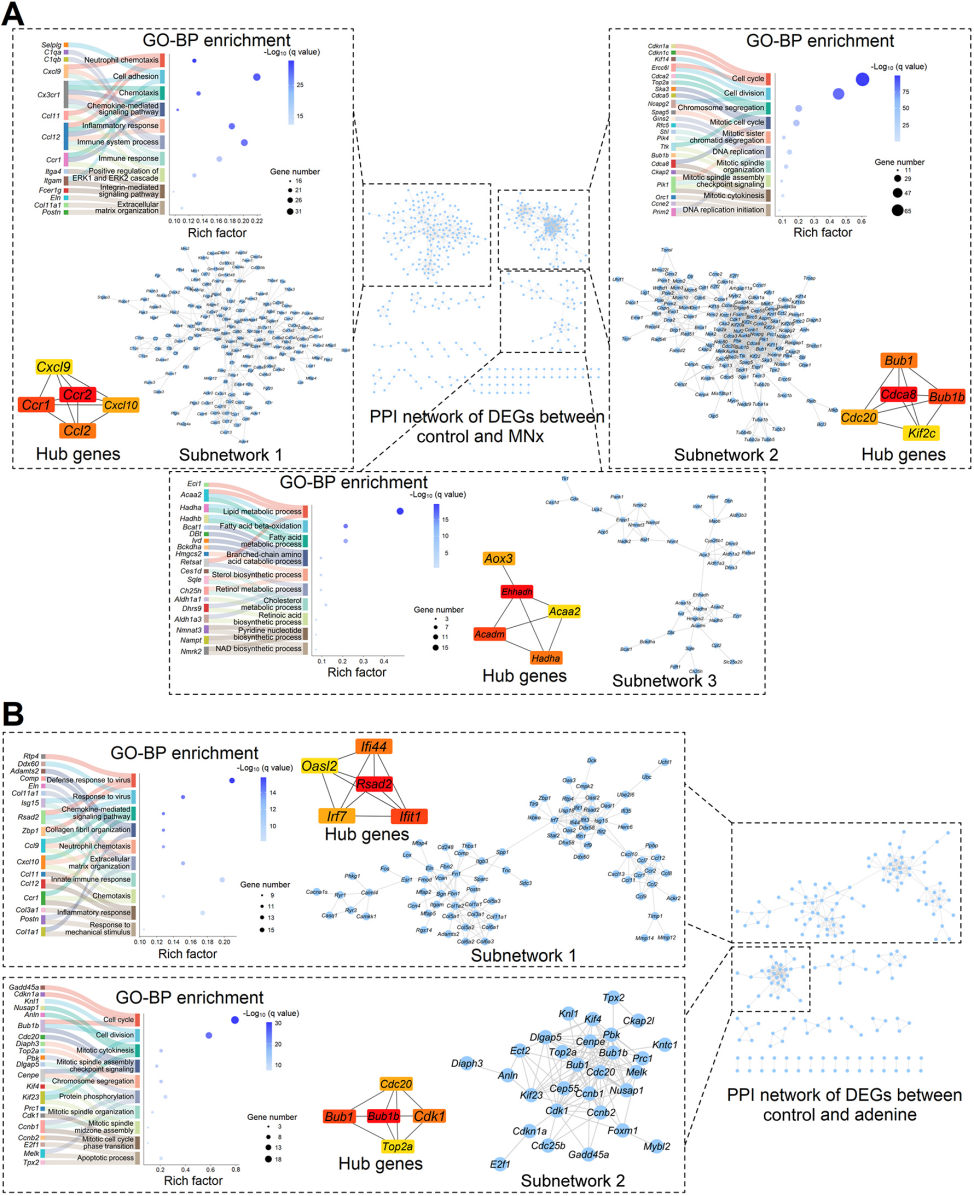
**Protein-protein interaction (PPI) analysis of DEGs in UC.** (A) In MNx-induced UC, the PPI network had three main subnetworks, and these subnetworks were mainly related to immune response, the cell cycle and metabolism. Hub genes graphs show the top five hub genes of each subnetwork. BP, biological process; GO, Gene Ontology. (B) In adenine-induced UC, the PPI network had three main subnetworks, and these subnetworks were mainly related to immune response and the cell cycle. Hub genes graphs show the top five hub genes of each subnetwork. Control, *n*=5; MNx, *n*=5; Adenine, *n*=5.

PPI analysis revealed several main subnetworks in all DEGs. In MNx-induced UC, subnetwork 1 was primarily associated with immune response and extracellular matrix remodeling, subnetwork 2 was primarily associated with cell cycle, and subnetwork 3 was primarily associated with metabolism (Fig. 6A). In adenine-induced UC, subnetwork 1 was primarily associated with immune response and extracellular matrix remodeling, and subnetwork 2 was primarily associated with the cell cycle, although the enrichment degree of subnetwork 2 was lower than that in model 6 (Fig. 6B). These findings highlight the main molecular changes in UC and indicate a minor change in adenine-induced UC.

To identify potential core genes in UC pathogenesis, we identified the top five hub genes in subnetworks through CytoHubba. Alterations in *Ccr2*, *Cdca8* and *Ehhadh* may serve as key drivers in MNx-induced UC pathogenesis, while *Rsad2* and *Bub1b* may be critical in adenine-induced UC (Fig. 6A,B). Because adenine and adenosine may broadly affect cell cycle, metabolism and inflammation processes, based on our previous analysis, we focused primarily on MNx-induced UC in subsequent studies. GSEA enrichment of MNx-induced UC based on WikiPathways revealed upregulation of proliferative pathways [e.g. epidermal growth factor receptor 1 (EGFR-1) signaling] and cell cycle arrest pathway, p53 signaling, further supporting previous analysis. Additionally, downregulated PPAR signaling may constitute the primary cause of energetic metabolism disorders (Table S11).

### C-C-motif receptor 2 (CCR-2) is a primary target for anti-inflammatory and antifibrotic processes in UC

We confirmed the upregulated core genes in subnetwork 1 and subnetwork 2, and the downregulated core genes in subnetwork 3, in MNx-induced UC (Fig. S25). Consequently, we concluded that increased CCR-2 may represent a primary therapeutic target for suppressing cardiac inflammation and fibrosis during UC progression.

**Fig. 7.**
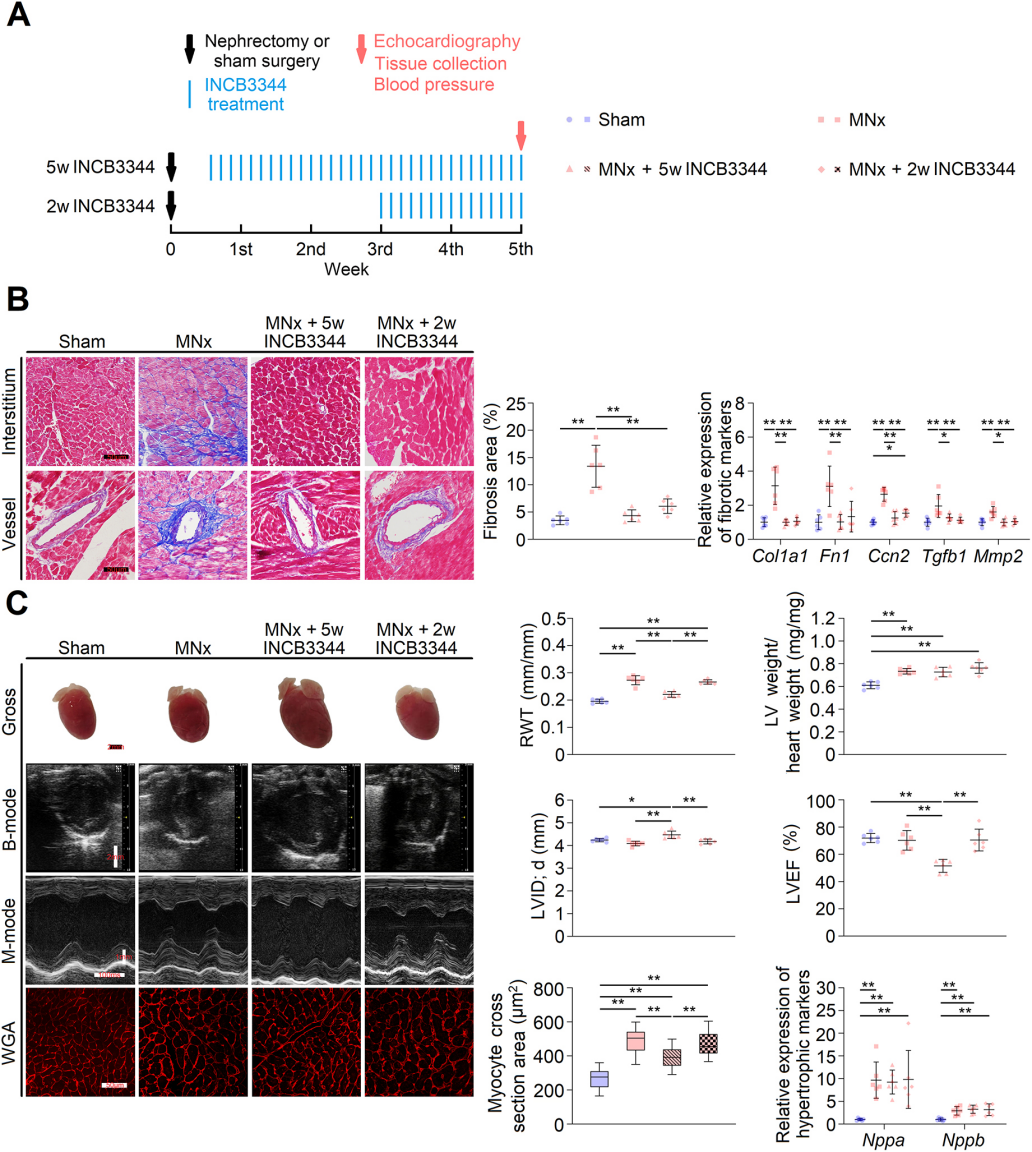
**CCR-2 blockade suppresses left ventricular (LV) fibrosis in MNx-induced UC.** (A) Schedule of INCB3344 administration. w, weeks. (B) CCR-2 inhibition significantly alleviated LV fibrosis (scale bars: 50 μm), and slightly reduced myocyte cross-section area. 2-week CCR-2 treatment obtained similar effects but without causing reduced myocyte cross-section area (scale bars: 50 μm, *n*=360 cells per group). (C) UC model with INCB3344 treatment demonstrated larger heart (Gross, scale bar: 2 mm), slightly reduced LV wall thickness, increased LV diameter [B-mode, scale bar: 2 mm; M-mode, scale bars: 1 mm (longitudinal) and 100 ms (transverse); WGA, scale bar: 50 μm] and compromised LV systolic function. However, 2-week CCR-2 treatment did not induce LV dilation and compromised LV systolic function. Sham (sham group), *n*=6; MNx, *n*=6; MNx+5w INCB3344 (modified nephrectomy group with 5-week INCB3344 injection), *n*=6; MNx+2w INCB3344 (modified nephrectomy group with 2-week INCB3344 injection), *n*=6. One-way ANOVA followed by Tukey's multiple comparisons test or Kruskal–Wallis test followed by Dunn's multiple comparisons test was used. *P<0.05, **P<0.01.

**Fig. 8.**
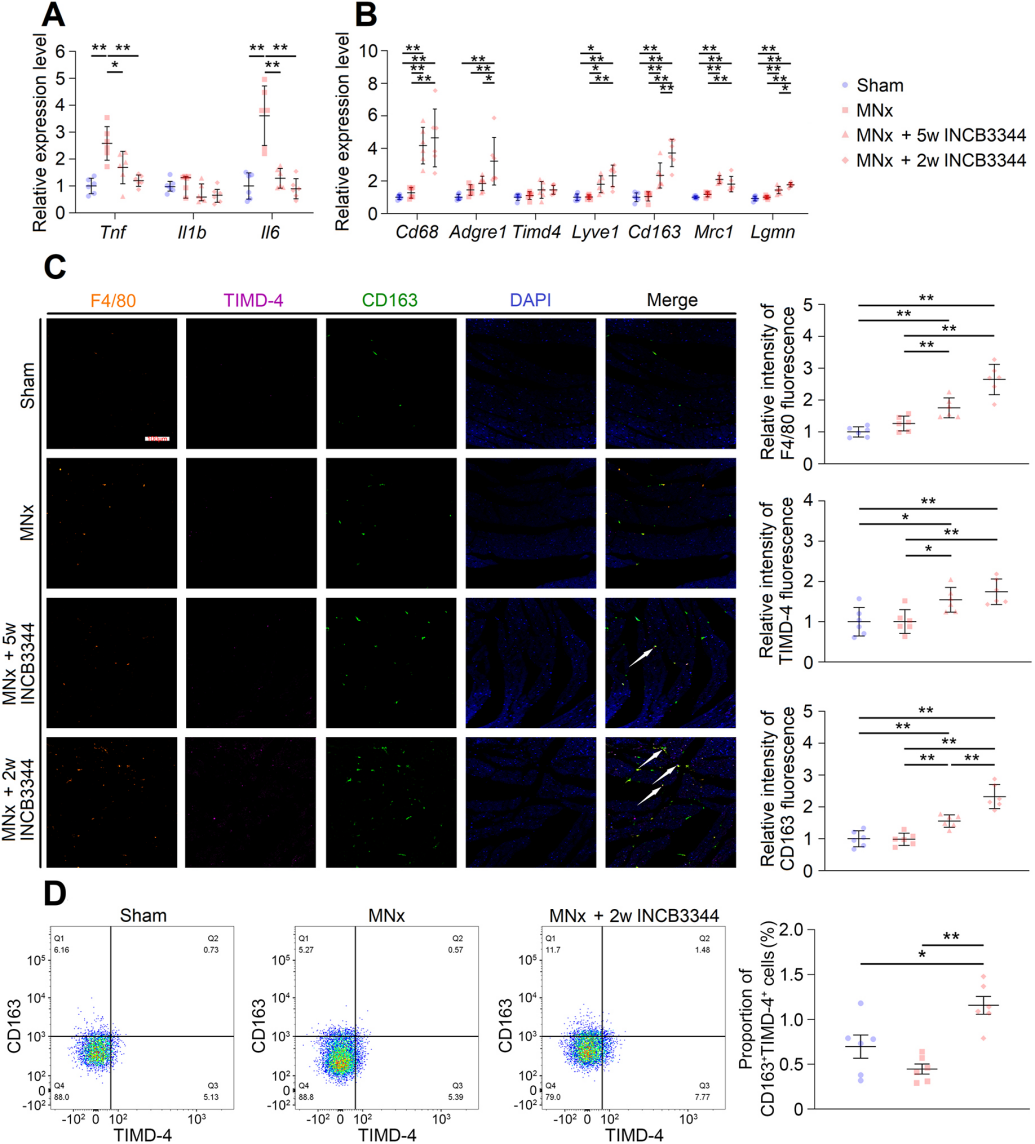
**INCB3344 reduces LV inflammation and may increase LV residual macrophages.** (A) Inhibition of CCR-2 downregulated the expression of markers of inflammation. (B) Inhibition of CCR-2 upregulated CCR-2^−^ residual macrophage markers in LV tissues. (C) Immunofluorescence showed increased infiltration of F4/80^+^, TIMD-4^+^ and CD163^+^ cells in LV tissues after INCB3344 injection, and some F4/80^+^ cells were co-stained with CD163 and TIMD-4. White arrows indicate F4/80^+^TIMD-4^+^CD163^+^ cells (scale bar: 100 μm). (D) Flow cytometry showed that INCB3344 increased the proportion of CD163^+^TIMD-4^+^ cells in F4/80^+^ cells derived from LV tissues. Sham, *n*=6; MNx, *n*=6; MNx+5w INCB3344, *n*=6; MNx+2w INCB3344, *n*=6. One-way ANOVA followed by Tukey's multiple comparisons test or Kruskal–Wallis test followed by Dunn's multiple comparisons test was used. *P<0.05, **P<0.01.

To validate our hypothesis further, we administered INCB3344, a CCR-2 inhibitor, to mice with MNx. Treatment commenced on the fourth day post-surgery and continued until the fifth week (Fig. 7A). Nearly 5 weeks of treatment did not affect renal impairment but marginally reduced systolic pressure (Fig. S26A-F) and effectively suppressed LV CCR-2 expression (Fig. S26G,H). As anticipated, INCB3344 inhibited interstitial/perivascular fibrosis (Fig. 7B) and LV inflammation (Fig. 8A) in mice with MNx. However, although CCR-2 suppression did not significantly affect LVH, it induced LV dilation and decreased LVEF (Fig. 7C; Table S12). Given that MNx rapidly induces hypertension (Fig. S3D), and initial inflammatory and fibrotic processes may be essential for responding to acute hemodynamic overload (Xu et al., 2011), timing the CCR-2 inhibition appropriately is crucial to avoid disrupting normal cardiac response during the acute phase. Subsequently, we initiated INCB3344 administration in the third week after MNx (Fig. 7A). Although the 2-week treatment still had no effect on LVH (Fig. 7C), it reversed cardiac fibrosis (Fig. 7B) and LV inflammation (Fig. 8A), while maintaining LV systolic function without causing LV dilation (Fig. 7C; Table S12). Similar to the 5-week treatment, the 2-week treatment had no effect on renal impairment (Fig. S26A-F) but inhibited LV CCR-2 expression (Fig. S26G,H).

Interestingly, although CCR-2 blockade suppressed inflammatory markers, such as *Tnf* and *Il6* (Fig. 8A), it upregulated *Cd68* and *Adgre1* (encoding F4/80) (Fig. 8B), which are the classical macrophage markers. Further analysis demonstrated increased expression of T-cell immunoglobulin and mucin domain containing 4 (*Timd4*; increasing tendency without reaching a significant difference), *Lyve1*, *Mrc1*, *Cd163* and *Lgmn* (Fig. 8B), which are cardiac CCR-2^−^ residual macrophage markers. Consistently, immunofluorescence and flow cytometry both revealed increased cardiac infiltration of F4/80^+^TIMD-4^+^CD163^+^ cells in mice with MNx after INCB3344 treatment (Fig. 8C,D). Cardiac CCR-2^−^ residual macrophages are considered to prevent fibrosis (Jia et al., 2022; Revelo et al., 2021); thus, the antifibrotic effects of CCR-2 inhibition may not only be mediated by decreasing cardiac infiltration of profibrotic cells (Patel et al., 2018; Xu et al., 2011) but also by promoting the expansion of cardiac residual macrophages.

## DISCUSSION

### UC model establishment

SD, C57BL/6J, BALB/c and CD-1 strains exhibit varying susceptibility to nephrectomy-induced CKD and UC, a factor frequently overlooked by researchers (Bufi and Korstanje, 2022; Hamzaoui et al., 2020; Soppert et al., 2022; Wollenhaupt et al., 2022). MNx accelerated CKD progression in susceptible strains (SD and CD-1) and induced UC more rapidly compared to classical 5/6 Nx, while C57BL/6J and BALB/c strains developed only mild renal injury without obvious UC phenotypes, even after extending the post-surgery periods (Figs S6 and S7). Additionally, hyperphosphatemia correlates with CKD-related cardiovascular complications (Vervloet and van Ballegooijen, 2018), whereas MNx, rather than 5/6 Nx, induces hyperphosphatemia in susceptible strains (Fig. S4F). These findings suggest that the novel MNx effectively simulates UC features observed in human CKD.

Unlike nephrectomy, insoluble 2, 8-dihydroxyadenine (DHA), which is derived from adenine, can deposit in the renal interstitium to induce injury (Klinkhammer et al., 2020). Thus, adenine causes sustained renal injury when feeding mice with an adenine diet. However, the unpalatable nature of the adenine diet leads to reduced food consumption (Fig. S10G, Fig. S14B, Fig. S15A) and severe malnutrition (Fig. S10F), potentially explaining why adenine-fed mice exhibited renal injury but lacked UC in previous studies (Chen et al., 2021; Kieswich et al., 2018; Wollenhaupt et al., 2022; Zhang et al., 2017). Because adequate nutrient supply is a prerequisite for LVH (Nakamura and Sadoshima, 2018), malnutrition may prevent LVH development in the adenine model. Indeed, switching to a normal diet to improve nutrient intake rapidly induces UC phenotypes (Fig. S13), further indicating that nutritional status is crucial for UC pathogenesis.

The phenotypes observed in adenine-induced UC demonstrate milder manifestations compared to those observed in MNx-induced UC. Although this difference might initially be attributed to less severe renal injury from adenine intake, two key observations challenge this interpretation. First, 2-week adenine intake results in ∼3.0-fold elevation in serum creatinine, and 2.7-fold increase in serum urea nitrogen, compared to that in the control group (Fig. S10H), and these indicators further elevate during 5 weeks of adenine intake, reaching ∼4.0-fold increase in serum creatinine, and 5.3-fold increase in serum urea nitrogen, compared to that in the control group (Fig. S16G,H). The MNx model exhibits comparable degrees of renal injury in the fifth week after surgery (Fig. S16G,H). Second, other indicators, including urinary protein excretion, serum phosphate and blood pressure, show similar degrees of change in both MNx and adenine groups (Fig. S16I-K). These observations necessitate consideration of additional factors to explain the minor cardiac molecular alterations in adenine-induced UC. Previous studies indicate that adenosine, a derivative of adenine, directly acts on the myocardium to inhibit cardiac hypertrophy (Fassett et al., 2011; Liao et al., 2003). Thus, although adenine diet induces renal injury, its derivative adenosine may protect myocardium. Such speculation is supported by the observation of significantly elevated serum adenosine (Fig. S18B), minor upregulation of cell cycle-related genes and proliferative signaling (Fig. 2), upregulated PPAR signaling and FA/BCAA metabolism (Figs 3 and 4) in adenine-induced UC. These findings suggest that adenine diet-induced CKD may not be optimal for investigating the pathophysiology of multi-organ complications in CKD owing to the biological effects of adenine.

Notably, ∼50% TUNEL^+^ nuclei are observed in LV myocardium derived from various UC models (Fig. S4F, Fig. S8F, Fig. S13F), exceeding the ∼10% TUNEL^+^ nuclei reported in some studies (Wang et al., 2020). However, other studies have reported higher proportions of TUNEL^+^ nuclei in UC (∼30-50%), with the proportion potentially correlating with renal impairment severity (Ding et al., 2016; Xu et al., 2025). Cardiomyocyte apoptosis analysis using flow cytometry indicates that only ∼10% of cardiomyocytes exhibit varying degrees of apoptosis, although UC models show higher proportions of propidium iodide (PI) single-positive cardiomyocytes (Fig. S27). Several factors may explain the discrepancy between flow cytometry and TUNEL results. First, the elevated proportion of PI-positive cardiomyocytes affects the accuracy of flow cytometry. Second, apoptosis may occur in non-cardiomyocytes (Fig. S4F, Fig. S8F, Fig. S13F). Third, cardiomyocyte hypertrophy is accompanied by increased proportions of binucleated or multinucleated cells (Li et al., 2020), and multiple TUNEL^+^ nuclei may exist in one cardiomyocyte; Therefore, TUNEL^+^ nuclei numbers may not accurately reflect apoptotic cardiomyocyte numbers. Fourth, TUNEL indicates not only apoptotic status but also cells undergoing DNA repair and active gene transcription (Loo, 2011). Future studies would benefit from analyzing cardiomyocyte apoptosis using more precise methods.

### Global molecular changes in UC

Given that adenine or its derivatives may directly influence the myocardium and exhibit biological functions, the molecular changes analysis primarily focused on MNx-induced UC. The main characteristics of UC include upregulation of cell cycle- and inflammation-related genes, and downregulation of FA and AA metabolism (Fig. 1B).

Although traditional understanding is that adult cardiomyocytes lack proliferative capacity, previous studies indicate that these cells can partially re-enter the cell cycle but fail to complete mitosis for full cell proliferation, resulting in cardiomyocyte hypertrophy (Ahuja et al., 2007; Broughton and Sussman, 2019; Cardoso et al., 2020; Li et al., 2020; Magadum et al., 2020). Indeed, our study also reveals increased expression of Ki67 in cardiomyocyte nuclei in mice with UC (Fig. 2B). One study demonstrates that angiotensin-II-induced cardiomyocyte hypertrophy correlates with increased multinucleation and S/G_2_ phases, while cell cycle inhibitors (p21 and p27) peak during late-stage hypertrophy, indicating post-G_2_ phase division suppression (Li et al., 2020). Cell proliferation requires AAs and nucleotides (Zhu and Thompson, 2019), and mice with UC exhibit decreased FA oxidation and modified AA metabolism (Fig. 3; Figs S20-S22), which is termed as metabolic remodeling (Nguyen and Schulze, 2023). Although mitochondrial dysfunction may explain reduced FA and BCAA oxidation in UC (Taylor et al., 2015), this could represent an adaptive response to preserve substrates for cell cycle re-entry as well. As FAs serve as the primary energetic substrate for cardiomyocytes, reduced FA oxidation results in energy deficiency and subsequent downregulation of energy-intensive ion transporters (Fig. S23). Consequently, CKD-related arrhythmia susceptibility likely stems from decreased energy availability (Stewart et al., 2005). Several pathways potentially mediate these processes: proliferative pathways including EGFR-1 signaling, MAPK signaling and focal adhesion-PI3K signaling, alongside the cell cycle arrest pathway, p53 signaling (Table S11). These pathways synergistically promote cardiomyocyte proliferation while suppressing division, leading to LVH, and reduced PPAR signaling downregulates FA oxidation, conserving substrates for cardiomyocyte proliferation (Table S11).

Inflammation plays a crucial role in cardiac remodeling, although the specific immunologic mechanisms in UC remain incompletely understood. Previous studies highlight the significance of T-cell and macrophage infiltration in UC-related diastolic dysfunction and cardiac fibrosis (Nakano et al., 2021; Winterberg et al., 2019), with proinflammatory macrophage infiltration being central to the compensatory-to-decompensatory transition in pressure overload-induced LVH (Ren et al., 2020). Owing to bulk RNA-sequencing resolution limitations, our findings only revealed increased monocyte-derived macrophage [predominantly CCR-2^+^ macrophages (Zaman et al., 2021)] infiltration (Fig. 5A), which may significantly influence UC progression. Notably, we identified elevated IL-17 signaling in MNx-induced UC (Fig. 5A), consistent with the known role of IL-17 in promoting cardiac fibrosis in ischemic heart failure (Chang et al., 2018). This suggests IL-17 and Th17 cells as potential therapeutic targets for LV fibrosis in UC.

### Adenosine as a potential therapeutic for UC

Although adenine converts to DHA, causing renal injury (Klinkhammer et al., 2020), it also transforms into adenosine AMP. It is possible that adenosine/AMP, rather than adenine, activates AMPK signaling (Young et al., 2015), which inhibits PI3K-mTORC1 signaling to induce cell cycle arrest (Steinberg and Hardie, 2023; Su et al., 2020; Young et al., 2015) and enhances FA metabolism through PPAR activation (Steinberg and Hardie, 2023; Su et al., 2020). Our research indeed reveals suppression of mTORC1 signaling in model 8 (Fig. 2C). Additionally, adenosine inhibits Toll-like receptor signaling (Power Coombs et al., 2011), proinflammatory factor production (Haskó and Pacher, 2012) and leukocyte transendothelial migration (Linden and Cekic, 2012), which are downregulated in model 8 compared to model 6 (Fig. 5C; Fig. S24). Given the conversion of adenosine to AMP, adenosine presents a promising multi-target therapeutic approach for UC.

Significantly, adenine-induced UC exhibits enhanced innate immune response (Fig. 5B; Fig. S17C), potentially due to myocardial DHA deposit, which causes renal injury in adenine-induced nephropathy (Okabe et al., 2013). However, myocardial DHA deposits were not observed (Fig. S28). Furthermore, adenine-induced UC shows upregulated RIG-I-like receptor signaling (Fig. 5C,D), although the mechanism by which adenine affects this RNA-sensing innate immune response remains unclear. Further investigation into adenine's immunological effects warrants attention.

### CCR-2 is a core target of antifibrosis in UC?

Fibrosis and inflammation demonstrate strong interconnection through PPI analysis, with CCR-2 emerging as a rank 1 hub gene in the inflammation/fibrosis subnetwork (Fig. 6A), suggesting a crucial role for CCR-2 in UC fibrosis. CCR-2 functions as a receptor for monocyte chemoattractant protein 1 (MCP-1), and the MCP-1/CCR-2 axis facilitates monocyte recruitment (França et al., 2017). Notably, mice with UC exhibit increased monocyte/macrophage infiltration (Fig. 6A). Monocyte-derived macrophages serve as initial immunocytes following injury, activating fibroblasts and promoting infiltration of other immune cells to induce inflammation and fibrosis across various organs (Krenkel et al., 2018; Mercer et al., 2009; Reich et al., 2013; Revelo et al., 2021). Therefore, the MCP-1/CCR-2 axis presents an ideal target for attenuating progressive inflammation and fibrosis in UC. As anticipated, sustained CCR-2 inhibition substantially reverses interstitial and perivascular fibrosis (Fig. 7B), while having no impact on LVH (Fig. 7C). These findings align with previous research demonstrating absence of cardiac fibrosis in angiotensin-II-treated *Ccr2*^−/−^ mice (Xu et al., 2011). Furthermore, these results also confirm that fibrosis and hypertrophy represent distinct pathological processes in UC, as indicated by minimal connection between the fibrosis/inflammation subnetwork and cell cycle subnetwork (Fig. 6A), while the cell cycle subnetwork mainly relates to LVH (Ahuja et al., 2007; Broughton and Sussman, 2019; Cardoso et al., 2020; Li et al., 2020; Magadum et al., 2020).

Initiating CCR-2 inhibition in the fourth day after MNx results in LV dilation and compromised LV systolic function (Fig. 7C; Table S12), with similar effects observed in *Ccr2*^−/−^ mice administered angiotensin-II (Xu et al., 2011). The reduced cross-section of cardiomyocytes and wall thickness likely result from LV dilation (Fig. 7C), which stretches muscle fibers. The mechanism by which CCR-2 inhibition leads to LV dilation and impaired LV function remains unclear. Inflammation may provide beneficial effects during early cardiac injury stages (Adamo et al., 2020), and appropriate inflammatory responses potentially enhance cardiomyocyte contractility in response to stimuli (Besse et al., 2022). Macrophages in normal heart and early response to transverse aortic constriction (TAC) (0-2 weeks after TAC) associate with heart contraction, action potential and angiogenesis, while their functions shift toward immune response, tissue remodeling and angiogenesis during middle-stage TAC (2-5 weeks after TAC). Therefore, inhibiting macrophage activation at middle-stage rather than early-stage TAC mitigates cardiac fibrosis (Ren et al., 2020). A recent study further indicates that CCR-2^+^ macrophages play a critical role in eliminating damaged mitochondria and improving ATP generation in myocardium during early heart failure with preserved ejection fraction (HFpEF) (Raman et al., 2025). Because enhanced cardiac ATP generation maintains systolic function and LV wall tension in response to stimuli, premature inhibition of CCR-2^+^ macrophage functions may trigger LV dilation. In rats with MNx, increased LV wall thickness and decreased LV diameter in the first to third week post-surgery maintain LV wall tension for elevated blood pressure, although these changes occur without increased LV weight and heart weight (Fig. S3C,D). Based on these findings and those by Ren et al. (2020), 0-3 weeks post-MNx appears to represent an early UC stage. Indeed, commencing INCB3344 treatment in the third week after surgery effectively reverses fibrosis (Fig. 7B) without inducing LV dilation or systolic function impairment (Fig. 7C; Table S12).

Although CCR-2 inhibition improved LV inflammation (Fig. 8A), it enhanced macrophage expansion, as evidenced by increased expression of macrophage markers CD68 and F4/80 (Fig. 8B). Given that cardiac residual CCR-2^−^ macrophages demonstrate anti-inflammatory and antifibrotic properties (Jia et al., 2022; Revelo et al., 2021), we hypothesized that CCR-2 inhibition not only prevents monocyte-derived macrophage infiltration but also promotes residual macrophage expansion to suppress fibrosis. Indeed, elevated expression of CCR-2^−^ residual macrophage markers (*Timd4*, *Lyve1*, *Cd163*, *Mrc1* and *Lgmn*) and increased infiltration of F4/80^+^TIMD-4^+^CD163^+^ cells were observed in UC mice treated with INCB3344 (Fig. 8B,C). These results are consistent with those from a recent study, which shows increased TIMD-4^+^ macrophage infiltration in *Ccr2^−/−^* mice with HFpEF (Raman et al., 2025). Notably, the 2-week INCB3344 treatment appeared to induce greater cardiac residual macrophage expansion compared to the 5-week treatment (Fig. 8B,C). Even so, the mechanisms underlying CCR-2 inhibition-induced residual macrophage expansion remain unclear and warrant further investigation.

Several limitations exist in our study. First, bulk transcriptome analysis provides lower resolution than single-cell transcriptome analysis, limiting our ability to obtain detailed transcriptome landscapes of individual cell subsets. Second, owing to the difficulty in obtaining human LV tissues from patients with CKD, we cannot compare our mouse UC transcriptomic landscape with that of human UC. Thus, our future research will compare MNx-induced UC with Alport syndrome (*Col4a3*^−/−^ mice)-induced UC, which more closely resembles human UC (Dussold et al., 2019). Third, although female mice with MNx exhibit UC phenotypes, INCB3344 administration in subsequent studies was limited to male mice. Fourth, flow cytometry, which is primarily used to validate immunofluorescence findings, cannot definitively confirm F4/80^+^CD163^+^TIMD-4^+^ cells as CCR-2^−^ residual macrophages.

In summary, this study elucidates strain-, sex- and protocol-based variations in the UC model and introduces two novel methods for effectively modeling UC in rodents. Additionally, we present a transcriptomic landscape revealing global molecular alterations in UC, encompassing disrupted cell cycle, FA metabolism, cardiac conduction, extracellular matrix remodeling and inflammatory response. Furthermore, CCR-2 emerges as a novel therapeutic target for fibrotic and inflammatory processes in UC. *In vivo* studies demonstrate that appropriate CCR-2 inhibition, administered without compromising normal cardiac response during the acute phase, effectively reverses LV fibrosis in UC.

## MATERIALS AND METHODS

### Animals

Male 8-week-old SD rats (Laboratory Animal Center, Southern Medical University) were used for model 1 and model 2, and female 8-week-old SD rats (Laboratory Animal Center, Southern Medical University) were used for model 3. Male 8-week-old C57BL/6J mice (Laboratory Animal Center, Southern Medical University) and 8-week-old BALB/c mice (Laboratory Animal Center, Southern Medical University) were used for model 4 and model 5, respectively. Male 8-week-old CD-1 mice (Beijing Vital River Laboratory Animal Technology Co., Ltd.) were used for model 6 and model 8, and female 8-week-old CD-1 mice (Beijing Vital River Laboratory Animal Technology Co., Ltd.) were used for model 7 and model 9. All animals were maintained in a specific pathogen-free facility under standard temperature (22±2°C), humidity (50-60%) and light (12-h light/12-h dark cycle) conditions, with food and water provided *ad libitum*. The animal experiments were approved by the Animal Ethics Committee of Nanfang Hospital (license number NFYY-2020-0113).

### Nephrectomy-induced UC model (models 1-7)

Rats and mice underwent a 1-week acclimation period before nephrectomy. The nephrectomy was performed as a two-step procedure. The animals were anesthetized with 5.0% isoflurane (vol/vol) in 600 cm^3^/min air flow via inhalation for induction and maintained under 1.5-2.5% isoflurane via a nose mask throughout surgery. Anesthetic depth was assessed by absence of toe pinch response, and body temperature was maintained using a heating pad. Following anesthesia, the right back skin was shaved, disinfected with 75% alcohol and incised along with muscular tissues to expose the right kidney. The adrenal gland was separated from the kidney, followed by dissection of perihilar adipose tissue. In the first stage, only two poles of the right kidney were removed, with excision ranges for 5/6 Nx and MNx shown in Fig. S3A. The kidney wound was covered with sterile gelfoam to achieve hemostasis, and the muscular and skin incisions were continuously sutured. One week later, the entire left kidney was removed after ligating the renal pedicle with silk under similar anesthetic conditions, followed by continuous suturing of the incisions. The sham group underwent identical procedures without kidney removal.

### Adenine-induced UC model (models 8 and 9)

Mice underwent 1-week acclimation feeding, followed by a 3-day transitional period with mixed adenine and normal chow before receiving adenine chow diet (Trophic Animal Feed High-Tech Co., Ltd.). Specifically, the adenine to normal chow ratio was 1:2 on day 1, 1:1 on day 2 and 2:1 on day 3, with pure adenine chow provided from day 4. The adenine groups received various adenine-containing chows for different durations, with detailed intake schemes described in the corresponding sections. Control groups received standardized control diet (Trophic Animal Feed High-Tech Co., Ltd.) and water *ad libitum* throughout the experimental period. Key dietary information is provided in Table S1.

### INCB3344 treatment

CCR-2 antagonist INCB3344 (TargetMol Chemicals Inc.; TQ0103) was dissolved in dimethyl sulfoxide, and 5% volume of this solution was combined with 95% volume of saline containing 20% sulfobutyl ether-β-cyclodextrin (TargetMol Chemicals Inc.; T16858). The solution underwent ultrasonic agitation for 15 min to ensure complete dissolution. Four days post-MNx, male mice with MNx were randomly assigned to receive either INCB3344 (30 mg/kg) or vehicle (saline solution of 5% dimethyl sulfoxide and 20% sulfobutyl ether-β-cyclodextrin) via daily intraperitoneal injections. For the 2-week INCB3344 treatment, mice with MNx received daily intraperitoneal administration of INCB3344 (30 mg/kg) beginning in the third week after surgery. In the fifth week after MNx, echocardiography and blood pressure measurements were performed, followed by tissue collection after sacrifice.

### Transthoracic echocardiography

High-resolution echocardiography (MS 250 13-24 MHz for rats and MS 400 18-38 MHz for mice) was used to evaluate the left ventricle through Vevo 2100 Linear Array Imaging (FUJIFILM VisualSonics, Inc.). Echocardiographic analyses were conducted at specific time points under isoflurane anesthesia. Anesthesia induction was achieved using 5.0% isoflurane (vol/vol) in 600 cm^3^/min air flow via inhalation, while maintenance was sustained with 0.5-1.5% isoflurane via nose mask delivery. Heart rates were maintained at 450-550 beats/min for mice and 300-400 beats/min for rats during detection. Recording was performed in B-mode and M-mode (short axis view, papillary muscle level), with M-mode images used to measure LV wall thickness, diameter and ventricular function. A VevoLab 2.1.0. (FUJIFILM VisualSonics, Inc.) was employed to analyze diastolic LV anterior wall thickness (LVAW; d), diastolic LV posterior wall thickness (LVPW; d), diastolic LV diameter (LVID; d) and left ventricle end-diastolic volume (LV vol; d). Additionally, systolic LV anterior wall thickness (LVAW; s), systolic LV posterior wall thickness (LVPW; s), systolic LV diameter (LVID; s), and left ventricle end-systolic volume (LV vol; s) were measured. These measurements averaged a minimum of three consecutive cardiac cycles per animal. LVEF was calculated as LV vol; s/LV vol; d×100%, and LV fractional shortening as (LV vol; d−LV vol; s)/LV vol; d×100. LV mass was calculated as 1.053×[(LVID; d+LVPW; d+LVAW; d)^3^−LVID; d^3^]×0.8. The RWT was determined by LVAW; d/LVID; d.

### Blood pressure measurement

Blood pressure was measured noninvasively using a BP2000 Series II Blood Pressure Analysis System (Visitech Systems, USA) while animals were awake. The tails were secured in a specialized cuff connected to the detection system. All measurements were conducted in the afternoon, with the first ten detection values discarded to minimize stress-induced blood pressure fluctuations. For each rodent, ten stable measurements of systolic and diastolic pressures were obtained and averaged.

### Blood and urine sampling

Upon reaching endpoints, rats and mice were euthanized using 5% isoflurane anesthesia in a separate room from the housing facility. Blood was collected through eyeball extirpation and stored at 4°C for serum precipitation, followed by centrifugation at 4500 ***g*** for 5 min to maximize serum collection. Serum creatinine, urea nitrogen, phosphate and albumin were analyzed using an AU480 Clinical Chemistry Analyzer (Beckman Coulter, Inc.). Urine samples were collected using metabolism cages (DXL-X and DXL-D, Beijing Jiayuan Industrial Technology Co., Ltd.) and centrifuged at 12,000 ***g*** and 4°C for 10 min to obtain the supernatant. The urine supernatant was analyzed for protein/creatinine ratio using a Catalyst One Chemistry Analyzer (IDEXX Laboratories, Inc).

### Serum adenosine assay

Serum adenosine concentration was determined using an Adenosine Assay Kit (Cell Biolabs, Inc.; MET-5090) following the manufacturer's protocol.

### Histological analysis of renal and cardiac tissues

Following sacrifice, body weight, total cardiac mass and renal mass (only in the adenine model) were measured. Renal and cardiac tissues were immediately immersed in 10% neutral formalin for 48 h at 4°C, and the fixed tissues were sequentially dehydrated in ascending alcohol concentrations, xylene and paraffin. Subsequently, tissues were embedded in paraffin and sectioned to 3 μm thickness using a HistoCore BIOCUT R (Leica Microsystems Co., Ltd.). For tissue staining, paraffin tissue slides were sequentially rehydrated in descending alcohol concentrations and water. A Masson Trichrome Stain Kit (Beijing Solarbio Science & Technology Co., Ltd.; G1340) was utilized to visualize renal and cardiac fibrosis. Specifically, slides were stained with Ponceau-Acid Fuchsin Solution for 4 min, followed by water rinsing. The slides were then incubated with Phosphomolybdic Acid Solution for 3 min and rinsed with water. Finally, slides were stained with Aniline Blue Solution for 4 min and rinsed with water. Dried slides were mounted with neutral resins (Beijing Solarbio Science & Technology Co., Ltd.; G8590) and observed using a BX53 Microscope (Olympus Co., Ltd.). The cardiac fibrosis area percentage was determined in a masked manner by analyzing five randomly selected 20× fields of each slide through ImageJ software (National Institutes of Health).

### Wheat germ agglutinin (WGA) staining

Following rehydration of cardiac paraffin slides, sodium citrate solution was used to fix slides at 95°C for 10 min, followed by incubation with QuickBlock Blocking Buffer for Immunol Staining (Beyotime Biotechnology Co., Ltd.; P0220) for 30 min. Subsequently, slides were incubated with WGA conjugated to Alexa Fluor 633 (Thermo Fisher Scientific, Inc.; W21404) at 50 μg/ml. Immunofluorescence images were captured using an LSM 880 (Carl Zeiss Microscopy, LLC). The myocyte cross-sectional area was measured using ImageJ software, with at least 60 cells measured per slide.

### TUNEL analysis

Cardiomyocyte apoptosis was detected using TUNEL assay with the In Situ Cell Death Detection Kit, POD (Roche Applied Science; 11684817910). Following rehydration, the cardiac tissue slides were incubated with 20 μg/ml proteinase K (Beyotime Biotechnology Co., Ltd.; ST533) for 20 min at 37°C, and TUNEL reaction mix was applied for 60 min at 37°C after phosphate buffer solution (PBS) wash. The slides were then incubated with converter-POD solution from the kit for 30 min at 37°C and DAB substrate (Beyotime Biotechnology Co., Ltd.; P0202) sequentially. After PBS washing, the slides were counterstained with Hematoxylin for 2 min, and images were captured using a BX53 (Olympus Co., Ltd.). The percentage of TUNEL^+^ nuclei relative to total nuclei was determined in a masked manner by analyzing five randomly selected 40× fields per slide.

### Immunofluorescence and immunochemistry

A TSA Fluorescence Triple Staining Kit (ABclonal Technology; RK05903) was utilized to perform immunofluorescence for F4/80 CD163, and TIMD-4. LV tissue slides were fixed in sodium citrate solution at 95°C for 20 min following rehydration, then incubated with peroxidase blocking buffer (ZSGB-BIO Co.; ZLI-9311) for 15 min. After PBS washing, the slides were incubated with QuickBlock Blocking Buffer for Immunol Staining (Beyotime Biotechnology Co., Ltd.; P0220) for 30 min. The slides were incubated overnight at 4°C with antibodies against TIMD-4 (1:200; Proteintech Group, Inc.; 12008-1-AP), followed by incubation with horseradish peroxidase (HRP)-linked goat anti-rabbit/mouse secondary antibodies at room temperature for 50 min after PBS washing. Subsequently, slides were incubated with TYR-690 fluorochrome at room temperature for 10 min after PBS washing, and antibodies were eluted using sodium citrate solution at 95°C for 30 min. This process was repeated for antibodies against F4/80 (1:800; Proteintech Group, Inc.; 28463-1-AP) and CD163 (1:1000; Starter Biotechnology Co., Ltd.; S0B2194), using TYR-570 and TYR-520 fluorochromes, respectively. Slides were mounted using antifade mounting medium containing DAPI (Biosharp Life Sciences; BL739A), and immunofluorescence images were captured using the LSM 880. The relative fluorescence intensity was determined in a masked manner by analyzing five randomly selected 20× fields per slide using ImageJ software.

For evaluating colocalization of cTnT and Ki67, the TSA Fluorescence Triple Staining Kit was employed following similar procedures. After fixation and blocking steps, slides were incubated overnight at 4°C with antibodies against Ki67 (1:500; Abcam; ab15580), followed by HRP-linked goat anti-rabbit secondary antibodies at room temperature for 50 min after PBS washing. TYR-570 fluorochrome was applied for 10 min at room temperature after PBS washing, followed by antibody elution with sodium citrate solution at 95°C for 30 min. This process was repeated for antibodies against cTnT (1:500; Abcam; ab209813), using TYR-520 fluorochrome. Slides were mounted with antifade mounting medium (containing DAPI; Biosharp Life Sciences; BL739A) and imaged using the LSM 880.

For CCR-2 analysis, LV tissue slides were fixed in EDTA pH 9.0 solution at 95°C for 10 min after rehydration, followed by incubation with peroxidase blocking buffer (ZSGB-BIO Co.; ZLI-9311) for 15 min. After PBS washing, slides were incubated with QuickBlock Blocking Buffer for Immunol Staining (Beyotime Biotechnology Co., Ltd.) for 30 min. Antibodies against CCR-2 (1:100; Proteintech Group, Inc.; 16153-1-AP) were applied overnight at 4°C, followed by incubation with goat anti-rabbit IgG (ZSGB-BIO Co.; PV-6000) for 30 min. Nuclei were stained with Hematoxylin. The average optical density was determined in a masked manner by analyzing five randomly selected 40× fields per slide.

### RNA extraction and qPCR analysis

LV tissues underwent RNA extraction using an Animal Total RNA Isolation Kit (Foregene Co., Ltd.; RE-03011). Briefly, 20 mg of LV tissues were immersed in lysis buffer and homogenized with steel beads using TissueLyser-II (Qiagen, Inc.). The supernatant was then filtered through the DNA-Cleaning Column and RNA-Only Column sequentially to remove genomic DNA and isolate RNA. The obtained RNA was washed with desalting buffer and eluted with ddH_2_O. RNA quantification and purity were assessed using a NanoDrop 2000 Spectrophotometer (Thermo Fisher Scientific, Inc.), and RNA solutions were standardized to 50 ng/μl. Subsequently, 50 ng RNA was converted to complementary DNA using a HiScript III 1st Strand cDNA Synthesis Kit (Vazyme Biotech Co., Ltd.; R312) with Oligo dT/Random hexamers mixed primers. An Applied Biosystems Veriti Thermal Cycler was programmed at 37°C/15 min-85°C/10 s for reverse transcription. mRNA levels were determined using ChamQ SYBR Color qPCR Master Mix (Low ROX Premixed; Vazyme Biotech Co., Ltd.; Q431) on a 7500 Fast Real-Time PCR System (Thermo Fisher Scientific, Inc.), with reaction conditions set at 95°C/10 s-60°C/30 s for 40 cycles. Relative mRNA expression was calculated using the 2^–ΔΔCt^ method with *Rn18s* as normalization. Primers are listed in Table S2.

### RNA sequencing

RNA sequencing was conducted at BGI Genomics (Shenzhen, China) using BGISEQ-500. We chose LV samples from model 6 and model 8, and the control was set as the sham group. Each group had five samples. LV tissues (25 mg) were immediately preserved in RNAlater (Thermo Fisher Scientific, Inc.). Total RNA extraction was performed using TRIzol (Thermo Fisher Scientific, Inc.), and RNA quantification, purity and integrity were evaluated using Fragment Analyzer 5200 (Agilent Technologies Co., Ltd.). Sequencing libraries were prepared according to the manufacturer's instructions using an MGIEasy RNA Library Prep Set (MGI Tech Co., Ltd.), with a sequencing length of 150 bp. Raw data underwent quality examination and cleansing using SOAPnuke (v1.5.6) (Li et al., 2008), followed by clean data alignment to the reference genome *Mus musculus* GCF_000001635.26_GRCm38.p6 using HISAT2 (v2.1.0) (Kim et al., 2015). The average uniquely mapping genome ratio was 81.31%. Table S13 shows the read count matrix of sequencing. Differential expression analysis was performed on read count matrices using DESeq2 (v1.4.5) (Love et al., 2014), to identify DEGs between control, MNx and adenine groups. DEGs with q-value<0.05 and log_2_|fold change|>1 were selected for further analysis. GO, KEGG and GSEA were conducted using Dr. Tom multi-omics data mining system (BGI Genomics Co., Ltd.).

### Immune infiltration analysis

The mMCP-counter (Petitprez et al., 2020) was employed to analyze the abundance of 16 cell types in LV tissues of MNx and adenine groups using RNA-sequencing data. The gene expression matrix was analyzed using R (version 4.1.3)-based mMCP-counter, and results were visualized using Prism 9 software (GraphPad Software, Inc.). Differential analysis of infiltrated cells was performed using multiple unpaired *t*-tests.

### PPI analysis of DEGs

DEGs from model 6 were used to construct an interaction network in the STRING database (confidence score>0.9) (Szklarczyk et al., 2011). Networks were visualized using Cytoscape (v 3.7.2) (Shannon et al., 2003), and subnetworks were identified using NetworkAnalyzer. Hub genes in the top three subnetworks were predicted using CytoHubba through the maximal clique centrality (MCC) algorithm.

### Western blotting

The total protein from LV tissues was extracted using RIPA lysis buffer (Beyotime Biotechnology Co., Ltd.; P0013B). Briefly, 20 mg of LV tissues were immersed in lysis buffer and homogenized with steel beads using TissueLyser-II (Qiagen, Inc.), and the supernatant was obtained for western blotting. An 8% SDS-PAGE gel was used for electrophoresis. Antibodies against P-p70S6K (Thr^389^/Thr^412^) (1:1000; Affinity Biosciences Co., Ltd.; AF3228), total p70S6K (1:2000; Proteintech Group, Inc.; 14485-1-AP) and HSP-90 (1:3000; Proteintech Group, Inc.; 13171-1-AP) served as primary antibodies, while IRDye^®^ 800CW Goat anti-Rabbit IgG Secondary Antibody (1:10000; LI-COR, Inc. 926-32211; ) served as the secondary antibody. Protein bands were visualized using an Odyssey Infrared Imaging System (LI-COR, Inc.), and relative expression levels were normalized to the HSP-90 band. Notably, incubation of P-p70S6K (Thr^389^/Thr^412^) antibodies was performed after the elution of total p70S6K antibodies. Images of uncropped gels are in Fig. S29.

### Flow cytometry analysis

Approximately 60 mg of LV tissues were placed into 1.5 ml tubes containing 500 μl 1640 medium and were cut into pieces with scissors. Tissue fragments were transferred into 15 ml tubes, and 0.5 μl/mg DNase I, 0.5 μl/mg collagenase II and Hanks’ balanced salt solution (HBSS) were added to a final volume of 3 ml. Tubes were incubated on a 37°C shaking table at 12 ***g*** for 40 min. The turbid liquid was passed through a 70 μm filter and centrifuged at 300 ***g*** for 5 min to remove the supernatant. Subsequently, 1 ml red blood cell lysis buffer was added and incubated for 3 min. Then, 6 ml 1640 medium was added, and centrifugation was performed at 300 ***g*** for 5 min to remove the supernatant. Finally, cells were resuspended in 200 μl HBSS buffer to obtain a single-cell suspension. After cell counting, 2×10^6^ cells were suspended in 100 μl HBSS buffer. Antibodies against F4/80 (1:100; BioLegend, Inc.; 123116), CD163 (1:100; Invitrogen, Inc.; 225-1631-82) and TIMD-4 (1:100; Invitrogen, Inc.; 12-5866-82) were added and incubated at room temperature for 30 min (protected from light). The cell suspension was then washed with HBSS buffer and centrifuged at 300 ***g*** for 5 min. Finally, cells were resuspended in 200 μl HBSS buffer for FACSCanto™ II flow cytometry analysis (BD Biosciences).

### Statistical analysis

Data are presented as mean±s.d. or median with interquartile range. The normal distribution of data was tested using the Shapiro–Wilk test. Differences between two groups were analyzed using a two-tailed *t*-test or Mann–Whitney *U*-test. One-way ANOVA or Kruskal–Wallis test was used for comparison between multiple groups, followed by Tukey's multiple comparisons test or Dunn's multiple comparisons test for multiple comparisons. Overall survival was evaluated using Kaplan–Meier survival analysis, followed by a Log-rank test for comparison between groups. Statistical analysis was performed using GraphPad Prism 9 software.

## Supplementary Material

10.1242/dmm.052395_sup1 Supplementary information

Table S13. Gene expression read counts.
