# Supplementary material for: C-C motif receptor 2 is a core profibrotic factor in uremic cardiomyopathy
Source: Dis Model Mech. 2026 Feb 3;19(1):dmm052395. doi: 10.1242/dmm.052395 (PMC12919955; doi:10.1242/dmm.052395)
Supplement: Supplementary information [file dmm-19-052395-s1.pdf]

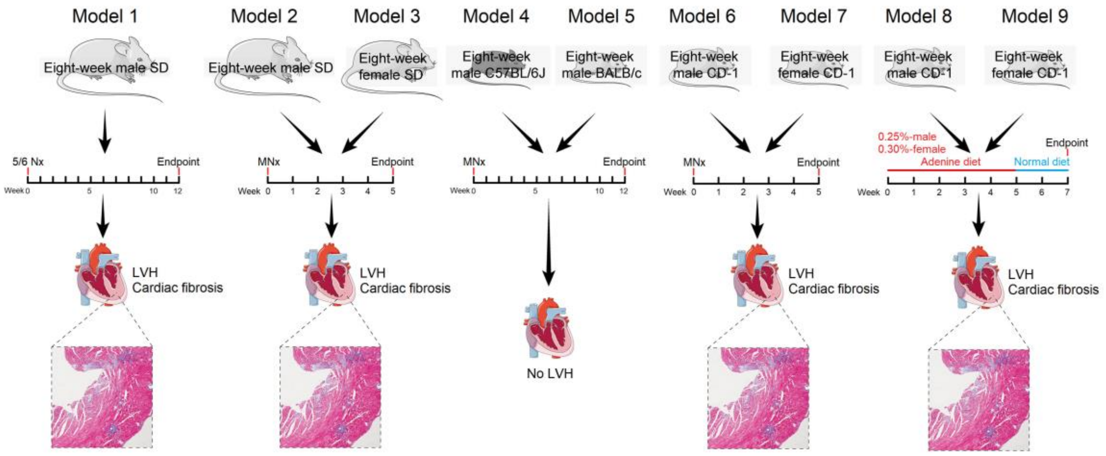

Result summary

	Renal function				Blood pressure		Cardiac parameters			
	Creatinie	Urea nitrogen	Serum phosphate	Renal pathology	Systolic	Diastolic	Realtive wall thcikness	Ejection fraction	Fibrosis	*Nppa-Nppb*
**Model 1**	↑3.1 fold	↑2.6 fold	↔	Interstitial fibrosis	/		↑1.3 fold	↔	↑2.9 fold	/
**Model 2**	↑7.0 fold	↑11.1 fold	↑1.7 fold	Interstitial fibrosis	↑1.6 fold	↑1.9 fold	↑1.4 fold	↔	↑3.0 fold	↑8.5 fold-↑5.5 fold
**Model 3**	↑4.7 fold	↑6.3 fold	↑1.5 fold	Interstitial fibrosis	↑1.4 fold	↑1.5 fold	↔	↔	↑2.7 fold	↑7.1 fold-↑1.9 fold
**Model 4**	↑1.8 fold	↑2.0 fold	↔	No significant fibrosis	↔		↔	↔	/	/
**Model 5**	↑2.4 fold	↑2.5 fold	↔	No significant fibrosis	↔	↑1.2 fold	↔	↔	/	/
**Model 6**	↑3.9 fold	↑5.0 fold	↑1.4 fold	Interstitial fibrosis	↑1.3 fold	↑1.5 fold	↑1.3 fold	↔	↑2.5 fold	↑12.8 fold-↑3.9 fold
**Model 7**	↑3.6 fold	↑4.7 fold	↑1.3 fold	Interstitial fibrosis	↑1.2 fold	↑1.3 fold	↑1.4 fold	↔	↑2.3 fold	↑6.3 fold-↑3.3 fold
**Model 8**	↑4.0 fold	↑5.3 fold	↑1.5 fold	Interstitial fibrosis	↑1.4 fold	↑1.4 fold	↑1.2 fold	↔	↑2.1 fold	↑2.6 fold-↑4.7 fold
**Model 9**	↑3.6 fold	↑4.8 fold	↑1.3 fold	Interstitial fibrosis	↑1.3 fold	↑1.2 fold	↑1.2 fold	↔	↑1.8 fold	↑2.8 fold-↑2.5 fold

↑: Increased ↓: Decreased ↔: Unchanged /: Not deteced

Fig. S1. Overview of UC models. CKD was induced in male and female SD, male C57BL/6J, male BALB/c, and male as well as female CD-1 by 5/6 Nx, MNx, or adenine diet. Among these rodents, male/female SD and CD-1 demonstrated UC phenotype, whereas male C57BL/6J and BALB/c had no significant UC phenotypes.

Disease Models & Mechanisms • Supplementary information

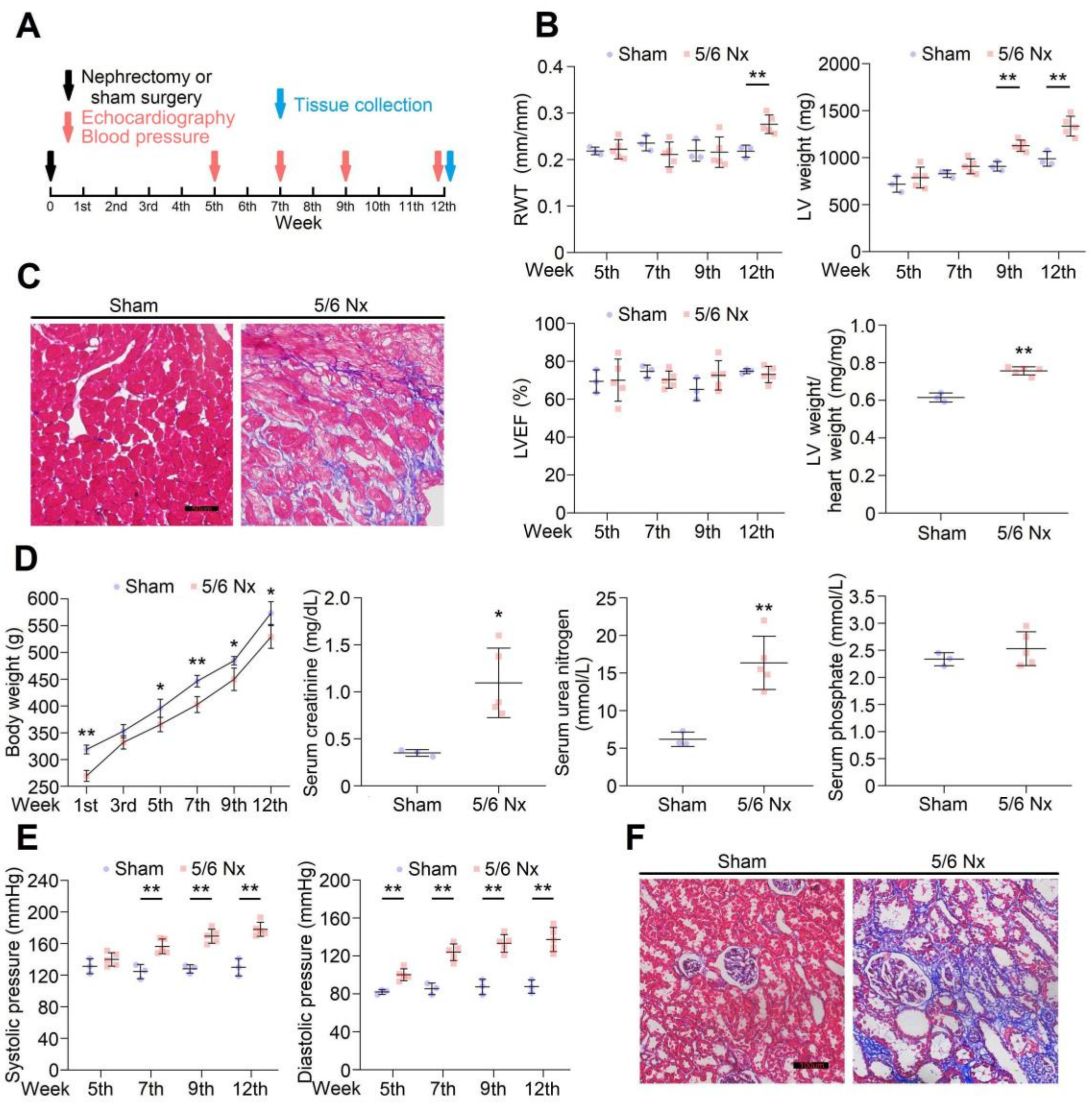

Fig. S2. Male SD rats develop UC in the 12th week after 5/6 Nx. (A) Observation contents after surgery. (B) LVH presented in nephrectomized rats in the 12th week after surgery. (C) Myocardial fibrosis can be observed in the 12th week after surgery (bar = 50 μm). (D) The body weight of rats in the CKD group was consistently lower than that in the sham group, and increased blood pressure, serum creatinine, serum urea nitrogen, and renal interstitial fibrosis, but not serum phosphate can be observed in these rats (bar = 100 μm). Sham, Sham group, n = 3; 5/6 Nx, 5/6 nephrectomy group, n = 5. Two-tailed t test was used. * P < 0.05, * P < 0.01.

Disease Models & Mechanisms • Supplementary information

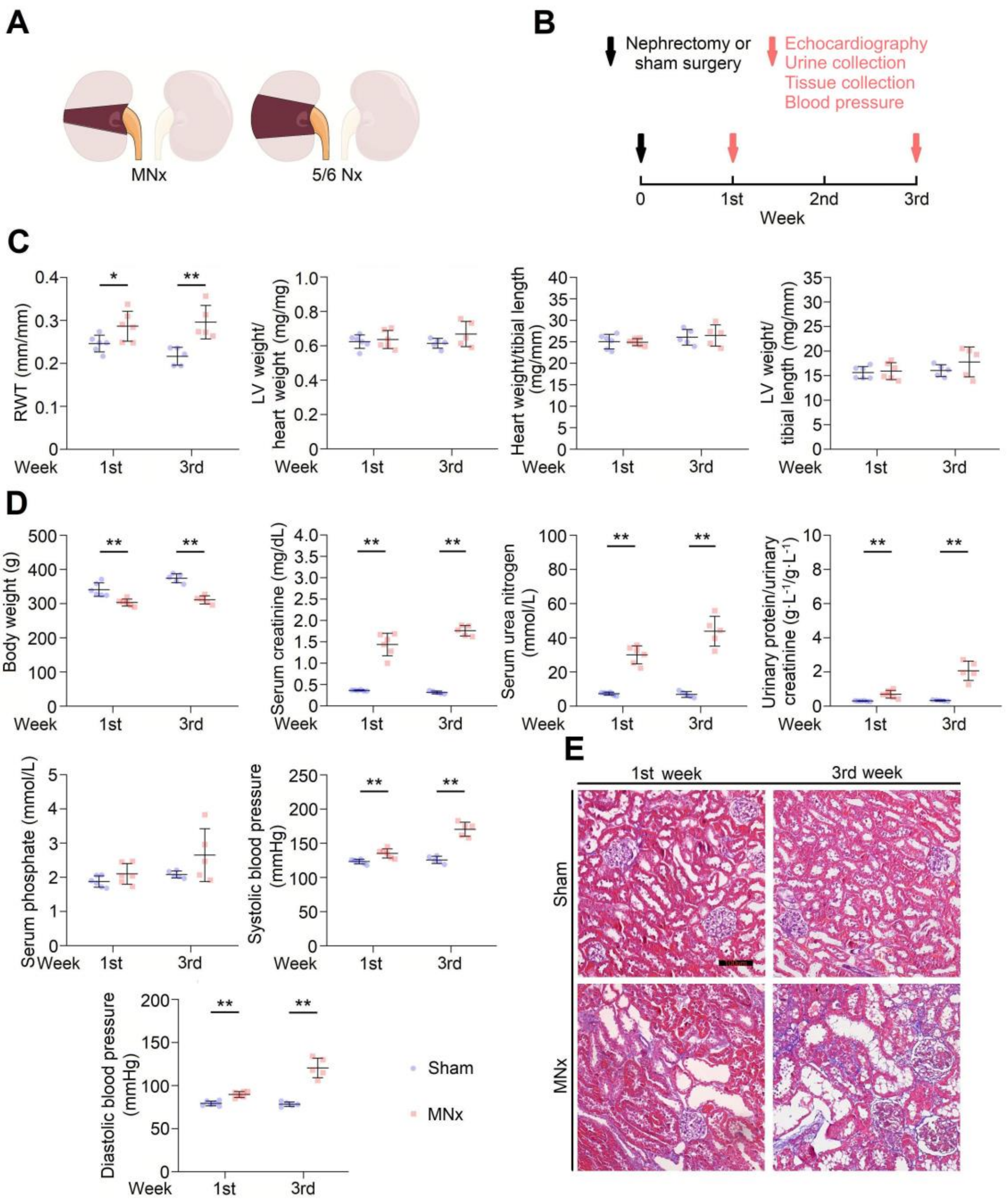

Fig. S3. MNx leads to rapid progression of LVH and renal injury in male SD rats. (A) Renal excision range of MNx and classical 5/6 Nx. (B) Observation contents after surgery. (C) Gradually increased LV wall thickness and LV weight can be observed in the 1st and 3rd week after MNx. (D) Nephrectomized rats had lower body weight, higher levels of serum creatinine, urea nitrogen, phosphate, urinary protein/creatinine, and blood pressures (E) Renal tubular dilatation and interstitial fibrosis were gradually observed in the nephrectomized rats (bar = 100 μm). Sham, Sham group, n = 5 or 6; MNx, Modified nephrectomy group, n = 5 or 6. Two-tailed t test was used. * $P < 0.05$, * $P < 0.01$.

Disease Models & Mechanisms • Supplementary information

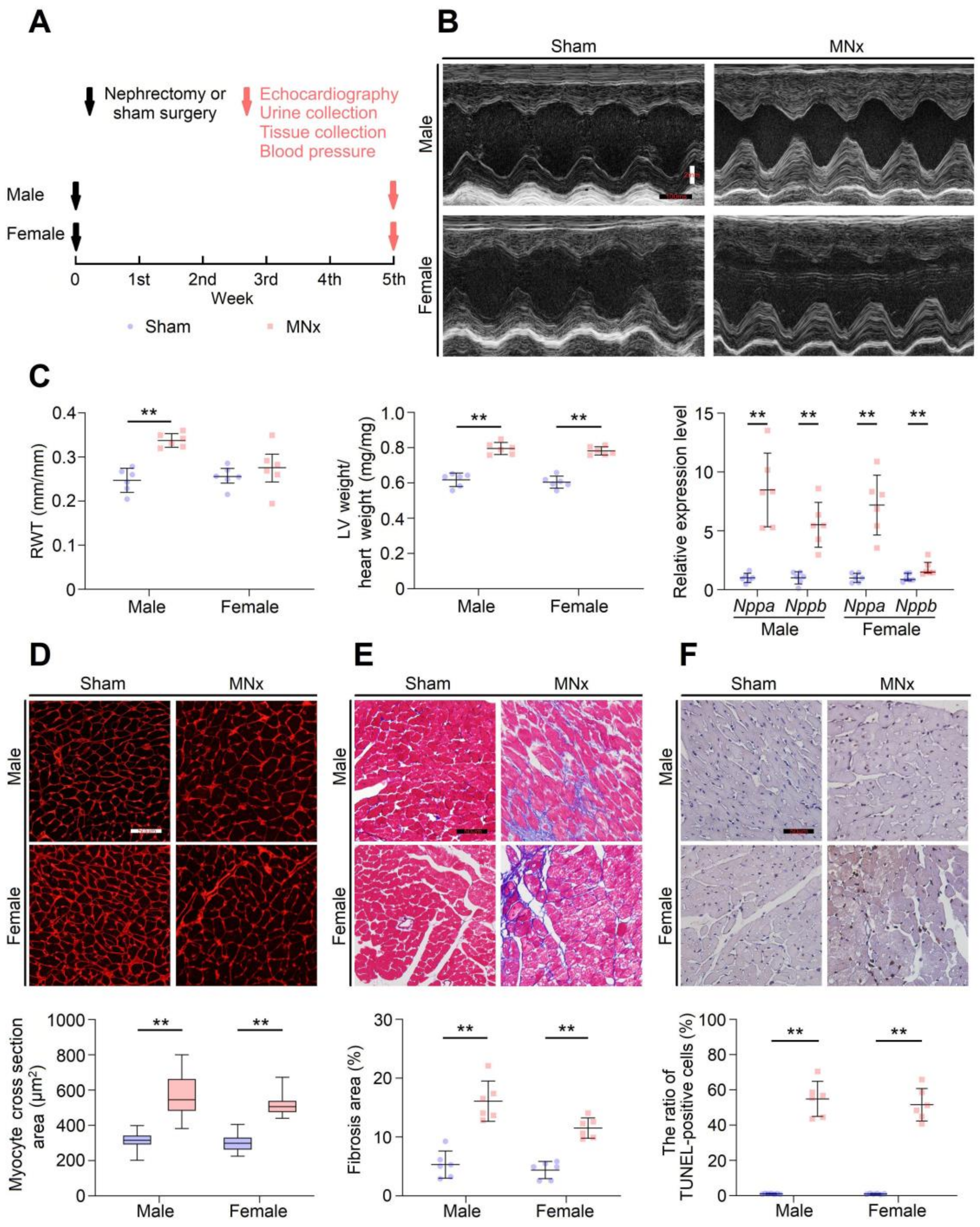

Fig. S4. MNx to UC in SD rats within 5 weeks. (A) Observation contents after surgery. (B) Representative M-mode diagram of echocardiography (black bar = 100 ms, white bar = 2 mm). (C) RWT, the ratio of LV weight/heart weight were, and LV expression of *Nppa* and *Nppb* were increased in the 5th week after surgery. (D) Enlarged cardiomyocytes can be observed in CKD group (bar = 50 μm, n = 360 cells per group). (E) Nephrectomized rats demonstrated significant cardiac fibrosis (bar = 50 μm). (F) Cardiomyocyte apoptosis is increased in rats with UC (bar = 50 μm). Two-tailed t test or Mann-Whitney U test was used. Sham, Sham group, n = 6; MNx, Modified nephrectomy group, n = 6. ** P < 0.01.

Disease Models & Mechanisms • Supplementary information

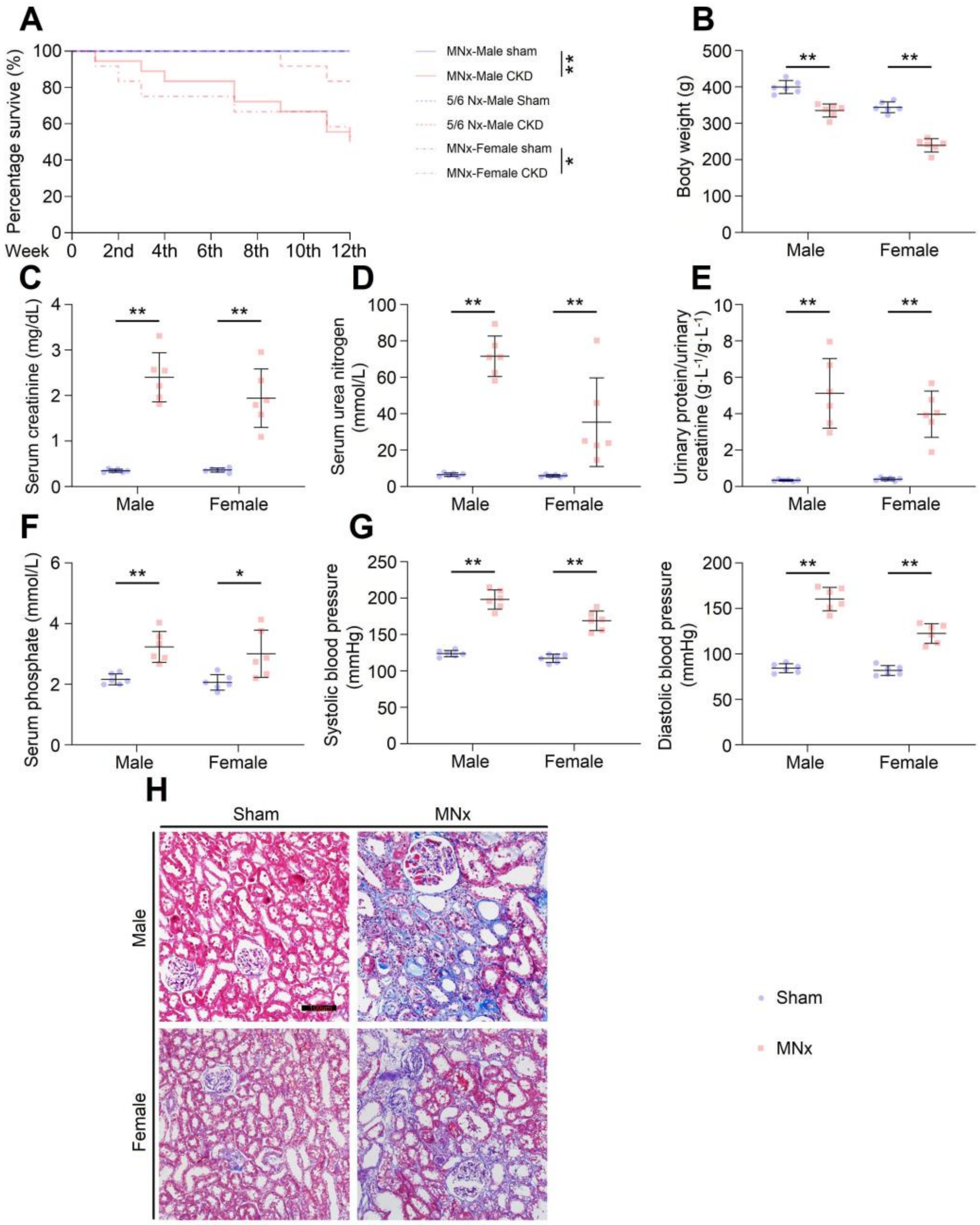

Fig. S5. Serious impairment of renal function in SD rats with MNx. (A) Mortality of male rats with 5/6 Nx and MNx, and female rats with MNx (MNx-Male sham = 12, MNx-Male CKD = 18, 5/6 Nx-Male sham = 8, 5/6 Nx-Male CKD = 12, MNx-female sham = 8, MNx-female CKD = 12). (B) Nephrectomized rats had lower body weight than sham rats. (C-F) The levels of serum creatinine, urea nitrogen, phosphate and urinary protein/creatinine in surgery group were significantly increased, and females had mild renal impairment compared to males. (G) Blood pressures of nephrectomized rats were increased. (H) Renal tubular dilatation and interstitial fibrosis were gradually observed in the nephrectomized rats (bar = 100 μm). Sham, Sham group, n = 6; MNx, Modified nephrectomy group, n = 6. Two-tailed t test or Log-rank test was used. * $P < 0.05$, * $P < 0.01$.

Disease Models & Mechanisms • Supplementary information

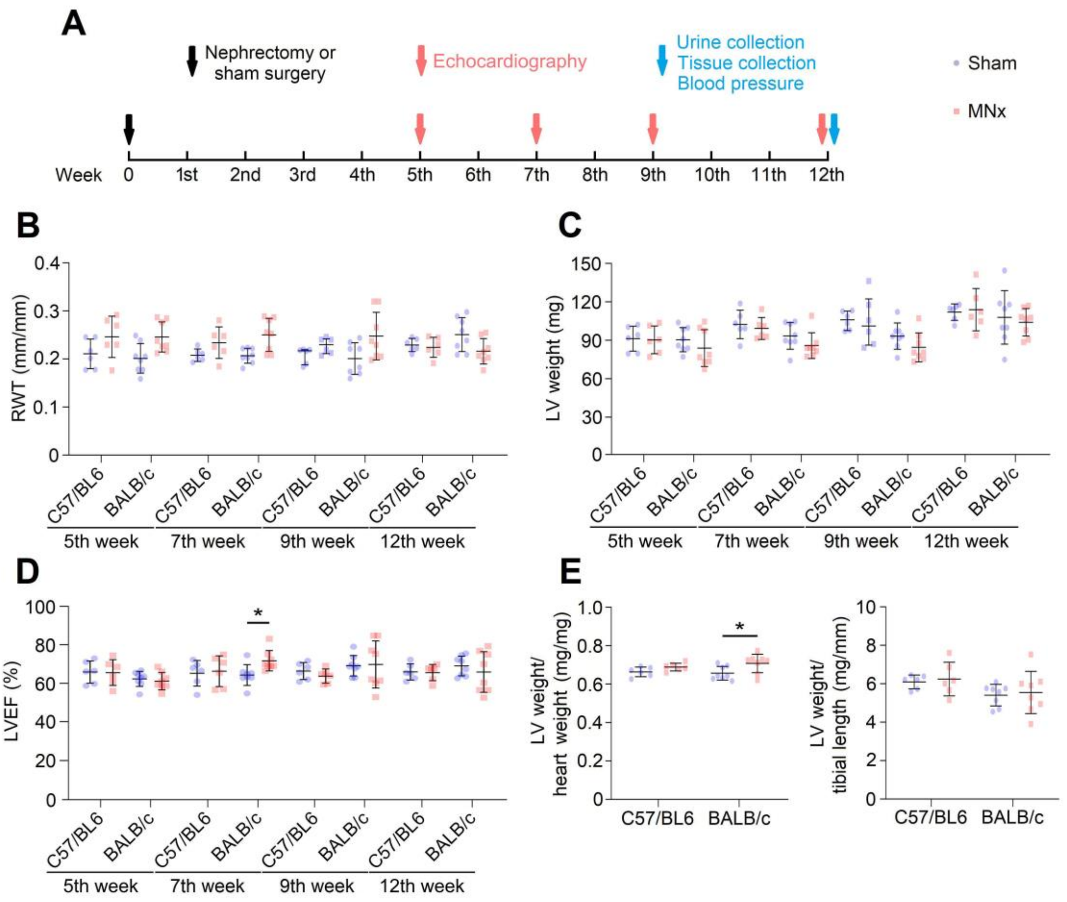

Fig. S6. MNx fails to induce UC in C57BL/6J and BALB/c mice within 12 weeks. (A) Observation contents after surgery. (B-C) There was no significant differences on RWT, and LV weight between the sham and the CKD groups until the 12th week after nephrectomy. (D) Unchanged LVEF can be observed in the surgery group until the 12th week after nephrectomy. (E) 12 weeks after MNx, LV weight/heart weight and LV weight/tibial length of C57/BL6 and BALB/c mice in the surgery group had no significant differences with those in the sham operation group. Sham, Sham group, n = 6 or 8; MNx, Modified nephrectomy group, n = 6 or 8. Two-tailed t test was used. * $P < 0.05$.

Disease Models & Mechanisms • Supplementary information

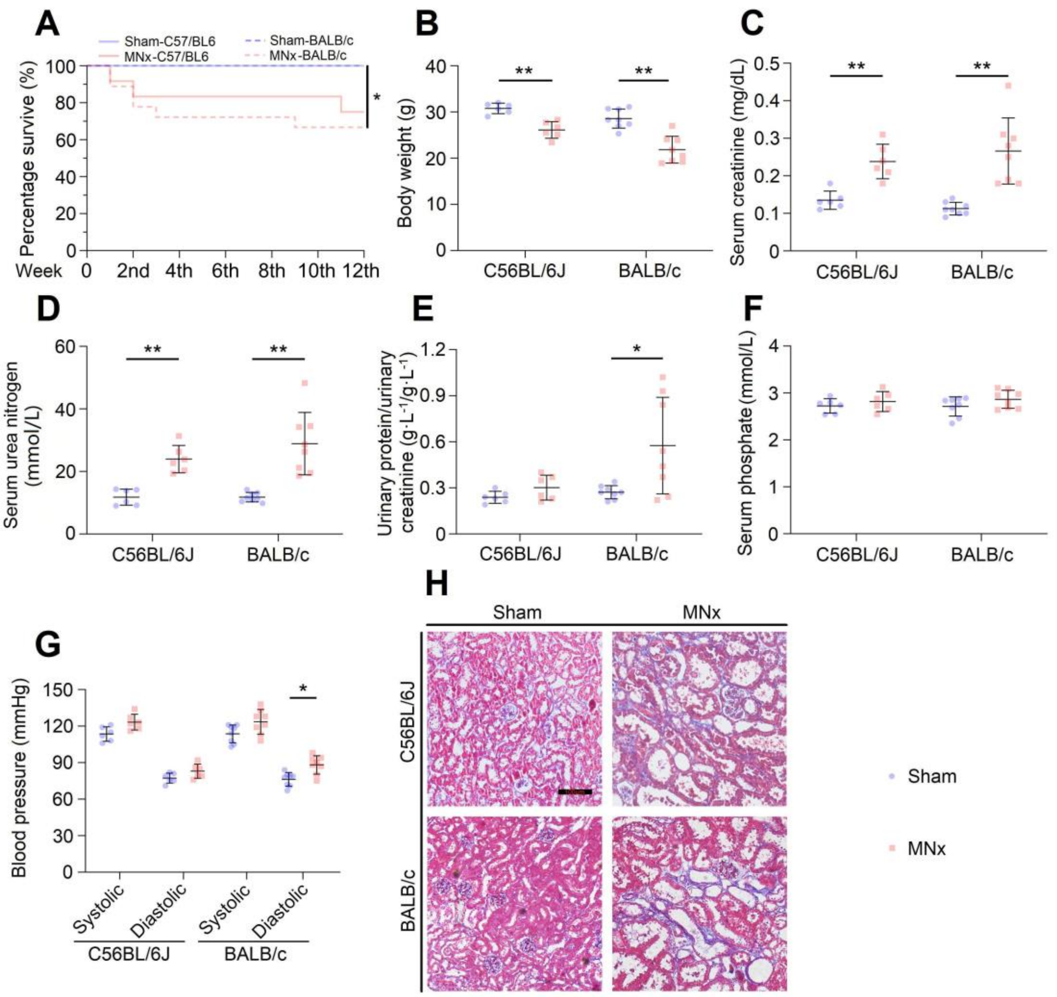

Fig. S7. MNx leads to mild renal impairment in C57BL/6J and BALB/c mice in the 12th week after surgery. (A) There was a significant difference in mortality between the sham and the CKD groups of BALB/c mice (n = 8 in sham, n = 12 in CKD), but not C57/BL6 mice (n = 12 in sham, n = 18 in CKD). (B) The body weights of nephrectomized mice were lower than sham group. (C-E) The levels of serum creatinine, urea nitrogen and urinary protein/creatine (except C57BL/6J mice) in the surgery group were mildly increased. (F) The serum phosphate level in the surgery group was unchanged. (G) Mild diastolic blood pressure increase can be observed in nephrectomized BALB/c mice. (H) Residual renal tissue of nephrectomized mice showed renal tubular dilation and glomerular hypertrophy, while interstitial fibrosis was not obvious (bar = 100 µm). Sham, Sham group, n = 6 or 8; MNx, Modified nephrectomy group, n = 6 or 8. Two-tailed t test or Log-rank test was used. * $P < 0.05$, ** $P < 0.01$.

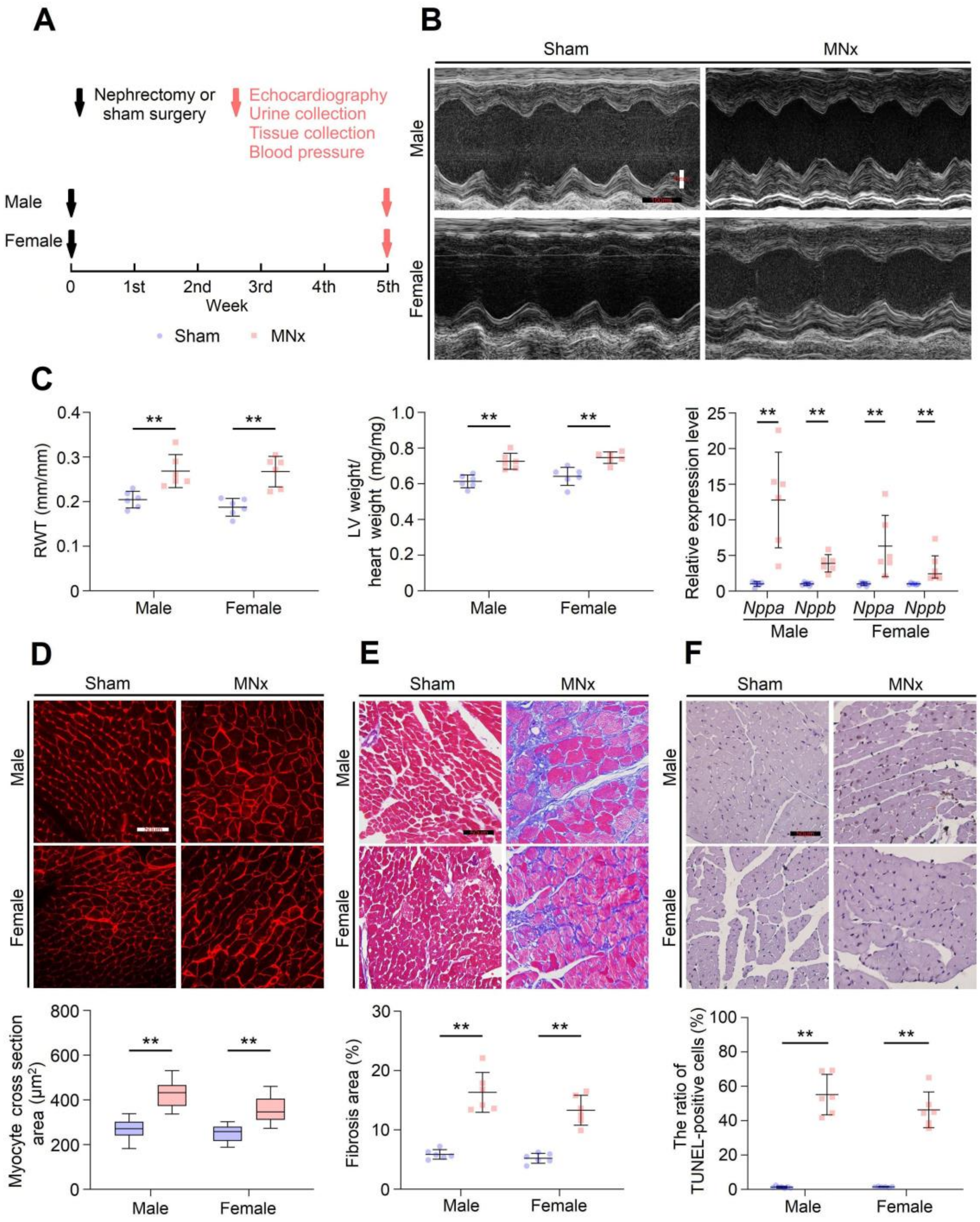

Fig. S8. Male and female CD-1 develop UC in the 5th week after MNx. (A) Schedule of MNx. (B) Representative M-mode diagram of echocardiography (black bar = 100 ms, red bar = 1 mm). (C) RWT, LV weight/heart weight, and the LV expression of *Nppa*/*Nppb* were significantly increased in surgery group. (D-F) Nephrectomized mice had enlarged cardiomyocytes (bar = 50 µm, n = 360 cells per group), LV fibrosis (bar = 50 µm), and increased cardiomyocyte apoptosis (bar = 50 µm). Sham, Sham group, n = 6; MNx, Modified nephrectomy group, n = 6. Two-tailed t test or Mann-Whitney U test was used. ** $P < 0.01$.

Disease Models & Mechanisms • Supplementary information

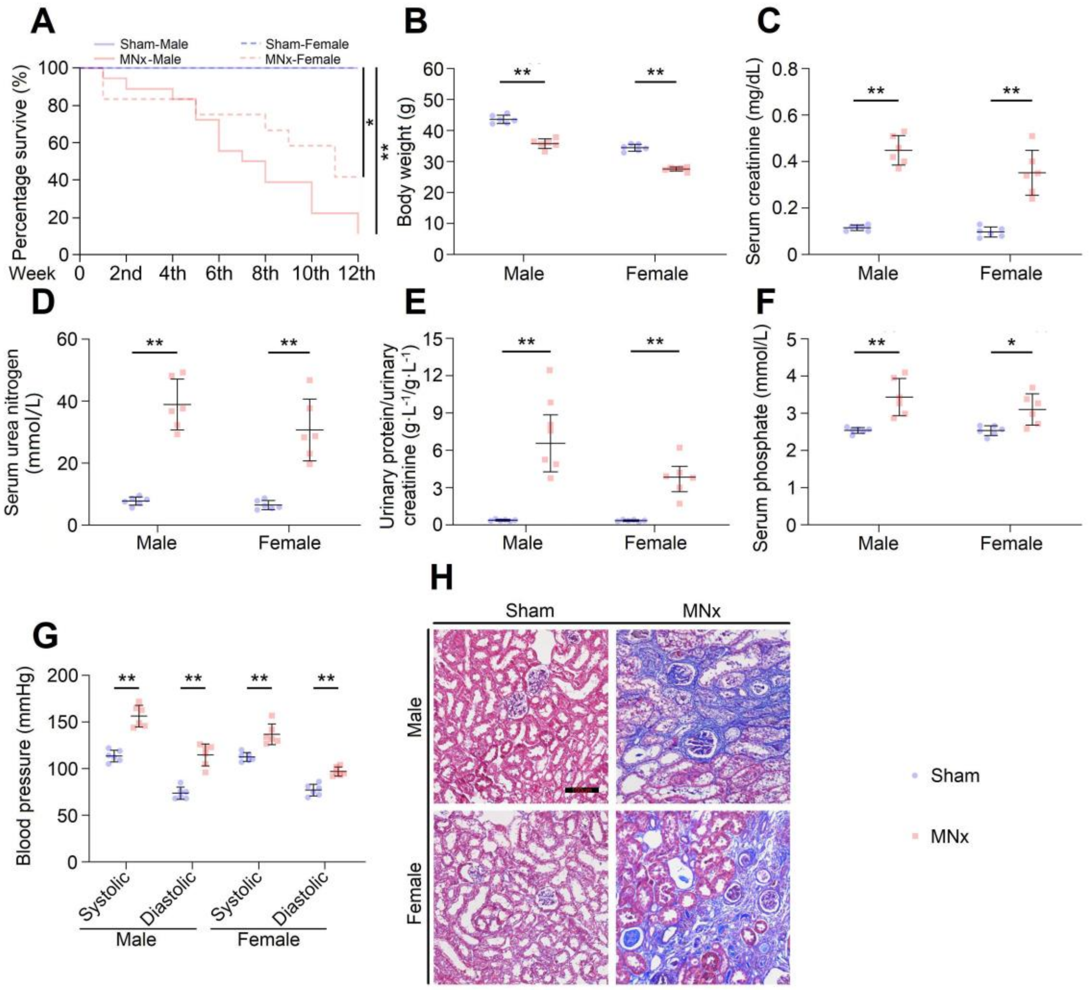

Fig. S9. MNx results in renal impairment in CD-1 mice in the 5th week after surgery. (A) The mortality of nephrectomized mice was higher (male: n = 12 in sham, n = 18 in CKD; female: n= 8 in sham, n = 12 in CKD). (B) The body weights of nephrectomized mice were lower than sham mice. (C-F) Serum creatinine, urea nitrogen, phosphate, and urinary protein/creatinine levels of nephrectomized mice were significantly increased. (G) Hypertension can be observed in nephrectomized mice. (H) Renal tubular dilatation, glomerular hypertrophy and interstitial fibrosis were observed in the residual renal tissue of nephrectomized mice (bar = 100 μm). Sham, Sham group, n = 6; MNx, Modified nephrectomy group, n = 6. Two-tailed t test or Mann-Whitney U test or Log-rank test was used. * $P < 0.05$, ** $P < 0.01$.

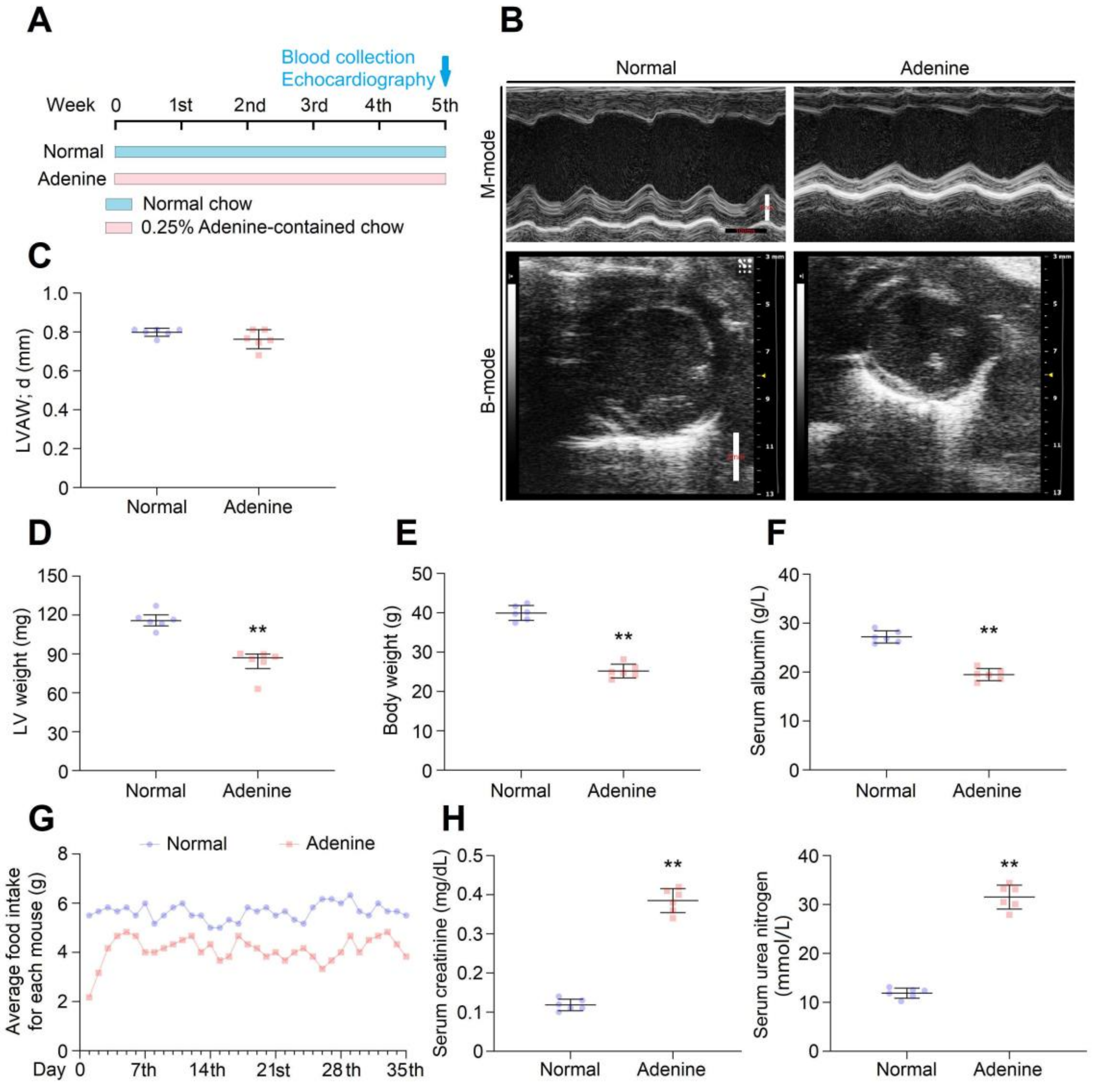

Fig. S10. 5-week 0.25% adenine diet leads to prominent malnutrition and smaller heart. (A) Feeding plan and observation contents. (B) Representative M-mode and B-mode diagrams of echocardiography after 5-week adenine diet (M-mode: black bar = 100 ms, white bar = 1 mm; B-mode: 2 mm). (C) There was no significant difference in LV wall thickness between the adenine and the control group. (D) LV weight of adenine diet-feeding mice was significantly lower than normal mice. (E) 5-week adenine diet causes significant weight loss. (F) Serum albumin in adenine diet-feeding mice was overtly lower. (G) The average food intake of adenine diet-feeding mice was lower than that of the normal diet-feeding mice. (H) Adenine group exhibited increased serum creatinine and urea nitrogen. Normal, Normal diet group, n = 6; Adenine, 0.25% Adenine diet group, n = 6. Two-tailed t test was used. ** $P < 0.01$.

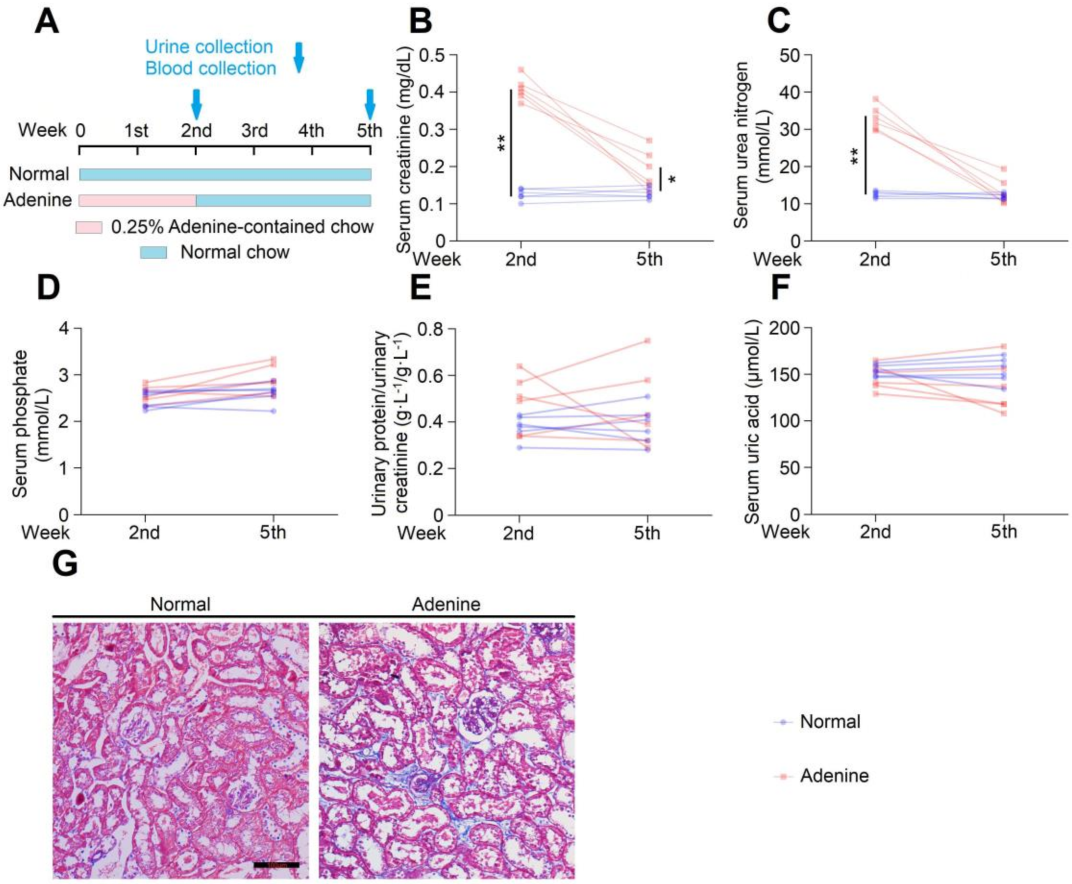

Fig. S11. 2-week 0.25% adenine diet results in reversible renal impairment in male CD-1 mice. (A) Feeding plan and observation contents. (B-C) Serum creatinine and urea nitrogen in mice fed with adenine diet decreased almost to normal level after 3-week normal diet feeding. (D-F) There were no significant changes in serum phosphate, uric acid and urinary protein/creatinine levels of adenine diet-feeding mice. (G) Only slight tubular dilation, interstitial fibrosis occurred in mice fed with 2-week adenine diet (bar = 100 μm). Normal, Normal diet group, n = 6; Adenine, 0.25% Adenine diet group, n = 6. Two-tailed t test was used. * $P < 0.05$, ** $P < 0.01$.

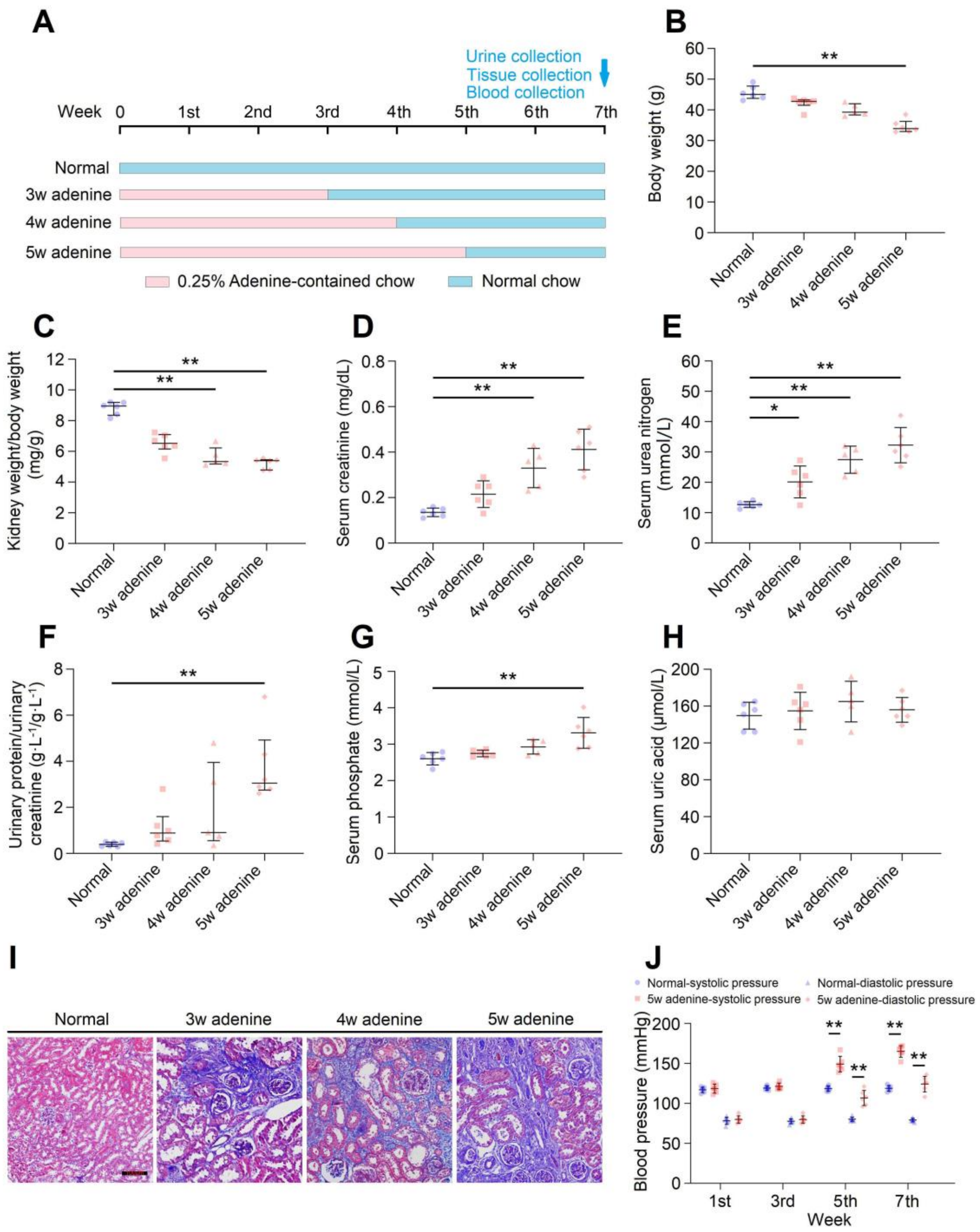

Fig. S12. 5-week 0.25% adenine diet causes irreversible renal impairment in male CD-1 mice. (A) Feeding plan and observation contents. (B-C) 5-week 0.25% adenine diet led to significant weight loss and renal atrophy. (D-G) The levels of serum creatinine, urea nitrogen, phosphate and urinary protein/creatinine in 5-week 0.25% adenine diet-feeding mice were significantly increased. (H) There was no significant difference in serum uric acid level among all groups. (I) Glomerulosclerosis and interstitial fibrosis occurred in renal tissues of 0.25% adenine diet-feeding mice, and the longer the feeding time, the more obvious the fibrosis was

Disease Models & Mechanisms • Supplementary information

(bar = 100 µm). (J) Hypertension can be found in male CD-1 mice with 5-week 0.25% adenine diet. Normal, Normal diet group, n = 6; Adenine, 0.25% Adenine diet group, n = 6. One-way ANOVA test followed by Tukey's multiple comparisons test or Kruskal-Wallis test followed by Dunn's multiple comparisons test or two-tailed t test was used. * $P < 0.05$, ** $P < 0.01$.

Disease Models & Mechanisms • Supplementary information

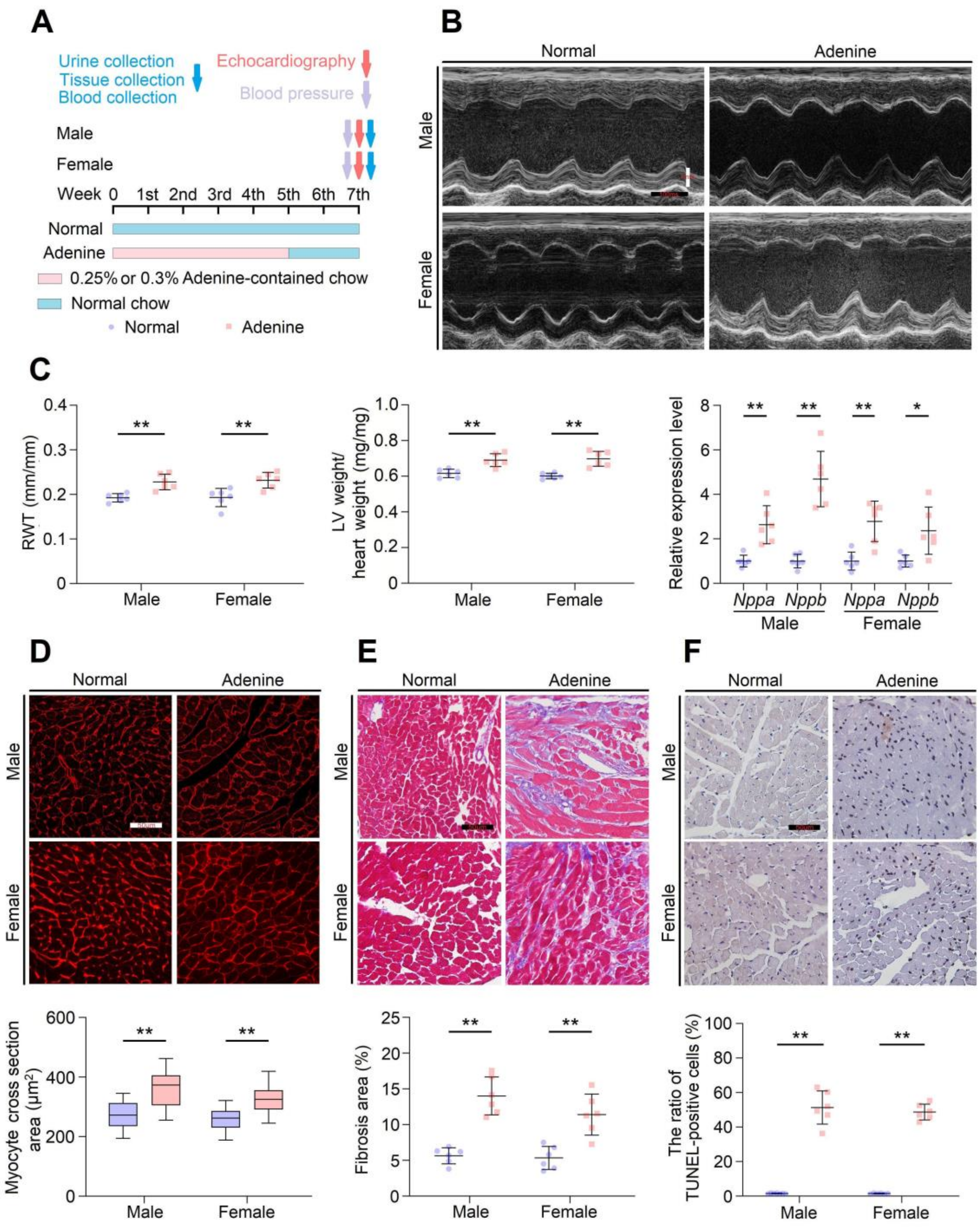

Fig. S13. UC appears in male and female CD-1 mice fed with 0.25% adenine and 0.3% adenine diet respectively. (A) Feeding schedule. (B) Representative M-mode diagram of echocardiography (black bar = 100 ms, red bar = 1 mm). (C) RWT, LV weight/heart weight, and the LV expression of *Nppa*/*Nppb* were significantly higher. (D-F) Mice with adenine diet had enlarged cardiomyocytes (bar = 50 μm, n = 360 cells per group), LV fibrosis (bar = 50 μm), and increased cardiomyocyte apoptosis (bar = 50 μm). Normal, Normal diet group, n = 6; Adenine, 0.25% Adenine diet group (male) or 0.3% Adenine diet group (female), n = 6. Two-tailed t test or Mann-Whitney U test was used. * $P < 0.05$, ** $P < 0.01$.

Disease Models & Mechanisms • Supplementary information

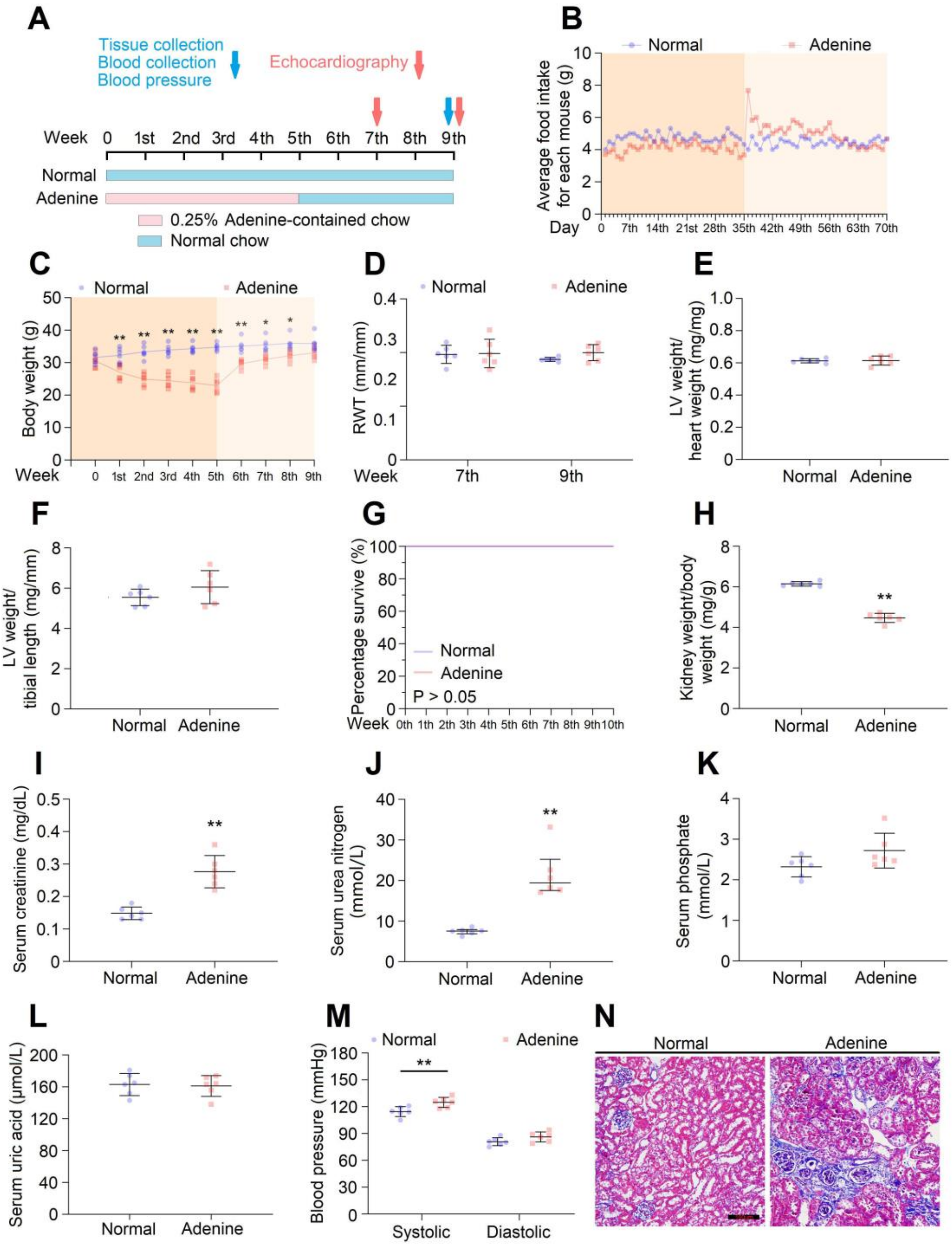

Fig. S14. 5-week 0.25% adenine diet and 4-week normal diet cause mild renal injury without UC phenotype in female CD-1 mice. (A) Feeding plan and observation contents. (B) Changing to normal diet significantly increased the average food intake. (C) The body weights of adenine diet-feeding mice increased significantly when fed with normal diet, and there was no significant difference in body weight between the two groups in the 9th week. (D-F) RWT, LV weight/heart weight, and LV weight/tibial length were significantly higher in adenine group in the

Disease Models & Mechanisms • Supplementary information

9th week; however, there were some of female mice had no significant LVH. (G) No death was observed in adenine group (n = 8 in control, n =12 in adenine). (H-J) Adenine group demonstrated renal atrophy, increased serum creatinine and urea nitrogen in the 9th week. (K) There was no significant hyperphosphatemia in adenine group. (L) There was no significant difference in serum uric acid between the adenine group and the control group. (M) Blood pressures of adenine diet-feeding mice increased slightly. (N) Renal tubular hypertrophy and interstitial fibrosis were obvious in adenine diet feeding mice (bar = 100 μm). Normal, Normal diet group, n = 6; Adenine, 0.25% Adenine diet group, n = 6. Two-tailed t test or Mann-Whitney U test or Log-rank test was used. * $P < 0.05$, ** $P < 0.01$.

Disease Models & Mechanisms • Supplementary information

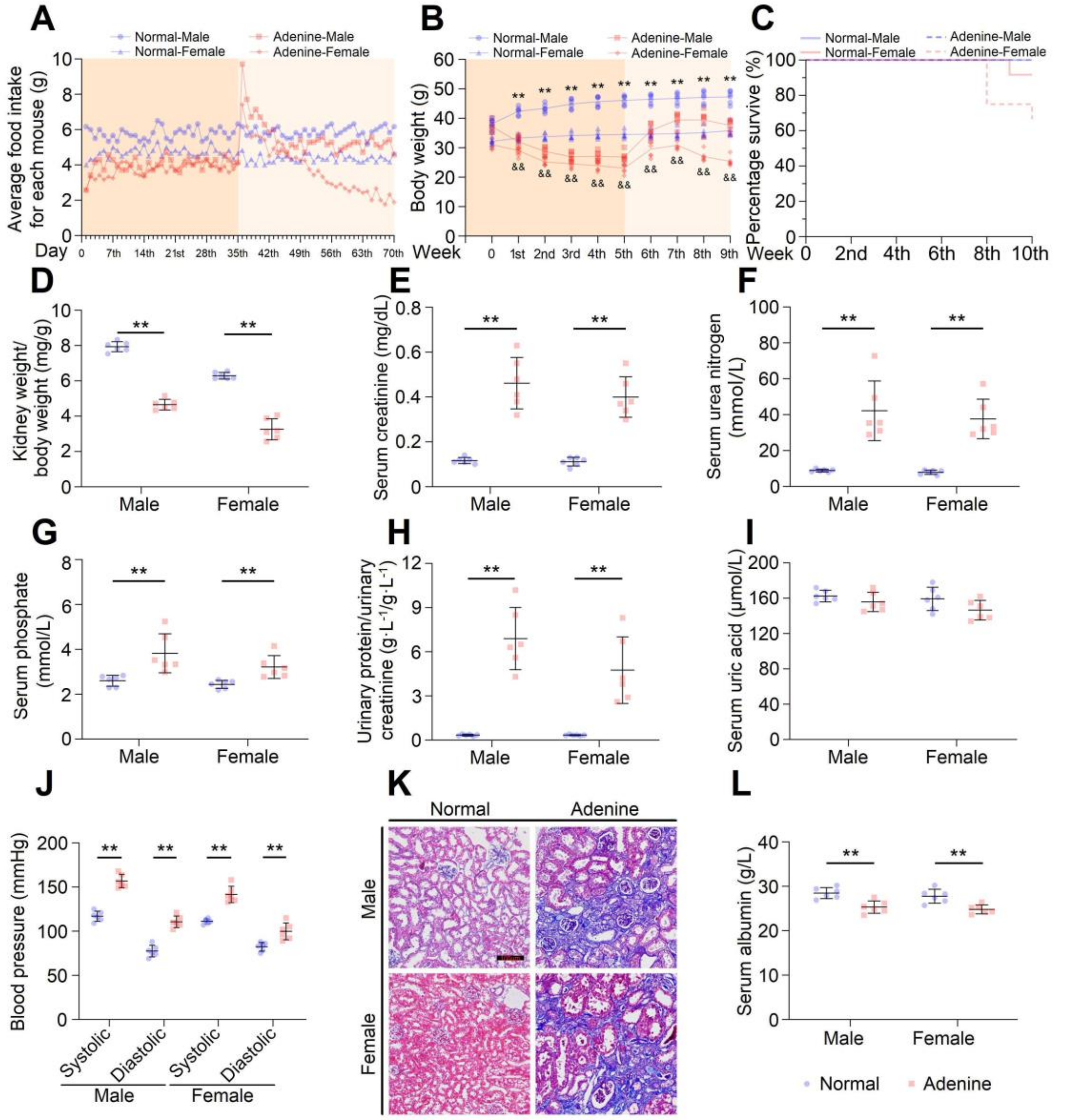

Fig. S15. The combination of adenine diet and normal diet results in renal impairment in CD-1 mice. (A-B) Changing to normal diet significantly increased body weight and average food intake in adenine group. (C) The mortality of adenine diet-feeding mice (male: n= 8 in control, n = 12 in adenine; female: n= 8 in control, n = 12 in adenine). (D) Adenine group demonstrated renal atrophy. (E-H) The levels of serum creatinine, urea nitrogen, phosphate, and urinary protein/creatinine were significantly higher in adenine group. (I) There was no difference in serum uric acid between the adenine and control groups. (J) Hypertension can be observed in adenine group. (K) Renal tubular hypertrophy and interstitial fibrosis were obvious in mice

fed with adenine diet (bar = 100 μm). (L) Serum albumin in adenine group was slightly lower than that in the control group. Normal, Normal diet group, n = 6; Adenine, 0.25% Adenine diet group (male) or 0.3% Adenine diet (female), n = 6. Two-tailed t test or Log-rank test was used. * Control-Male with Adenine-Male, $^{\&}$ Control-Female with Adenine-Female. $^{\&\&}$ ** $P < 0.01$.

Disease Models & Mechanisms • Supplementary information

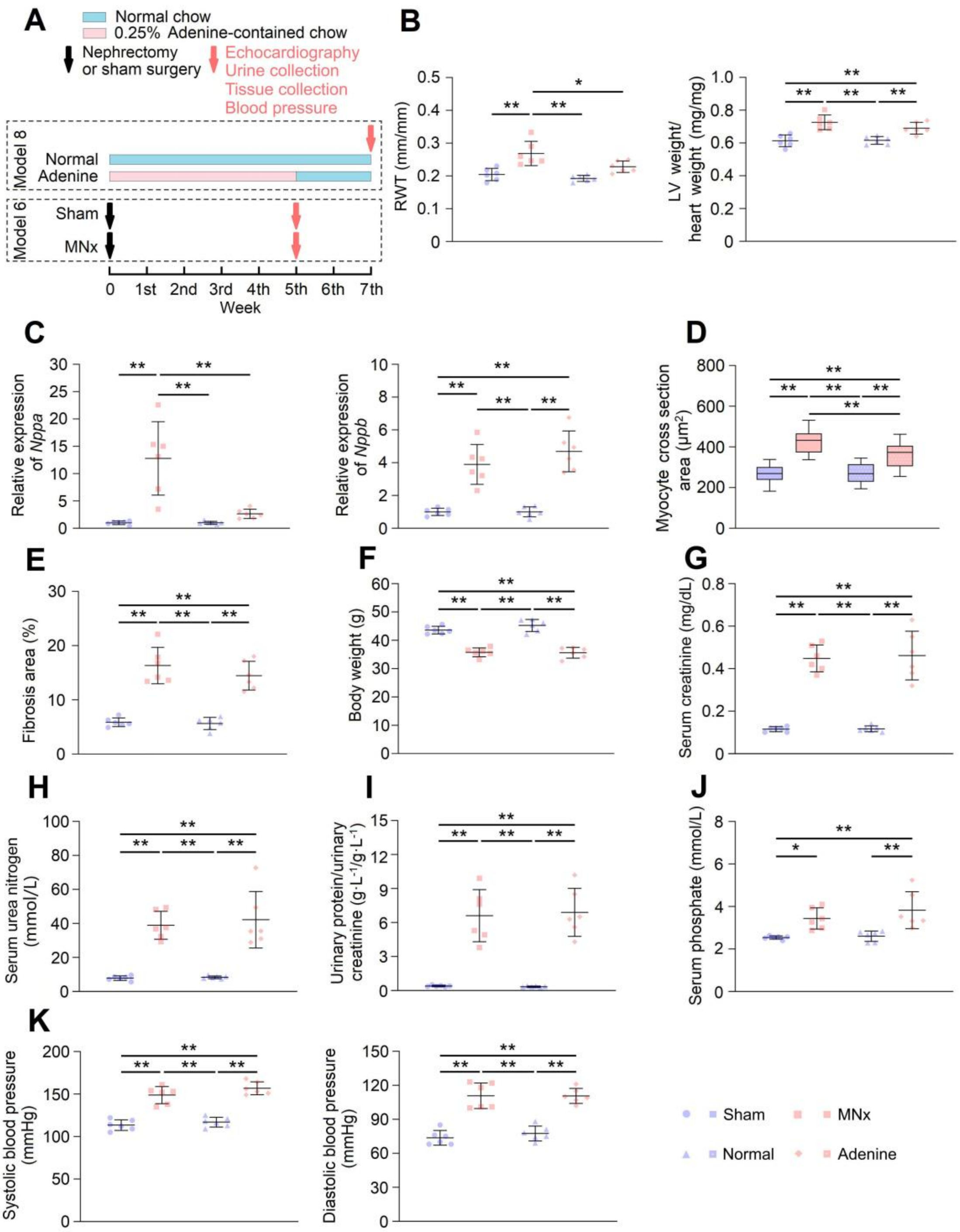

Fig. S16. Male CD-1 with adenine have slighter phenotypes of UC compared to male CD-1 with MNx. (A) Schedule of MNx and adenine diet. (B-C) RWT, LV weight/heart weight, and the LV expression of *Nppa*/*Nppb* were increased in mice with MNx and adenine, but mice with adenine had milder changes. (D-E) Mice with MNx and adenine had enlarged cardiomyocytes and LV fibrosis, but mice with

Disease Models & Mechanisms • Supplementary information

adenine had smaller cardiomyocytes than mice with MNx (n = 360 cells per group). (F) Mice with MNx and adenine demonstrated lower body weight. (G-J) Increased serum creatinine, urea nitrogen, urinary protein/creatinine, and phosphate were similar in mice with MNx and adenine. (K) Hypertension was observed in mice with MNx and adenine. Sham, Sham group, n = 6; MNx, Modified nephrectomy group, n = 6; Normal, Normal diet group, n = 6; Adenine, 0.25% Adenine diet group, n = 6. One-way ANOVA test followed by Tukey's multiple comparisons test was used. * $P < 0.05$, ** $P < 0.01$.

Disease Models & Mechanisms • Supplementary information

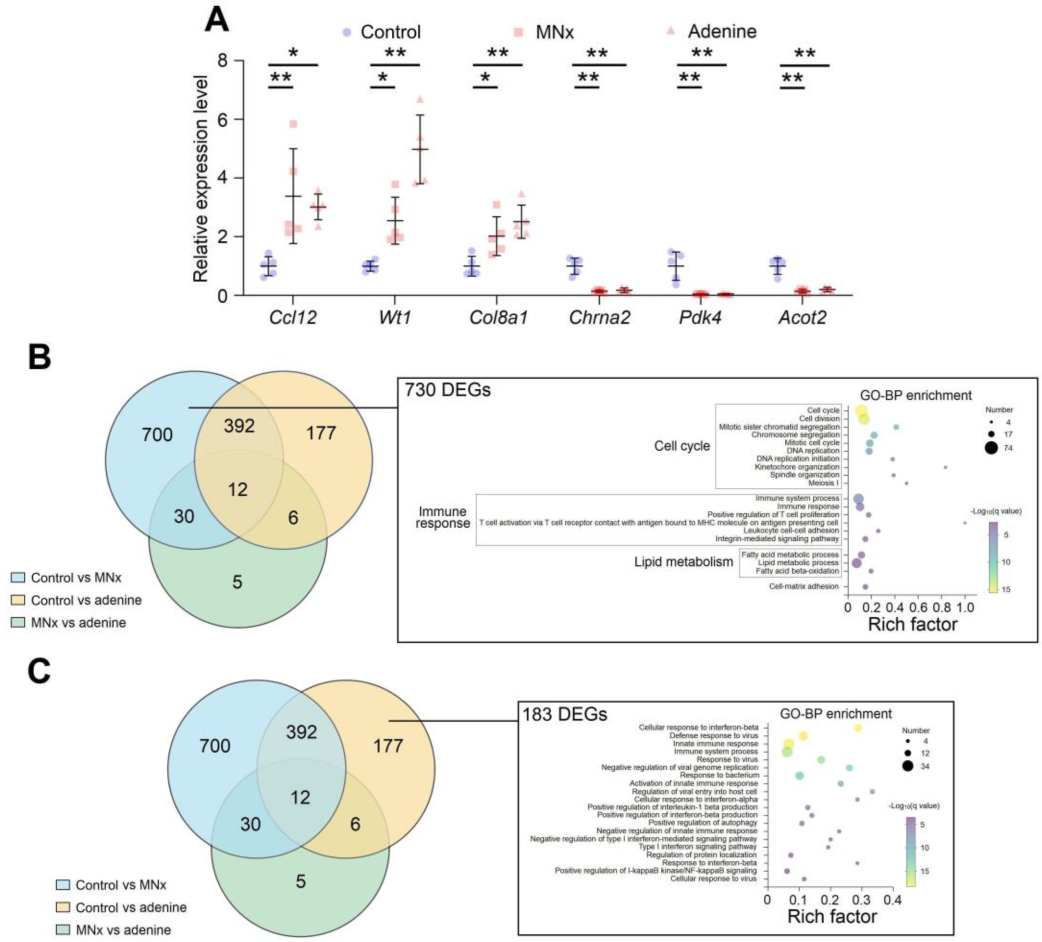

Fig. S17. Unique DEGs in MNx- and adenine-induced UC. (A) The confirmation of RNA sequencing data through qPCR. (B) The GO-biological process enrichment of unique DEGs in MNx-induced UC. (C) The GO- biological process enrichment of unique DEGs in adenine-induced UC. Control, Control group, n = 5; MNx, Modified nephrectomy group, n = 5; Adenine, 0.25% Adenine diet group, n = 5. One-way ANOVA test followed by Tukey's multiple comparisons test was used. * P < 0.05, ** P < 0.01.

Disease Models & Mechanisms • Supplementary information

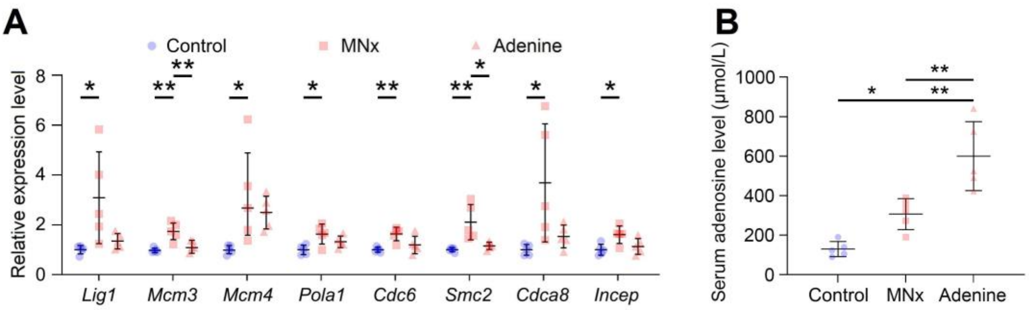

Fig. S18. Alternations of cell cycle and serum adenosine level in UC. (A) qPCR confirmed the upregulated genes related to cell cycle in UC. (B) Adenine-induced UC had a higher serum adenosine level compared to MNx-induced UC and control. Control, Control group, n = 5; MNx, Modified nephrectomy group, n = 5; Adenine, 0.25% Adenine diet group, n = 5. One-way ANOVA test followed by Tukey's multiple comparisons test was used. * $P < 0.05$, ** $P < 0.01$.

Disease Models & Mechanisms • Supplementary information

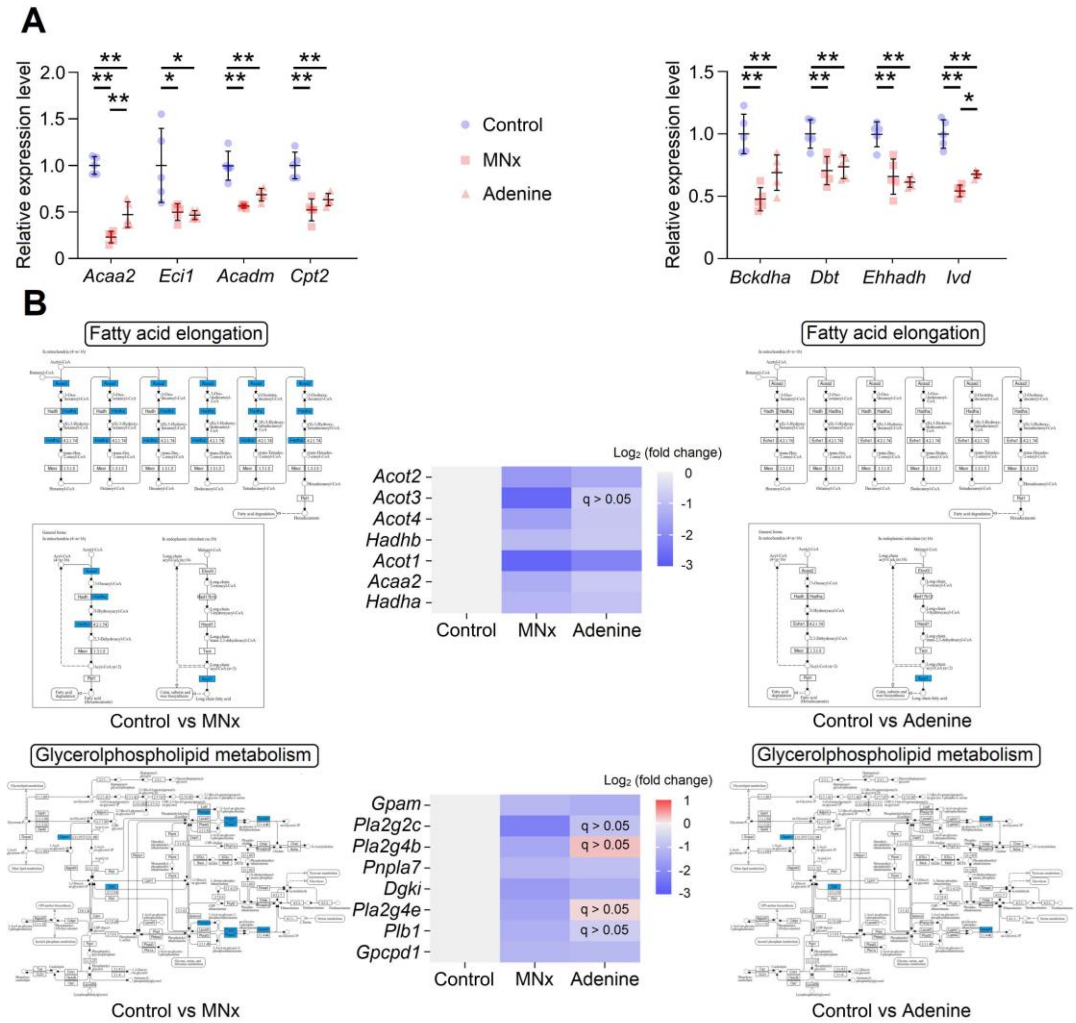

Fig. S19. Alternations of FA metabolism in UC. (A) qPCR confirmed the downregulated genes related to FA degradation and BCAA degradation in UC. (B) Adenine-induced UC had minor changes compared to MNx in FA elongation and glycerophospholipid metabolism. Control, Control group, n = 5; MNx, Modified nephrectomy group, n = 5; Adenine, 0.25% Adenine diet group, n = 5. One-way ANOVA test followed by Tukey's multiple comparisons test was used. * P < 0.05, ** P < 0.01.

Disease Models & Mechanisms • Supplementary information

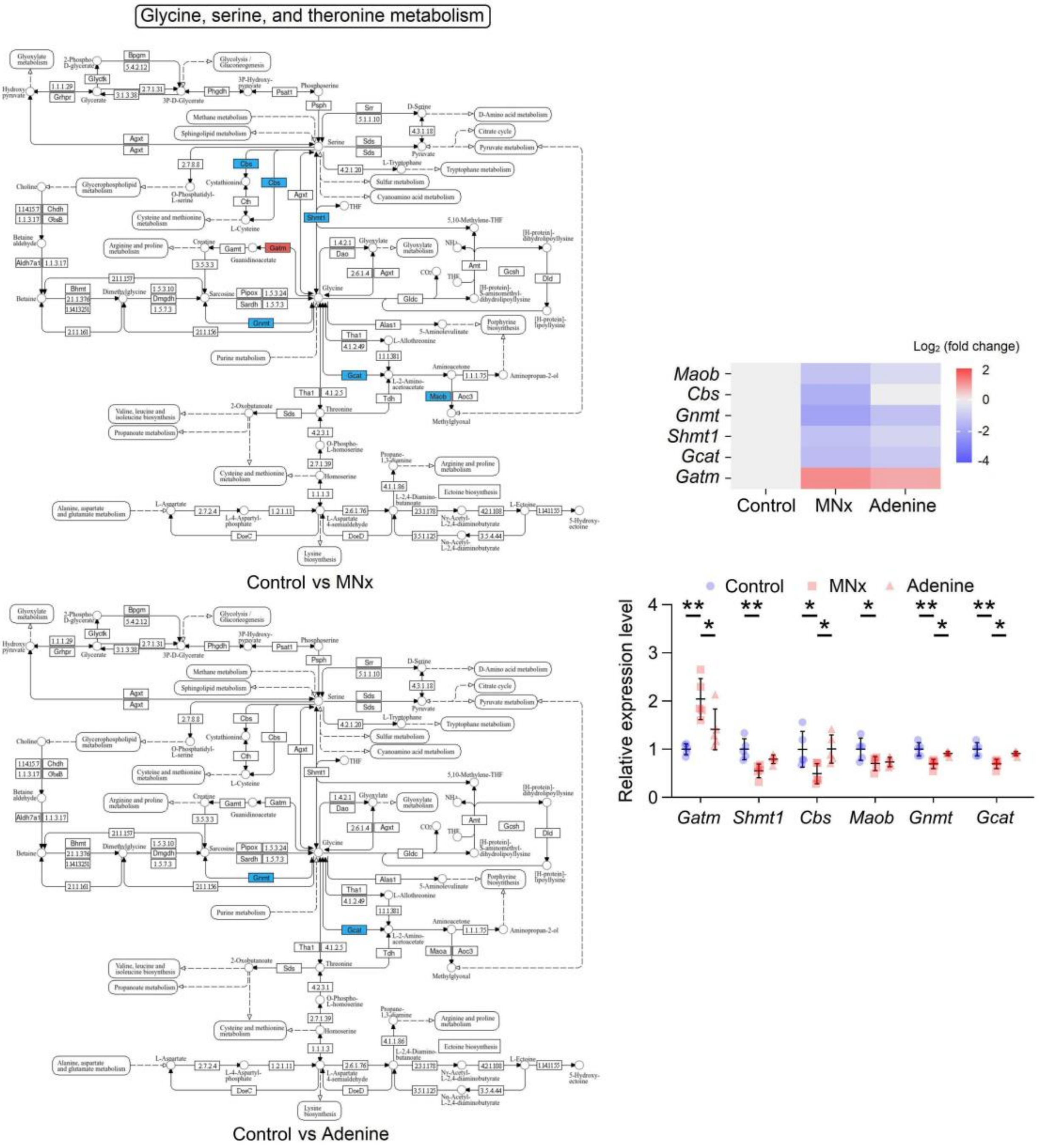

Fig. S20. Alternations of AA metabolism in UC. Both MNx- and adenine-induced UC demonstrated alternations of glycine, serine, threonine, tyrosine, and tryptophan metabolism, while adenine-induced UC had minor changes compared to MNx. qPCR analysis also showed less change of related metabolic enzymes in adenine-induced UC. Control, Control group, n = 5; MNx, Modified nephrectomy group, n = 5; Adenine, 0.25% Adenine diet group, n = 5. One-way ANOVA test followed by Tukey's multiple comparisons test was used. * $P < 0.05$, ** $P < 0.01$.

Disease Models & Mechanisms • Supplementary information

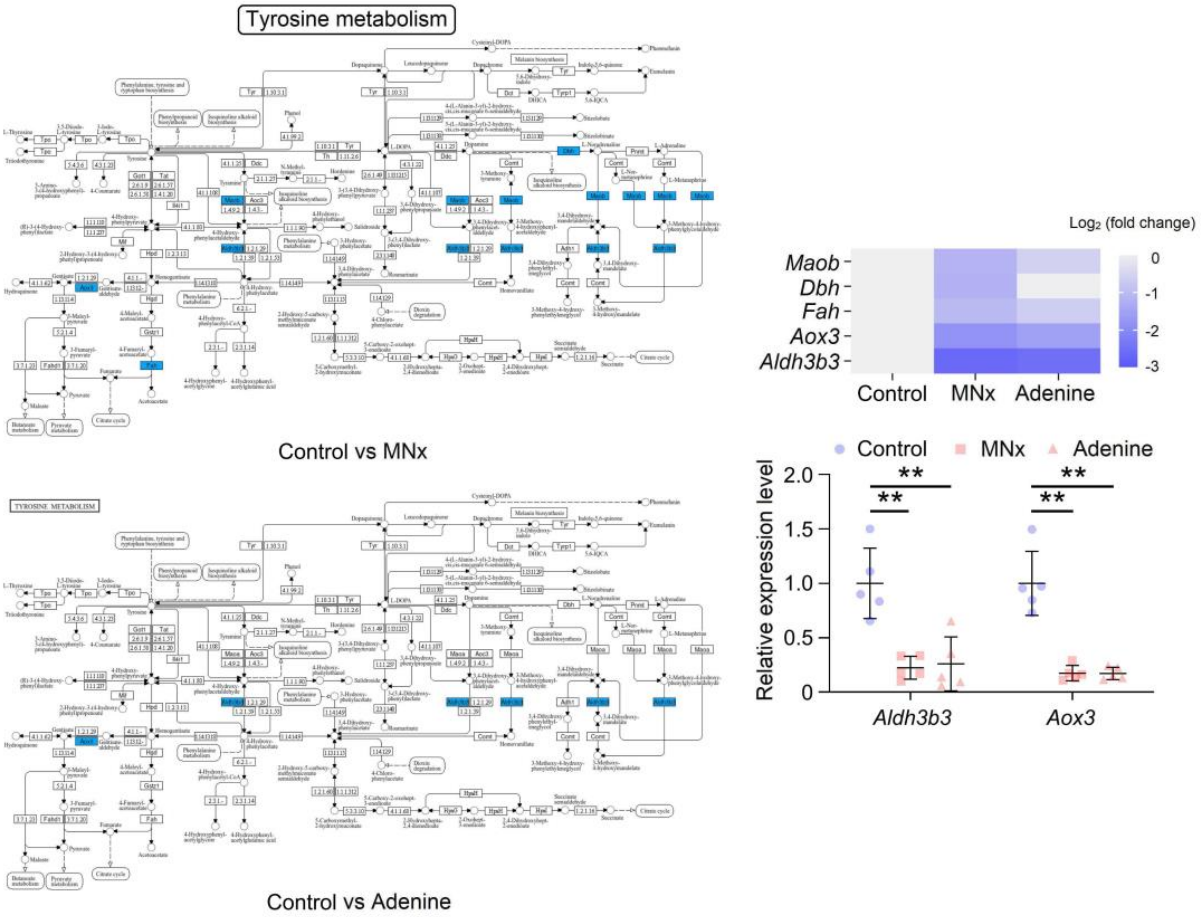

Fig. S21. Alternations of AA metabolism in UC. Both MNx- and adenine-induced UC demonstrated alternations of tyrosine metabolism, while adenine-induced UC had minor changes compared to MNx. qPCR analysis showed similar results compared to RNA sequencing. Control, Control group, n = 5; MNx, Modified nephrectomy group, n = 5; Adenine, 0.25% Adenine diet group, n = 5. One-way ANOVA test followed by Tukey's multiple comparisons test was used. ** $P < 0.01$.

Disease Models & Mechanisms • Supplementary information

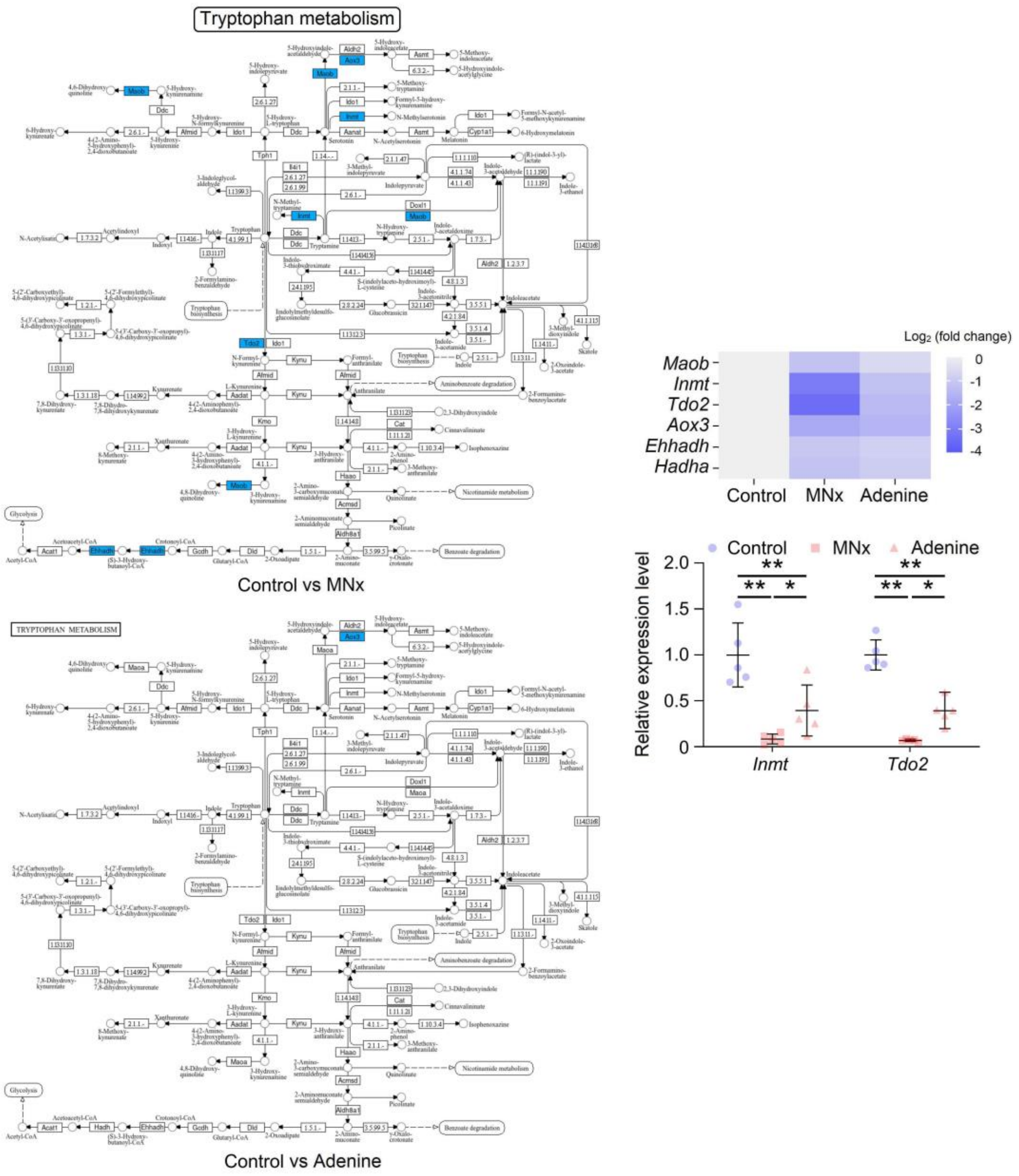

Fig. S22. Alternations of AA metabolism in UC. Both MNx- and adenine-induced UC demonstrated alternations of tryptophan metabolism, while adenine-induced UC had minor changes compared to MNx. qPCR analysis demonstrated less change of related metabolic enzymes in adenine-induced UC as well. Control, Control group, n = 5; MNx, Modified nephrectomy group, n = 5; Adenine, 0.25% Adenine diet group, n = 5. One-way ANOVA test followed by Tukey's multiple comparisons test was used. * P < 0.05, ** P < 0.01.

Disease Models & Mechanisms • Supplementary information

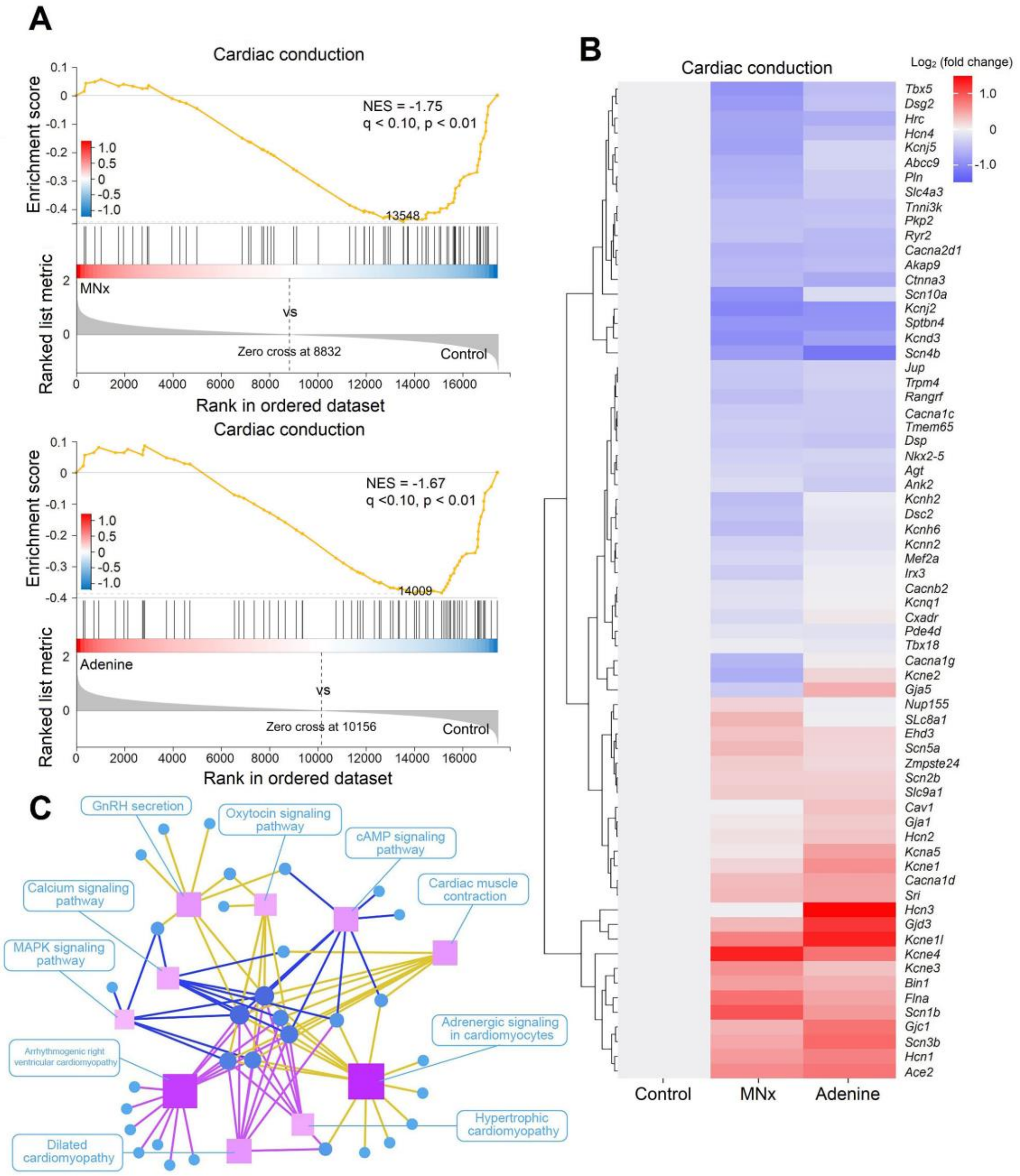

Fig. S23. Analysis on ion channels in UC. (A) GSEA showed downregulated process of cardiac conduction in both MNx- and adenine-induced UC. (B) Heatmap of cardiac conduction-related genes. (C) KEGG network demonstrated that several signalings are associated with cardiac conduction-related genes. Control, Control group, n =5; MNx, Modified nephrectomy group, n =5; Adenine, 0.25% Adenine diet group, n =5.

Disease Models & Mechanisms • Supplementary information

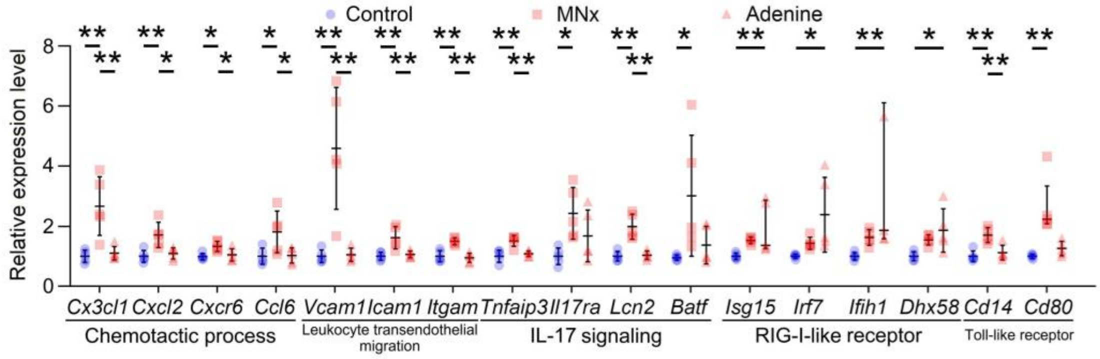

Fig. S24. qPCR confirmation of genes related to inflammatory response in MNx- and adenine-induced UC. Control, Control group, n = 5; MNx, Modified nephrectomy group, n = 5; Adenine, 0.25% Adenine diet group, n = 5. One-way ANOVA test followed by Tukey's multiple comparisons test or Kruskal-Walli's test followed by Dunn's multiple comparisons test was used. * P < 0.05, ** P < 0.01.

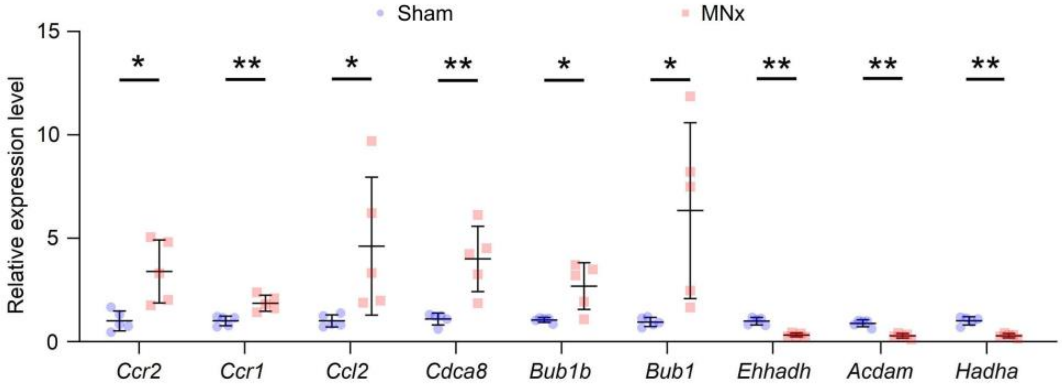

Fig. S25. qPCR confirmation of expression level of identified hub genes in LV tissues from model 6. Sham, Sham group, n = 5; MNx, Modified nephrectomy group, n = 5. One-way ANOVA test followed by Tukey's multiple comparisons test. * P < 0.05, ** P < 0.01.

Disease Models & Mechanisms • Supplementary information

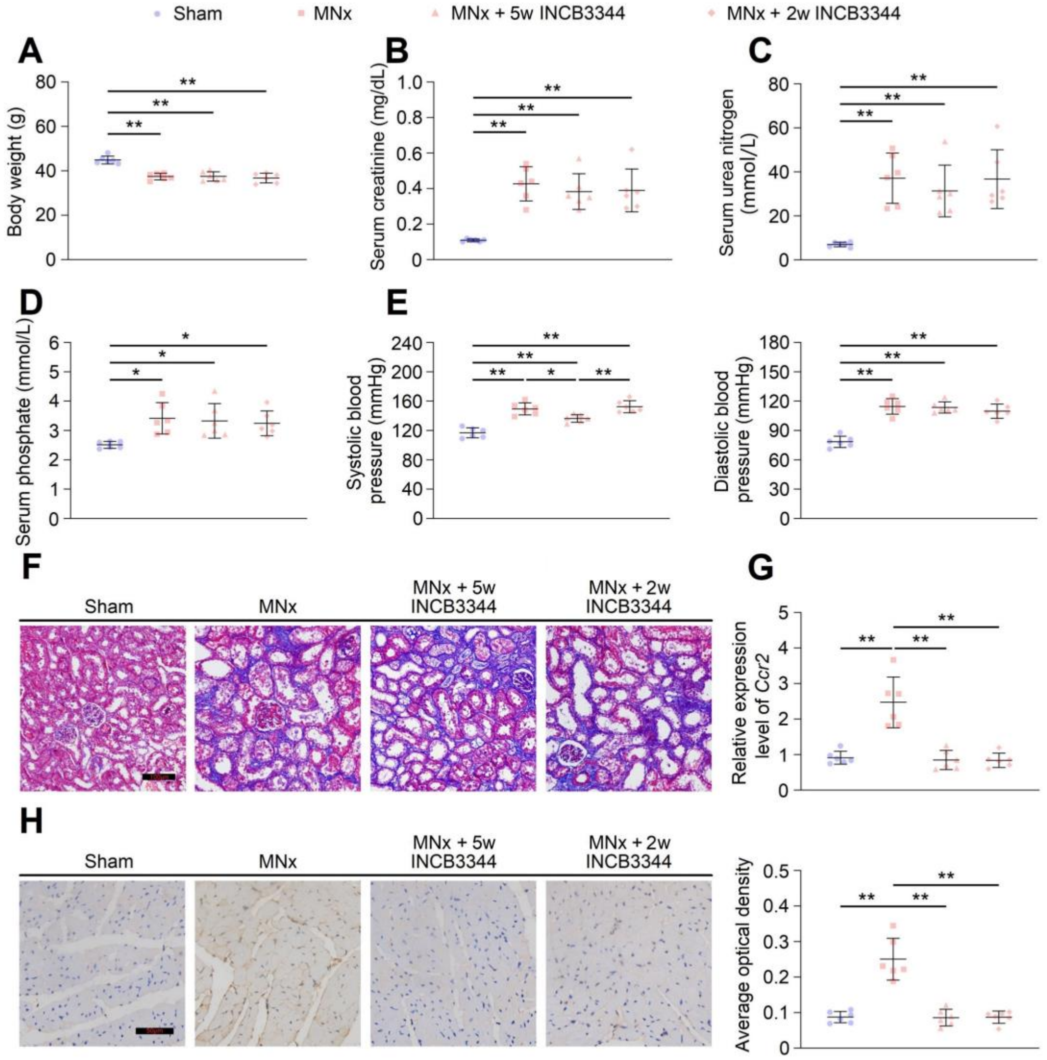

Fig. S26. INCB3344 injection does not affect renal function but suppresses cardiac CCR-2 expression. (A-D) INCB3344 injection did not affect body weight, renal functions, and circulating phosphate. (E) 5-week CCR-2 inhibition slightly reduced systolic pressure in mice with MNx, but 2-week CCR-2 inhibition did not. (F) INCB3344 injection was unable to mitigate renal fibrosis (bar = 100 μm). (G-H) INCB3344 injection decreased CCR-2 expression in LV tissues (bar = 50 μm). Sham, Sham group, n = 6; MNx, Modified nephrectomy group, n = 6; MNx + 5w INCB3344, Modified nephrectomy group with 5-week INCB3344 injection, n = 6; MNx + 2w INCB3344, Modified nephrectomy group with 2-week INCB3344 injection, n = 6. One-way ANOVA test followed by Tukey's multiple comparisons test. * $P < 0.05$, ** $P < 0.01$.

Disease Models & Mechanisms • Supplementary information

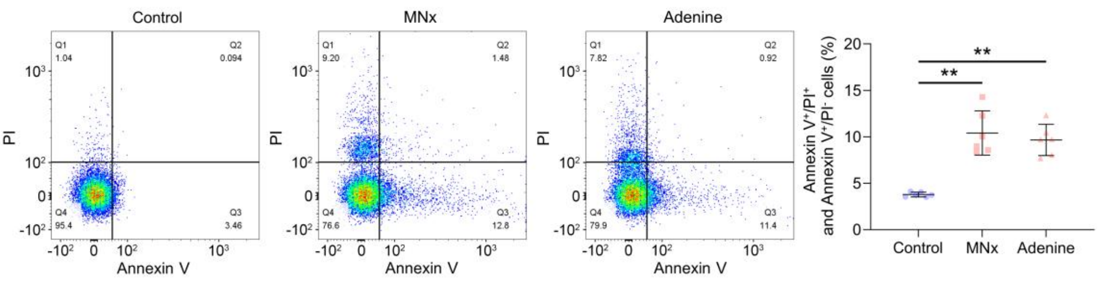

Fig. S27. Flow cytometry analysis on cardiomyocyte apoptosis. Increased apoptotic and necrotic cardiomyocytes can be observed in mice with MNx and adenine. Control, Control group, n = 6; MNx, Modified nephrectomy group, n = 6; Adenine, 0.25% Adenine diet group, n = 6. One-way ANOVA test followed by Tukey's multiple comparisons test was used. ** P < 0.01.

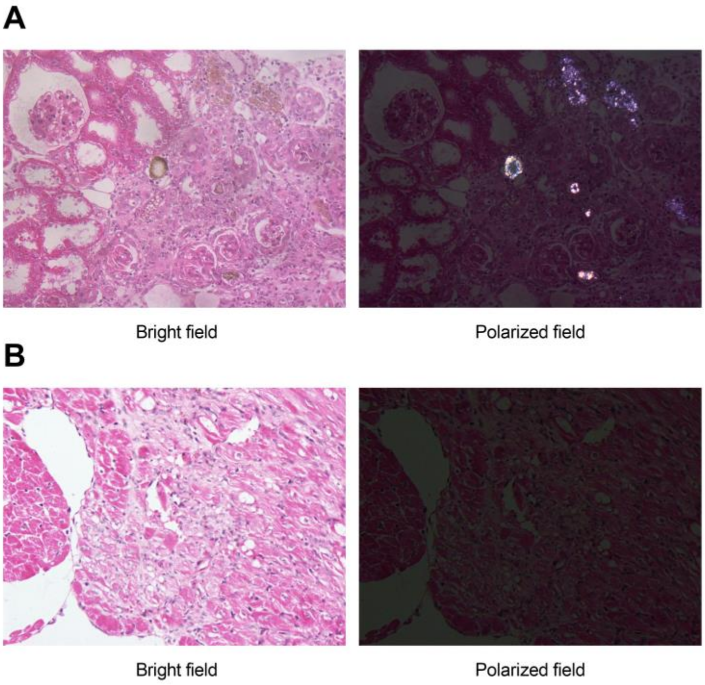

Fig. S28. Polarized microscopy presents DHA deposition in kidney but not in myocardial tissues derived from model 8. (A) Bright and polarized fields of renal tissue. (B) Bright and polarized fields of myocardial tissue.

Disease Models & Mechanisms • Supplementary information

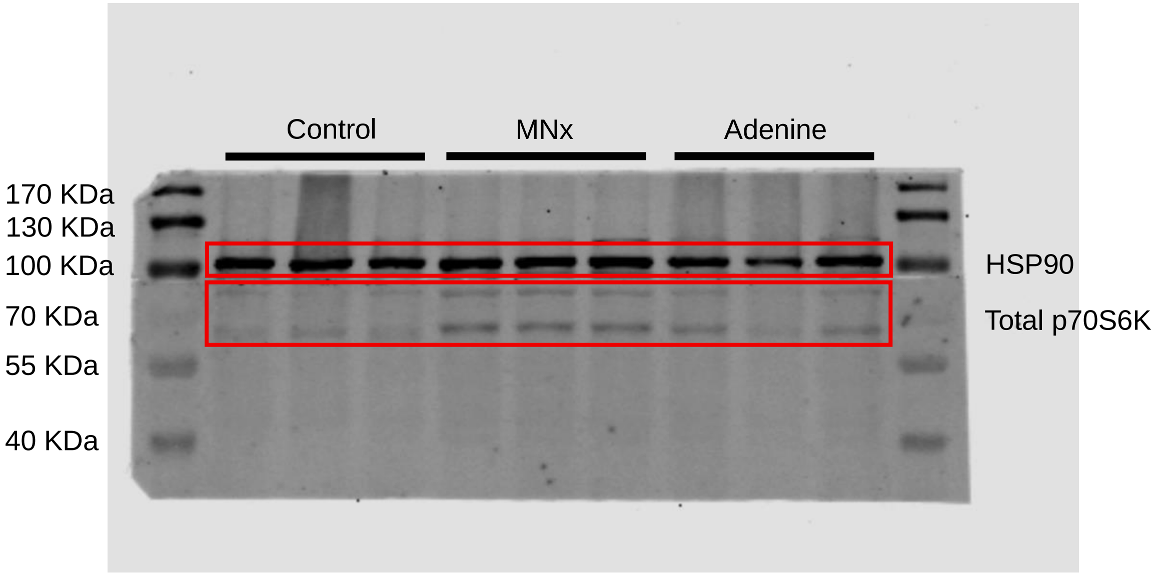

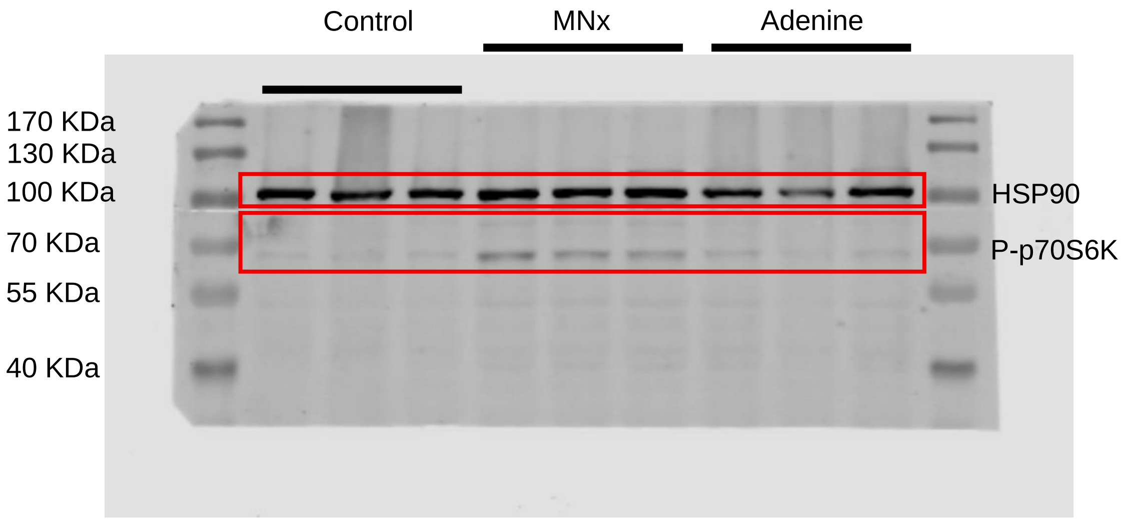

Fig. S29. Uncropped gels.

Disease Models & Mechanisms • Supplementary information

Table S1. Brief information about rodent diet composition

	Protein (g/kg)	Fat (g/kg)	Crude fiber (g/kg)	Calcium (g/kg)	Phosphate (g/kg)	Total calories (kcal/kg)	Adenine (g/kg)
Model 1	180	40	50	14	9	3656	0
Model 2	180	40	50	14	9	3656	0
Model 3	180	40	50	14	9	3656	0
Model 4	180	40	50	14	9	3656	0
Model 5	180	40	50	14	9	3656	0
Model 6	180	40	50	14	9	3656	0
Model 7	180	40	50	14	9	3656	0
Model 8	180	40	50	14	9	3656	2.5
Model 9	180	40	50	14	9	3656	3

Disease Models & Mechanisms • Supplementary information

Table S2. The sequence of primers

Primer	Sequence	Primer	Sequence
Rat *Nppa*	Forward: CGGAAGCTGTTGCAGCCTA<br>Reverse: GCCCTGAGCGAGCAGACCGA	Rat *Nppb*	Forward: CCAGAACAATCCACGATGC<br>Reverse: TCGAAGTCTCTCCTGGATCC
Mouse *Acadm*	Forward: AGGATGACGGAGCAGCCAATGA<br>Reverse: GCCGTTGATAACATACTCGTCAC	Mouse *Acca2*	Forward: TCTGCTGGCAAAGTTCCACCTG<br>Reverse: ACAGAGCCTGTTGAGGGTAAGG
Mouse *Acot2*	Forward: AAGAAGCCGTGAACTACCTGCG<br>Reverse: TGTGATGCCCTTCAGGAAGGAG	Mouse *Adgre1*	Forward: CGTGTTGTTGGTGGCACTGTGA<br>Reverse: CCACATCAGTGTTCCAGGAGAC
Mouse *Aldh3b3*	Forward: GCTACATTGGTCATCAAACAGGT<br>Reverse: CCATCCCACTCGATCCCACT	Mouse *Aox3*	Forward: AAAAAGGTCACCGAGAGGAATG<br>Reverse: GGGGTCGTATCTTGAGATCATCA
Mouse *Batf*	Forward: AGAAGGCTGACAAGCTCCACGA<br>Reverse: CATCTTCTCGTGCTCCTTCAGC	Mouse *Bckdha*	Forward: ATGGCTATGCCATCTCCACACC<br>Reverse: CAAACACATCGTTGCCGTCCAC
Mouse *Bub1*	Forward: TGCCACAGTGTGGACCAGAAAC<br>Reverse: GACAGTTGGTGATGGCTGCACT	Mouse *Bub1b*	Forward: GTCCACAGGTTCTCAATGCCCA<br>Reverse: TGATGGCGTCTTCACTCAGAGG
Mouse *Cbs*	Forward: GGAAAATTGGGAACACCCCTAT<br>Reverse: CCACCCGCATTGAAGAACTCA	Mouse *Ccl2*	Forward: GCTACAAGAGGATCACCAGCAG<br>Reverse: GTCTGGACCCATTCCTTCTTGG
Mouse *Ccl6*	Forward: CACCAGTGGTGGGTGCATCAAG<br>Reverse: GTGCTTAGGCACCTCTGAACTC	Mouse *Ccl12*	Forward: GCTACAGGAGAATCACAAGCAGC<br>Reverse: ACGTCTTATCCAAGTGGTTTATGG
Mouse *Ccr1*	Forward: GCCAAAAGACTGCTGTAAGAGCC<br>Reverse: GCTTTGAAGCCTCCTATGCTGC	Mouse *Ccr2*	Forward: GCTGTGTTTGCCTCTCTACCAG<br>Reverse: CAAGTAGAGGCAGGATCAGGCT
Mouse *Ccn2*	Forward: TGCGAAGCTGACCTGGAGGAAA<br>Reverse: CCGCAGAACTTAGCCCTGTATG	Mouse *Cd14*	Forward: TTGAACCTCCGCAACGTGTCGT<br>Reverse: CGCAGGAAAAGTTGAGCGAGTG
Mouse *Cd68*	Forward: GGCGGTGGAATACAATGTGTCC<br>Reverse: AGCAGGTCAAGGTGAACAGCTG	Mouse *Cd80*	Forward: CCTCAAGTTTCCATGTCCAAGGC<br>Reverse: GAGGAGAGTTGTAACGGCAAGG
Mouse *Cd163*	Forward: GGCTAGACGAAGTCATCTGCAC<br>Reverse: CTTCGTTGGTCAGCCTCAGAGA	Mouse *Cdc6*	Forward: CGCAAAGTGTCTGCTGTTTCAGG<br>Reverse: GGAGAGTGGTTTGAGGACTGTC
Mouse *Cdca8*	Forward: CCAGTCCATACAAGGAAGAGGC<br>Reverse: TGCGTAGCCCTGGAGTCTTGAA	Mouse *Chrna2*	Forward: CCTCTTTGGAGGCTACAATCGC<br>Reverse: CTGCTTTAGCCAGACATTGGTGG
Mouse *Col1a1*	Forward: CCTCAGGGTATTGCTGGACAAC<br>Reverse: CAGAAGGACCTTGTTTGCCAGG	Mouse *Col8a1*	Forward: GGAAATCCCACCTGTGCCAAGA<br>Reverse: TCCTCTTGGTCCAGGTTCTCCA
Mouse *Col9a2*	Forward: AAGGGGCCTCCAGGTAAAGTT<br>Reverse: TCCCATTAAACCATCAATGCCA	Mouse *Cpt2*	Forward: GATGGCTGAGTGCTCCAAATACC<br>Reverse: GCTGCCAGATACCGTAGAGCAA
Mouse *Cx3cl1*	Forward: CAGTGGCTTTGCTCATCCGCTA<br>Reverse: AGCCTGGTGATCCAGATGCTTC	Mouse *Cxcl2*	Forward: CATCCAGAGCTTGAGTGTGACG<br>Reverse: GGCTTCAGGGTCAAGGCAAACT
Mouse *Cxcr6*	Forward: GGTTCTTCCTGCCATTGCTCAC<br>Reverse: GCAGGAACACAGCCACTACAAG	Mouse *Dbt*	Forward: TCAATGCCTCCGTGGACGAGAA<br>Reverse: GGCGATCTCAAATACAGAGCGG
Mouse *Dhx58*	Forward: ATCGCTCAGTGCAATGTGGTGG<br>Reverse: CTCTGTAGCCAGGAAGGAGTAC	Mouse *Eci1*	Forward: CAGAGTTCACCATCAGCCTGGA<br>Reverse: GCCATACATCTCCAGCAAGTCC
Mouse *Ehhadh*	Forward: CAACTCCCTCAGGAGCATCTTG<br>Reverse: GGTCTGACTCTACAGCAACCAC	Mouse *Fh1*	Forward: GAACTCACACGCAGGATGCTGT<br>Reverse: GGCGGCTTTTATTCTCACCATCG
Mouse *Fn1*	Forward: CCCTATCTCTGATACCGTTGTCC<br>Reverse: TGCCGCAACTACTGTGATTCGG	Mouse *Gatm*	Forward: TGGTGCCAAGTGGACAACAGCA<br>Reverse: GCAAGGCTCAAACTCAGTCGTC
Mouse *Gcat*	Forward: TAGTCGGCTGTGCTTCTAAGGC	Mouse *Gnmt*	Forward: TGGTGATCGACCACCGCAACTA

Disease Models & Mechanisms • Supplementary information

	Reverse: GCTCCTGAGACAGTAAAACCAGC		Reverse: GTCGTAATGTCCTTGGTCAGGTC
Mouse *Hadha*	Forward: GTTTGAGGACCTCGGTGTAAAGC<br>Reverse: GAGAGCAGATGTGTTGCTGGCA	Mouse *Icam1*	Forward: AAACCAGACCCTGGAACTGCAC<br>Reverse: GCCTGGCATTTCAGAGTCTGCT
Mouse *Idh2*	Forward: GGCTGTCAAGTGTGCCACAATC<br>Reverse: TTGGCTCTCTGAAGACGGTTCC	Mouse *Ifih1*	Forward: TGCGGAAGTTGGAGTCAAAGCG<br>Reverse: CACCGTCGTAGCGATAAGCAGA
Mouse *Il1b*	Forward: TGGACCTTCCAGGATGAGGACA<br>Reverse: GTTCATCTCGGAGCCTGTAGTG	Mouse *Il17ra*	Forward: CTGTATGACCTGGAGGCTTTCTG<br>Reverse: CGAGTAGACGATCCAGACCTTC
Mouse *Il6*	Forward: TACCACTTCACAAGTCGGAGGC<br>Reverse: CTGCAAGTGCATCATCGTTGTTC	Mouse *Incep*	Forward: GAAGGTAGAGGAAGACAAGCGG<br>Reverse: CCTCCTTCATCTGTTCCACCCT
Mouse *Inmt*	Forward: AGCCTACGACTGGTCCTCCATA<br>Reverse: CATCACACCTCAGGACACGAGT	Mouse *Irf7*	Forward: CCTCTGCTTTCTAGTGATGCCG<br>Reverse: CGTAAACACGGTCTTGCTCCTG
Mouse *Isg15*	Forward: CATCCTGGTGAGGAACGAAAGG<br>Reverse: CTCAGCCAGAACTGGTCTTCGT	Mouse *Itgam*	Forward: TACTTCGGGCAGTCTCTGAGTG<br>Reverse: ATGGTTGCCTCCAGTCTCAGCA
Mouse *Ivd*	Forward: TACGGTGCTCACTCCAACCTCT<br>Reverse: AAGGCTCCGATGAACTCACCAC	Mouse *Lcn2*	Forward: GGACCAGGGCTGTCGCTACT<br>Reverse: GGTGGCCACTTGCACATTGT
Mouse *Lgmn*	Forward: ATCAACCGACCTAACGGCACAG<br>Reverse: ACAGCTTCTGCGTCACCTCTCA	Mouse *Lig1*	Forward: ACCAAGGACACCGAGCAGATTG<br>Reverse: CACACCGTCAAGGTAGTCCTTC
Mouse *Lyve1*	Forward: ACCAGGTAGAGTCAGCGCAGAA<br>Reverse: CAGGACACCTTTGCCATTCTTCC	Mouse *Maob*	Forward: TACTTGGGGACCGAGTGAAGCT<br>Reverse: CCAAAGCAGGTGGAATGGCACT
Mouse *Mcm3*	Forward: CTGCTGTCACTACAGACCAGGA<br>Reverse: ATCACCTCGTGGATGGCTGTGC	Mouse *Mcm4*	Forward: TTCAGCCTTGGCTCCCAGCATT<br>Reverse: GAAGGATGTTGATCTCAGCACGG
Mouse *Mdh2*	Forward: TCACTCCTGCTGAAGAACAGCC<br>Reverse: CCTTTGAGGCAATCTGGCAACTG	Mouse *Mmp2*	Forward: CAAGGATGGACTCCTGGCACAT<br>Reverse: TACTCGCCATCAGCGTTCCCAT
Mouse *Mrc1*	Forward: GTTCACCTGGAGTGATGGTTCTC<br>Reverse: AGGACATGCCAGGGTCACCTTT	Mouse *Nppa*	Forward: CTCCAATCCTGTCAATCCTACC<br>Reverse: TCCGATAGATCTGCCCTCTT
Mouse *Nppb*	Forward: AGTCCTTCGGTCTCAAGGCA<br>Reverse: CCGATCCGGTCTATCTTGTGC	Mouse *Pdk4*	Forward: GTCGAGCATCAAGAAAACCGTCC<br>Reverse: GCGGTCAGTAATCCTCAGAGGA
Mouse *Pola1*	Forward: CTCCTCCACTTGTGGTGATGTC<br>Reverse: GAGGCTCTGGAGGTGCTTTATC	Mouse *Shmt1*	Forward: CTGGAGATGCTGTGTCAGAAGC<br>Reverse: TGAGGCTCTACCAGGGCAGTAT
Mouse *Smc2*	Forward: GATGAGCTGAGGACCAAGGAGA<br>Reverse: CCTCTGTCTTCATCTCCCACTG	Mouse *Tdo2*	Forward: GGCATGGCTGGAAAGAACAC<br>Reverse: CTCCCTGGAGTGCACGGTAT
Mouse *Tgfb1*	Forward: TGATACGCCTGAGTGGCTGTCT<br>Reverse: CACAAGAGCAGTGAGCGCTGAA	Mouse *Timd4*	Forward: AGCTTCTCCGTACAGATGGAA<br>Reverse: CCCACTGTCACCTCGATTGG
Mouse *Tnf*	Forward: GGTGCCTATGTCTCAGCCTCTT<br>Reverse: GCCATAGAACTGATGAGAGGGAG	Mouse *Tnfaip3*	Forward: ACCATGCACCGATACACGC<br>Reverse: AGCCACGAGCTTCCTGACT
Mouse *Vcam1*	Forward: GCTATGAGGATGGAAGACTCTGG<br>Reverse: ACTTGTGCAGCCACCTGAGATC	Mouse *Wt1*	Forward: GGTTTTCTCGCTCAGACCAGCT<br>Reverse: ATGAGTCCTGGTGTGGGTCTTC
Rn18s	Forward: TTTCTCGATTCCGTGGGTGG<br>Reverse: AGCATGCCAGAGTCTCGTTC		

Disease Models & Mechanisms • Supplementary information

Table S3. Echocardiographic results of SD rats in the 5th week after MNx

	Male		Female	
	**Sham (n = 6)**	**MNx (n = 6)**	**Sham (n = 6)**	**MNx (n = 6)**
LVAW; d (mm)	1.80 ± 0.14	2.39 ± 0.08**	1.66 ± 0.07	2.10 ± 0.26**
LVAW; s (mm)	3.07 ± 0.47	3.79 ± 0.36*	3.06 ± 0.32	3.27 ± 0.43
LVPW; d (mm)	1.87 ± 0.20	2.70 ± 0.13**	1.84 ± 0.20	2.18 ± 0.28*
LVPW; s (mm)	2.80 ± 0.25	3.59 ± 0.25**	3.26 ± 0.19	3.45 ± 0.35
LVID; d (mm)	7.32 ± 0.38	7.09 ± 0.25	6.52 ± 0.39	7.79 ± 1.33*
LVID; s (mm)	4.30 ± 0.54	3.82 ± 0.59	2.82 ± 0.54	4.61 ± 1.13**
LV vol; d (μL)	282.50 ± 32.65	263.60 ± 21.23	218.40 ± 29.67	335.10 ± 143.30**
LV vol; s (μL)	83.92 ± 24.91	64.68 ± 20.65	31.49 ± 14.81	104.80 ± 65.50**
LVEF (%)	70.75 ± 5.84	75.68 ± 7.23	71.90 ± 3.90	70.03 ± 7.34
LV Fractional shortening (%)	41.96 ± 5.15	46.23 ± 7.40	42.90 ± 3.62	41.28 ± 6.36**
Left ventricular weight (mg)	778.20 ± 56.35	1224.00 ± 38.83**	612.00 ± 34.14	1105.00 ± 369.30**

Note: Two-tailed t test was used. * $P < 0.05$, ** $P < 0.01$. LVAW; d, diastolic LV anterior wall thickness; LVAW; s, systolic LV anterior wall thickness; LVPW; d, diastolic LV posterior wall thickness; LVPW; s, systolic ventricular posterior wall thickness; LVID; d, diastolic LV diameter; LVID; s, Systolic LV diameter; LV vol; s, left ventricle end-systolic volume; LV vol; d, left ventricle end-diastolic volume; LVEF, left ventricular ejection fraction.

Disease Models & Mechanisms • Supplementary information

Table S4. Echocardiographic results of C57BL/6J and BALB/c mice in the 12th week after MNx

	C57BL/6J		BALB/c	
	Sham (n = 6)	MNx (n = 6)	Sham (n = 8)	MNx (n = 8)
LVAW; d (mm)	0.93 ± 0.06	0.91 ± 0.05	0.87 ± 0.08	0.94 ± 0.07
LVAW; s (mm)	1.46 ± 0.08	1.42 ± 0.15	1.52 ± 0.15	1.48 ± 0.11
LVPW; d (mm)	0.84 ± 0.11	0.88 ± 0.07	0.84 ± 0.07	0.95 ± 0.23
LVPW; s (mm)	1.35 ± 0.13	1.34 ± 0.17	1.33 ± 0.15	1.35 ± 0.26
LVID; d (mm)	4.08 ± 0.16	4.09 ± 0.29	4.02 ± 0.25	3.77 ± 0.35
LVID; s (mm)	2.61 ± 0.17	2.63 ± 0.30	2.48 ± 0.22	2.43 ± 0.52
LV vol; d (μL)	73.52 ± 6.73	74.04 ± 12.27	71.38 ± 10.76	61.43 ± 13.43
LV vol; s (μL)	25.06 ± 3.93	25.89 ± 7.42	22.12 ± 5.19	22.19 ± 11.08
LVEF (%)	65.92 ± 4.20	65.55 ± 4.21	69.01 ± 5.16	65.87 ± 10.61
LV Fractional shortening (%)	35.96 ± 3.24	35.65 ± 2.96	38.39 ± 4.17	36.20 ± 7.95
Left ventricular weight (mg)	112.00 ± 6.39	113.90 ± 16.52	104.10 ± 10.69	107.90 ± 20.91

Note: Two-tailed t test was used. LVAW; d, diastolic LV anterior wall thickness; LVAW; s, systolic LV anterior wall thickness; LVPW; d, diastolic LV posterior wall thickness; LVPW; s, systolic ventricular posterior wall thickness; LVID; d, diastolic LV diameter; LVID; s, Systolic LV diameter; LV vol; s, left ventricle end-systolic volume; LV vol; d, left ventricle end-diastolic volume; LVEF, left ventricular ejection fraction.

Disease Models & Mechanisms • Supplementary information

Table S5. Echocardiographic results of CD-1 mice in the 5th week after MNx

	Male		Female	
	**Sham (n = 6)**	**MNx (n = 6)**	**Sham (n = 6)**	**MNx (n = 6)**
LVAW; d (mm)	0.85 ± 0.03	1.09 ± 0.11**	0.76 ± 0.09	1.08 ± 0.08**
LVAW; s (mm)	1.37 ± 0.06	1.59 ± 0.15**	1.34 ± 0.24	1.74 ± 0.18**
LVPW; d (mm)	0.89 ± 0.09	1.05 ± 0.10*	0.81 ± 0.06	1.03 ± 0.12**
LVPW; s (mm)	1.27 ± 0.08	1.47 ± 0.11**	1.37 ± 0.12	1.55 ± 0.18
LVID; d (mm)	4.16 ± 0.28	4.09 ± 0.25	4.07 ± 0.24	4.08 ± 0.37
LVID; s (mm)	2.71 ± 0.34	2.69 ± 0.34	2.52 ± 0.31	2.43 ± 0.35
LV vol; d (μL)	77.01 ± 11.89	74.06 ± 10.07	73.06 ± 9.86	73.92 ± 15.83
LV vol; s (μL)	27.90 ± 9.05	27.23 ± 7.82	23.26 ± 6.66	21.35 ± 7.48
LVEF (%)	64.36 ± 6.26	63.79 ± 7.24	68.65 ± 5.97	71.49 ± 6.72
LV Fractional shortening (%)	35.04 ± 4.39	30.20 ± 5.12	38.19 ± 4.84	40.58 ± 5.36
Left ventricular weight (mg)	111.90 ± 8.37	146.60 ± 15.45**	94.69 ± 15.38	142.10 ± 19.46**

Note: Two-tailed t test was used. * $P < 0.05$, ** $P < 0.01$. LVAW; d, diastolic LV anterior wall thickness; LVAW; s, systolic LV anterior wall thickness; LVPW; d, diastolic LV posterior wall thickness; LVPW; s, systolic ventricular posterior wall thickness; LVID; d, diastolic LV diameter; LVID; s, Systolic LV diameter; LV vol; s, left ventricle end-systolic volume; LV vol; d, left ventricle end-diastolic volume; LVEF, left ventricular ejection fraction.

Disease Models & Mechanisms • Supplementary information

Table S6. Echocardiographic results of CD-1 mice after 5-week 0.25% adenine diet

	Normal (n = 6)	Adenine (n = 6)
LVAW; d (mm)	0.80 ± 0.02	0.76 ± 0.05
LVAW; s (mm)	1.41 ± 0.09	1.19 ± 0.08**
LVPW; d (mm)	0.83 ± 0.09	0.77 ± 0.09
LVPW; s (mm)	1.25 ± 0.13	1.12 ± 0.08
LVID; d (mm)	4.47 ± 0.12	3.84 ± 0.21**
LVID; s (mm)	2.87 ± 0.14	2.53 ± 0.19**
LV vol; d (μL)	91.19 ± 5.62	63.91 ± 8.03**
LV vol; s (μL)	31.36 ± 3.66	23.59 ± 4.31**
LVEF (%)	65.57 ± 3.80	63.29 ± 3.24
LV Fractional shortening (%)	31.36 ± 3.66	23.59 ± 4.31
Left ventricular weight (mg)	116.10 ± 6.75	83.52 ±10.29**

Note: Two-tailed t test was used. ** $P < 0.01$. LVAW; d, diastolic LV anterior wall thickness; LVAW; s, systolic LV anterior wall thickness; LVPW; d, diastolic LV posterior wall thickness; LVPW; s, systolic ventricular posterior wall thickness; LVID; d, diastolic LV diameter; LVID; s, Systolic LV diameter; LV vol; s, left ventricle end-systolic volume; LV vol; d, left ventricle end-diastolic volume; LVEF, left ventricular ejection fraction.

Disease Models & Mechanisms • Supplementary information

Table S7. Echocardiographic results of CD-1 mice fed with 5-week 0.25% adenine or 5-week 0.3% adenine diet

	**Male**		**Female**	
	**Normal (n = 6)**	**Adenine (n = 6)**	**Normal (n = 6)**	**Adenine (n = 6)**
LVAW; d (mm)	0.82 ± 0.03	0.96 ± 0.05**	0.79 ± 0.06	0.95 ± 0.07**
LVAW; s (mm)	1.32 ± 0.07	1.54± 0.13**	1.23 ± 0.10	1.38 ± 0.12*
LVPW; d (mm)	0.82 ± 0.04	0.99 ± 0.10**	0.81 ± 0.08	0.98 ± 0.07**
LVPW; s (mm)	1.28 ± 0.12	1.46 ± 0.09*	1.32 ± 0.13	1.40 ± 0.23
LVID; d (mm)	4.25 ± 0.15	4.20 ± 0.14	4.08 ± 0.18	4.11 ± 0.08
LVID; s (mm)	2.79 ± 0.24	2.73 ± 0.07	2.56 ± 0.20	2.67 ± 0.10
LV vol; d (μL)	80.97 ± 6.65	78.64 ± 6.23	73.67 ± 8.32	74.60 ± 3.66
LV vol; s (μL)	27.41 ± 5.87	27.85 ± 1.87	24.46 ± 4.98	26.26 ± 2.56
LVEF (%)	66.34 ± 5.23	64.38 ± 3.90	67.00 ± 3.16	64.87 ± 1.70
LV Fractional shortening (%)	36.44 ± 4.04	34.86 ± 2.97	36.74 ± 2.34	35.10 ± 1.21
Left ventricular weight (mg)	107.70 ± 6.67	132.60 ± 10.13**	96.89 ± 8.96	127.60 ± 8.80**

Note: Two-tailed t test was used. * $P < 0.05$, ** $P < 0.01$. LVAW; d, diastolic LV anterior wall thickness; LVAW; s, systolic LV anterior wall thickness; LVPW; d, diastolic LV posterior wall thickness; LVPW; s, systolic ventricular posterior wall thickness; LVID; d, diastolic LV diameter; LVID; s, Systolic LV diameter; LV vol; s, left ventricle end-systolic volume; LV vol; d, left ventricle end-diastolic volume; LVEF, left ventricular ejection fraction.

Disease Models & Mechanisms • Supplementary information

Table S8. Echocardiographic results of female CD-1 mice fed with 5-week 0.25% adenine diet and 2- or 4-week normal diet

	7th week		9th week	
	Normal (n = 6)	Adenine (n = 6)	Normal (n = 6)	Adenine (n = 6)
LVAW; d (mm)	0.81 ± 0.06	0.83 ± 0.11	0.80 ± 0.02	0.84 ± 0.07
LVAW; s (mm)	1.31 ± 0.02	1.35 ± 0.12	1.44 ± 0.08	1.41± 0.18
LVPW; d (mm)	0.79 ± 0.07	0.86 ± 0.15	0.81 ± 0.04	0.90 ± 0.13
LVPW; s (mm)	1.28 ± 0.17	1.35 ± 0.08	1.32 ± 0.11	1.37 ± 0.15
LVID; d (mm)	4.11 ± 0.14	4.19 ± 0.11	4.28 ± 0.14	4.22 ± 0.22
LVID; s (mm)	2.65 ± 0.16	2.71 ± 0.15	2.69 ± 0.16	2.74 ± 0.25
LV vol; d (μL)	74.62 ± 5.90	78.16 ± 4.94	82.08 ± 6.13	79.66 ± 9.94
LV vol; s (μL)	25.93 ± 4.06	27.33 ± 3.57	26.81 ± 4.15	28.24 ± 6.66
LVEF (%)	65.26 ± 4.33	64.94 ± 4.91	67.30 ± 4.40	64.28 ± 4.52
LV Fractional shortening (%)	35.48 ± 3.33	35.32 ± 3.72	37.16 ± 3.46	35.22 ± 3.26
Left ventricular weight (mg)	98.35 ± 8.47	110.00 ± 18.35	106.5± 7.94	116.00 ± 15.71

Note: Two-tailed t test was used. LVAW; d, diastolic LV anterior wall thickness; LVAW; s, systolic LV anterior wall thickness; LVPW; d, diastolic LV posterior wall thickness; LVPW; s, systolic ventricular posterior wall thickness; LVID; d, diastolic LV diameter; LVID; s, Systolic LV diameter; LV vol; s, left ventricle end-systolic volume; LV vol; d, left ventricle end-diastolic volume; LVEF, left ventricular ejection fraction.

Disease Models & Mechanisms • Supplementary information

Table S9. Echocardiographic results of male CD-1 mice with MNx and adenine

	MNx		Adenine	
	Sham (n = 6)	MNx (n = 6)	Normal (n = 6)	CKD (n = 6)
LVAW; d (mm)	0.85 ± 0.03	1.09 ± 0.11**	0.82 ± 0.03$^{\&\&}$	0.96 ± 0.05* $^{\&\&\ \#\#}$
LVAW; s (mm)	1.37 ± 0.06	1.59 ± 0.15*	1.32 ± 0.07$^{\&}$	1.54± 0.13$^{\#}$
LVPW; d (mm)	0.89 ± 0.09	1.05 ± 0.10*	0.82 ± 0.04$^{\&\&}$	0.99 ± 0.10$^{\#}$
LVPW; s (mm)	1.27 ± 0.08	1.47 ± 0.11*	1.28 ± 0.12$^{\&}$	1.46 ± 0.09* $^{\#}$
LVID; d (mm)	4.16 ± 0.28	4.09 ± 0.25	4.25 ± 0.15	4.20 ± 0.14
LVID; s (mm)	2.71 ± 0.34	2.69 ± 0.34	2.79 ± 0.24	2.73 ± 0.07
LV vol; d (μL)	77.01 ± 11.89	74.06 ± 10.07	80.97 ± 6.65	78.64 ± 6.23
LV vol; s (μL)	27.90 ± 9.05	27.23 ± 7.82	27.41 ± 5.87	27.85 ± 1.87
LVEF (%)	64.36 ± 6.26	63.79 ± 7.24	66.34 ± 5.23	64.38 ± 3.90
LV Fractional shortening (%)	35.04 ± 4.39	30.20 ± 5.12	36.44 ± 4.04	34.86 ± 2.97
Left ventricular weight (mg)	111.90 ± 8.37	146.60 ± 15.45**	107.70 ± 6.67$^{\&\&}$	132.60 ± 10.13* $^{\#\#}$

Note: One-way ANOVA test followed by Tukey's multiple comparisons test was used. ** compared with Sham, P < 0.01; * compared with Sham, P < 0.05; $^{\&\&}$ compared with MNx, P < 0.01; $^{\&}$ compared with MNx, P < 0.05; $^{\#\#}$ compared with Normal, P < 0.01; $^{\#}$ compared with Normal, P < 0.05. LVAW; d, diastolic LV anterior wall thickness; LVAW; s, systolic LV anterior wall thickness; LVPW; d, diastolic LV posterior wall thickness; LVPW; s, systolic ventricular posterior wall thickness; LVID; d, diastolic LV diameter; LVID; s, Systolic LV diameter; LV vol; s, left ventricle end-systolic volume; LV vol; d, left ventricle end-diastolic volume; LVEF, left ventricular ejection fraction.

Disease Models & Mechanisms • Supplementary information

Table S10. GSEA between model 8 and model 6

GO-biological process name	Enrichment score	Normalized enrichment score	Q value
**Upregulation**			
Response to interferon beta	0.7749242	3.2026856	0
Proton motive force driven ATP synthesis	0.6728073	2.7261138	0
Negative regulation of viral genome replication	0.657897	2.5595875	0
Negative regulation of viral process	0.53575116	2.3856492	0
Regulation of viral genome replication	0.52282	2.2593148	0.001914284
Oxidative phosphorylation	0.48364797	2.1964495	0.003872501
Aerobic respiration	0.4545792	2.1880493	0.003422714
NADH dehydrogenase complex assembly	0.5602644	2.1729743	0.003592555
ATP biosynthetic process	0.4941587	2.1509233	0.004874987
Organic acid catabolic process	0.43255064	2.1397643	0.005280159
Regulation of viral process	0.44052508	2.1366496	0.005114151
Aerobic electron transport chain	0.5379755	2.0665917	0.012231016
Monocarboxylic acid catabolic process	0.44388485	2.0639966	0.011556682
ATP synthesis coupled electron transport	0.5094402	2.0625994	0.010974772
Response to type I interferon	0.49881214	2.0435731	0.013068856
Thioester metabolic process	0.4836198	2.042891	0.01250999
Fatty acid beta oxidation	0.4713823	2.0016644	0.018593209
Cellular respiration	0.4052538	1.9937923	0.01884865
Cellular amino acid catabolic process	0.45225945	1.9687028	0.023259604
Fatty acid catabolic process	0.44691816	1.9684579	0.02216346
Lipid oxidation	0.42247444	1.9679412	0.021207487
Cytoplasmic pattern recognition receptor signaling pathway in response to virus	0.56257373	1.9371395	0.02737903
Response to interferon alpha	0.5614369	1.9222559	0.031112239
Suppression of viral release by host	0.60860425	1.9142402	0.03281928
Retina homeostasis	0.48447442	1.9059513	0.034645967
Xenobiotic catabolic process	0.5595726	1.8809402	0.04269019
Viral genome replication	0.4196248	1.8800842	0.041642908
Regulation of type I interferon mediated signaling pathway	0.49813712	1.8744723	0.04303291
Alpha amino acid catabolic process	0.44795424	1.870298	0.04348368
Interferon gamma mediated signaling pathway	0.59815866	1.8689555	0.042609397
Very long chain fatty acid metabolic process	0.5210685	1.867838	0.041698143
Defense response to symbiont	0.36407933	1.8603575	0.043426096
Tricarboxylic acid cycle	0.53435546	1.859419	0.042381294
Small molecule catabolic process	0.3598055	1.8516041	0.044412088
Negative regulation of type I interferon mediated signaling pathway	0.55851686	1.8449914	0.04577882
Branched chain amino acid catabolic process	0.6014194	1.844485	0.044621754
**Downregulation**			

Disease Models & Mechanisms • Supplementary information

DNA templated DNA replication	-0.5931329	-2.4229403	0
Cell cycle DNA replication	-0.72893894	-2.374874	0
DNA replication initiation	-0.74860805	-2.350111	0
DNA unwinding involved in DNA replication	-0.7896425	-2.2768917	0
Regulation of DNA templated DNA replication	-0.62104666	-2.22152	0
Sister chromatid segregation	-0.51692885	-2.215333	0
Protein localization to chromosome centromeric region	-0.72550654	-2.2122428	0
Chromosome separation	-0.54676545	-2.2096837	0
Regulation of chromosome segregation	-0.5439217	-2.1773806	2.50E-04
DNA replication	-0.49576843	-2.1659117	2.25E-04
Regulation of chromosome separation	-0.54483336	-2.158227	2.05E-04
Integrin mediated signaling pathway	-0.5513107	-2.1532612	2.82E-04
Neutrophil mediated immunity	-0.6480385	-2.1411073	3.47E-04
Protein localization to condensed chromosome	-0.7757211	-2.1262343	4.02E-04
Chromosome segregation	-0.46920264	-2.1000936	6.77E-04
Mitotic sister chromatid separation	-0.5464315	-2.0890586	7.76E-04
Nuclear chromosome segregation	-0.47247055	-2.0804057	0.001128821
DNA templated DNA replication maintenance of fidelity	-0.602215	-2.0736132	0.001128261
Cell cycle checkpoint signaling	-0.48881915	-2.0696914	0.001248115
Attachment of spindle microtubules to kinetochore	-0.62335694	-2.0585966	0.001525425
Mitotic cell cycle checkpoint signaling	-0.5007838	-2.0568128	0.001560473
Regulation of cell cycle g2 m phase transition	-0.52353317	-2.0497823	0.001898641
Spindle checkpoint signaling	-0.59105223	-2.0407827	0.002208804
Metaphase plate congression	-0.5543459	-2.0249467	0.00324734
DNA duplex unwinding	-0.60528153	-2.0229094	0.003253129
Positive regulation of chromosome segregation	-0.6399889	-2.0157776	0.003953249
Cell adhesion mediated by integrin	-0.5318021	-2.0097353	0.004388957
Regulation of mitotic sister chromatid segregation	-0.56784785	-2.001544	0.005198904
Mitotic nuclear division	-0.45372602	-1.9991603	0.005408473
Neutrophil migration	-0.5002245	-1.9966208	0.005529439
Cell cycle G2-M phase transition	-0.4847402	-1.9874729	0.006117822
Positive regulation of cell division	-0.5269751	-1.9864743	0.006103599
Negative regulation of cell cycle G2-M phase transition	-0.5417522	-1.9821054	0.006329262
DNA conformation change	-0.55381227	-1.9759681	0.006540789
Cellular extravasation	-0.52790254	-1.9740942	0.006482269
Myeloid leukocyte mediated immunity	-0.5004173	-1.9717643	0.006552936
Regulation of cell cycle checkpoint	-0.57694346	-1.9697474	0.006559422
T cell activation involved in immune response	-0.5068193	-1.9692558	0.006535729
Positive regulation of cell cycle process	-0.45122752	-1.9607384	0.0073542
DNA recombination	-0.44150254	-1.9573812	0.007651248
Granulocyte migration	-0.47508788	-1.9565499	0.007546638
Myeloid leukocyte migration	-0.45102662	-1.9542583	0.007688611
Mitotic metaphase plate congression	-0.55760366	-1.9434414	0.009216954
Centriole assembly	-0.5739125	-1.9428052	0.009085193

Disease Models & Mechanisms • Supplementary information

Regulation of substrate adhesion dependent cell spreading	-0.5393745	-1.9406955	0.00903348
Leukocyte migration	-0.43141186	-1.9290391	0.01080546
Microtubule organizing center organization	-0.46784383	-1.9254622	0.011105192
Centromere complex assembly	-0.6134348	-1.9247794	0.011039192
Collagen fibril organization	-0.5330887	-1.9226356	0.011159672
Neutrophil chemotaxis	-0.5030289	-1.9198012	0.011298818
Attachment of mitotic spindle microtubules to kinetochore	-0.6845329	-1.9173294	0.01136486
Centrosome duplication	-0.5174459	-1.9159759	0.011405577
Kinetochore organization	-0.65804446	-1.9145088	0.011382202
Substrate adhesion dependent cell spreading	-0.4807321	-1.9093751	0.012196683
Positive regulation of cell cycle	-0.42611817	-1.9078093	0.012344678
DNA replication checkpoint signaling	-0.7158082	-1.9063512	0.012407068
Recombinational repair	-0.457838	-1.8972923	0.013795757
DNA strand elongation	-0.581805	-1.8961055	0.013732628
Regulation of mitotic nuclear division	-0.47586638	-1.8921623	0.014284806
Positive regulation of smooth muscle cell migration	-0.5214721	-1.8906394	0.014574236
Neuroblast proliferation	-0.51321316	-1.8896445	0.014502085
Integrin activation	-0.62280077	-1.8878255	0.014777076
Neutrophil degranulation	-0.7207367	-1.8844097	0.015353324
Response to muramyl dipeptide	-0.6592494	-1.8822083	0.015537218
Regulation of odontogenesis	-0.70347434	-1.8790911	0.016167192
Positive regulation of leukocyte migration	-0.45477122	-1.8784677	0.016145227
Positive regulation of myeloid leukocyte mediated immunity	-0.6259051	-1.8763075	0.016341923
Negative regulation of nuclear division	-0.51947224	-1.87488	0.016384114
Regulation of chromosome organization	-0.43345678	-1.8727999	0.016703824
Heterotypic cell-cell adhesion	-0.5287438	-1.8718283	0.016675182
Positive regulation of cell cycle checkpoint	-0.6889839	-1.867214	0.01755512
Regulation of attachment of spindle microtubules to kinetochore	-0.6713867	-1.8608025	0.019261047
Positive regulation of vascular associated smooth muscle cell migration	-0.65455276	-1.8599012	0.01913803
Mitotic G2-M transition checkpoint	-0.53313994	-1.8507516	0.021645416
Phagocytosis	-0.43139935	-1.8494924	0.02173369
Response to muscle stretch	-0.64511114	-1.848686	0.021729834
Negative regulation of cell cycle process	-0.4177966	-1.8463782	0.022314994
Regulation of mitotic cell cycle phase transition	-0.41379526	-1.8456584	0.022188239
Cell chemotaxis	-0.42021465	-1.8454653	0.021935934
Negative regulation of adaptive immune response	-0.52688146	-1.844533	0.021916613
Granulocyte chemotaxis	-0.46279716	-1.8422296	0.022302404
Motor neuron axon guidance	-0.57840186	-1.8414675	0.022277895
Membrane invagination	-0.4950893	-1.8384628	0.023004
Amyloid beta clearance	-0.5824225	-1.838454	0.022730142
Positive regulation of smooth muscle cell proliferation	-0.4678575	-1.8351415	0.02351507

Positive regulation of extracellular matrix organization	-0.59022266	-1.8347489	0.023333803
Regulation of myeloid leukocyte mediated immunity	-0.50379705	-1.8318642	0.023948735
Negative regulation of myotube differentiation	-0.68584096	-1.8309032	0.023972886
Regulation of mitotic cell cycle	-0.40249413	-1.829951	0.023996107
Neutrophil activation involved in immune response	-0.627884	-1.8259495	0.02517548
Retina layer formation	-0.64327943	-1.8258744	0.024898827
Regulation of leukocyte migration	-0.4225606	-1.825798	0.024628187
Positive regulation of nitric oxide metabolic process	-0.5332512	-1.8257453	0.024363369
Non membrane bounded organelle assembly	-0.40519428	-1.8200662	0.026173808
Cell activation involved in immune response	-0.41549283	-1.8198518	0.026029233
Leukocyte chemotaxis	-0.4254664	-1.8164029	0.027089823
Vascular associated smooth muscle cell migration	-0.5618237	-1.8161088	0.02688053
Negative regulation of mitotic cell cycle phase transition	-0.43518832	-1.8127376	0.027967395
Regulation of neuroblast proliferation	-0.53564227	-1.8110301	0.028346362
Negative regulation of chromosome organization	-0.47192323	-1.8107488	0.028187605
Cell matrix adhesion	-0.4184335	-1.8086702	0.02867022
Positive regulation of mitotic cell cycle	-0.44934818	-1.8078692	0.028589085
Mitotic cell cycle phase transition	-0.40088752	-1.8074968	0.0284548
Regulation of DNA replication	-0.44661954	-1.8073585	0.028235834
Homologous recombination	-0.50531656	-1.806813	0.028182467
Leukocyte degranulation	-0.4730272	-1.8010464	0.030069916
DNA integrity checkpoint signaling	-0.44468668	-1.794643	0.032398883
Positive regulation of DNA replication	-0.51693404	-1.7937094	0.03250732
Regulation of nuclear division	-0.43768692	-1.7935611	0.03226127
Acute phase response	-0.5466439	-1.7894934	0.033307154
Lymphocyte activation involved in immune response	-0.424587	-1.7890314	0.0331601
Leukocyte proliferation	-0.40050152	-1.7882807	0.033167075
Chromosome localization	-0.4793966	-1.7861228	0.033784587
Interleukin 2 production	-0.48544195	-1.7857504	0.033656623
Muscle cell migration	-0.4383653	-1.7831297	0.034661762
Macrophage migration	-0.4932659	-1.7806486	0.035584196
Chondrocyte development	-0.5479392	-1.7795849	0.03574426
Positive regulation of histone acetylation	-0.5370451	-1.775355	0.03747317
Signal transduction in response to DNA damage	-0.4225648	-1.7752695	0.037196364
Chaperone mediated protein folding	-0.49291202	-1.774932	0.037066113
Regulation of nitric oxide metabolic process	-0.48412284	-1.7732263	0.03751765
T cell differentiation	-0.39714783	-1.7729055	0.037330456
Cell substrate adhesion	-0.3954523	-1.7725276	0.037228458
Inhibitory postsynaptic potential	-0.62530226	-1.7717477	0.037321523
Hematopoietic stem cell proliferation	-0.5521206	-1.7716285	0.03708618
Positive regulation of cell cycle G2-M phase transition	-0.56159943	-1.7713594	0.036943834
Positive regulation of leukocyte cell-cell adhesion	-0.40501386	-1.770507	0.037071995
Positive regulation of leukocyte proliferation	-0.42501494	-1.7697465	0.03712764
Regulation of spindle checkpoint	-0.6146825	-1.7621962	0.040354583

Disease Models & Mechanisms • Supplementary information

Negative regulation of extrinsic apoptotic signaling pathway via death domain receptors	-0.57151747	-1.7620467	0.04013091
Pyrimidine ribonucleoside triphosphate metabolic process	-0.67151254	-1.7612243	0.040239304
Kinetochore assembly	-0.63985556	-1.758878	0.04123473
Female meiotic nuclear division	-0.53970844	-1.7579802	0.04132518
Leukocyte homeostasis	-0.43622795	-1.7575876	0.04126987
Regulation of mammary gland epithelial cell proliferation	-0.6304662	-1.7570391	0.041198846
Lipopolysaccharide mediated signaling pathway	-0.49346814	-1.757007	0.040904015
Myeloid cell activation involved in immune response	-0.4514043	-1.7561088	0.040934112
Negative regulation of cell division	-0.66631263	-1.7548332	0.04139923
Leukocyte mediated immunity	-0.3891259	-1.7538023	0.041597556
Positive regulation of mitotic cell cycle phase transition	-0.45457262	-1.7523894	0.042067226
Cartilage development	-0.4126796	-1.7523214	0.041808985
Histone exchange	-0.66694057	-1.751884	0.04171298
Negative regulation of cell cycle	-0.38828266	-1.7516804	0.041524548
Regulation of calcineurin mediated signaling	-0.52519435	-1.7498851	0.04207627
Pigment cell differentiation	-0.52840626	-1.7483245	0.04265202
Cortical cytoskeleton organization	-0.48419112	-1.7470275	0.043134242
Positive regulation of cell activation	-0.3881514	-1.7468861	0.042902425
Apoptotic process involved in development	-0.52667123	-1.7455765	0.043216106
Regulation of calcium mediated signaling	-0.46038103	-1.7452548	0.04310879
Double strand break repair	-0.39578667	-1.7436823	0.043589167
Positive regulation of cell-cell adhesion	-0.39203426	-1.7428464	0.043781072
Negative regulation of mitotic cell cycle	-0.40616626	-1.7426243	0.04358229
Regulation of cell cycle phase transition	-0.38205495	-1.742336	0.043467525
Regulation of b cell proliferation	-0.47282946	-1.7401515	0.044352792
Leukocyte cell-cell adhesion	-0.38805643	-1.7397243	0.044242084
Spindle organization	-0.41011253	-1.7383004	0.044698566
Microtubule cytoskeleton organization involved in mitosis	-0.4194069	-1.7365363	0.045313675
Substrate dependent cell migration	-0.55159163	-1.7364078	0.04508401
Spindle assembly	-0.4267692	-1.7346963	0.045710698
Leukocyte aggregation	-0.64664936	-1.73429	0.04565111
Developmental growth involved in morphogenesis	-0.3990351	-1.7336766	0.045676462
Leukocyte adhesion to vascular endothelial cell	-0.49498	-1.7324226	0.046064645
Mononuclear cell migration	-0.4095539	-1.7321018	0.045955893
Positive regulation of cell adhesion	-0.38147888	-1.731989	0.045730535
Base excision repair	-0.5251053	-1.7308129	0.046050172
Response to X ray	-0.5714779	-1.7291771	0.046760738
Regulation of muscle organ development	-0.58413815	-1.7277231	0.0473273
Mast cell activation	-0.47204134	-1.7257329	0.048163526
Inositol phosphate mediated signaling	-0.4998531	-1.7236885	0.04910978
Positive regulation of epithelial to mesenchymal transition	-0.5089169	-1.7219267	0.049870543

Disease Models & Mechanisms • Supplementary information

Table S11. GSEA based on Wiki pathway between control group and MNx group

Wiki pathway	Enrichment score	Normalized enrichment score	Q value
**Upregulation**			
TYROBP causal network in microglia	0.70188695	2.3147635	0
Microglia pathogen phagocytosis pathway	0.7331937	2.2541819	0
Prostaglandin synthesis and regulation	0.7223923	2.094081	0
Inflammatory response pathway	0.7293762	2.0866597	0
Fibrin complement receptor 3 signaling pathway	0.6656351	2.0655274	0
Focal adhesion	0.5619781	2.045751	0
Chemokine signaling pathway	0.55404145	2.023693	0
Il5 signaling pathway	0.6019133	1.9812074	0
Regulation of actin cytoskeleton	0.52965343	1.9124278	3.17E-04
Il3 signaling pathway	0.537602	1.8575797	0.001063987
Kit receptor signaling pathway	0.5591605	1.8536079	9.67E-04
Spinal cord injury	0.53699917	1.8450394	0.001048858
Complement activation classical pathway	0.7240221	1.8401521	0.001188532
Apoptosis	0.54511184	1.827188	0.001239759
Integrin mediated cell adhesion	0.52632517	1.8205199	0.00121869
TGF beta signaling pathway	0.567387	1.8110783	0.001436278
P53 signaling	0.5449415	1.8073533	0.001749883
Endochondral ossification	0.5675687	1.802062	0.001758938
G1 to S cell cycle control	0.55922097	1.7889347	0.002423801
Oxidative damage response	0.58263105	1.7800009	0.002736238
FAS pathway and stress induction of HSP regulation	0.58293027	1.7433825	0.00465292
G13 signaling pathway	0.5670202	1.7205653	0.005994993
EGFR1 signaling pathway	0.46445554	1.6988189	0.007676573
Matrix metalloproteinases	0.6042108	1.6906317	0.008279448
Type II interferon signaling	0.5854792	1.6815817	0.008977084
Toll-like receptor signaling	0.5664752	1.6698608	0.010277291
Primary focal segmental glomerulosclerosis	0.5022107	1.665975	0.010113255
EBY lmp1 signaling	0.618276	1.659718	0.010717753
MAPK signaling pathway	0.46053493	1.6596916	0.010348176
Burn wound healing	0.62326264	1.6582654	0.010130785
Alpha 6 beta 4 integrin signaling pathway	0.4986499	1.6468354	0.011133868
Hedgehog signaling pathway	0.60733914	1.6441351	0.011257492
Focal adhesion PI3K AKT mTOR signaling pathway	0.4426647	1.6392852	0.011580024
id signaling pathway	0.50175345	1.5825291	0.02295781
GPCRS small ligand	0.6171197	1.572642	0.025208069
Il1 signaling pathway	0.52517545	1.5578123	0.029123798
Il2 signaling pathway	0.46197453	1.5467159	0.032488693
Cytoplasmic ribosomal proteins	0.45689678	1.5449955	0.03223375
Cytokines and inflammatory response	0.56798744	1.5447807	0.031530134

Disease Models & Mechanisms • Supplementary information

Novel jundmp1 pathway	0.5495787	1.5147622	0.04209569
Mechanisms associated with pluripotency	0.40291858	1.5093431	0.04359084
Notch signaling pathway	0.485748	1.5026171	0.045496877
WNT signaling pathway and pluripotency	0.43776438	1.4925224	0.049121913
Delta notch signaling pathway	0.44066423	1.4892726	0.049613588
**Downregulation**			
Electron transport chain	-0.6927241	-3.0721989	0
Fatty acid beta-oxidation	-0.7798437	-2.9159129	0
TCA cycle	-0.79384995	-2.9019446	0
Mitochondrial long chain fatty acid beta-oxidation	-0.8374606	-2.602946	0
Oxidative phosphorylation	-0.63321793	-2.5531306	0
Amino acid metabolism	-0.5447556	-2.4564338	0
PPAR signaling pathway	-0.52365583	-2.259436	0
Tryptophan metabolism	-0.5628402	-2.1992285	1.41E-04
Fatty acid biosynthesis	-0.62525517	-2.0753176	3.63E-04
Monoamine GPCRs	-0.47022787	-1.6308498	0.035449397
Triacylglyceride synthesis	-0.4874944	-1.6212425	0.033998407

Disease Models & Mechanisms • Supplementary information

Table S12. Echocardiography of MNx male CD-1 with INCB3344 administration

	Sham (n = 6)	MNx (n = 6)	MNx + 5w INCB3344 (n =6)	MNx + 2w INCB3344 (n =6)
LVAW; d (mm)	0.83 ± 0.03	1.12 ± 0.06**	0.99 ± 0.03** &&	1.11 ± 0.02** ##
LVAW; s (mm)	1.42 ± 0.13	1.61 ± 0.17	1.33 ± 0.19	1.66 ± 0.19#
LVPW; d (mm)	0.82 ± 0.04	1.03 ± 0.08**	0.99 ± 0.08**	1.05 ± 0.08**
LVPW; s (mm)	1.43 ± 0.13	1.55 ± 0.25	1.33 ± 0.13	1.54 ± 0.16
LVID; d (mm)	4.24 ± 0.08	4.09 ± 0.10	4.47 ± 0.17* &&	4.18 ± 0.11##
LVID; s (mm)	2.51 ± 0.14	2.47 ± 0.29	3.30 ± 0.22** &&	2.51 ± 0.33##
LV vol; d (μL)	80.54 ± 3.36	73.91 ± 4.39	91.28 ± 8.18* &&	77.75 ± 4.62##
LV vol; s (μL)	22.60 ± 3.01	22.09 ± 6.28	44.43 ± 7.50** &&	23.03 ± 6.74##
LVEF (%)	71.97 ± 3.31	70.32 ± 7.22	51.52 ± 4.76** &&	70.55 ± 8.03##
LV Fractional shortening (%)	40.91 ± 2.83	39.69 ± 6.08	26.25 ± 2.91** &&	40.09 ± 7.25##
Left ventricular weight (mg)	108.30 ± 6.71	146.70 ±10.32**	150.50 ± 17.23**	154.00 ± 11.71**

Note: One-way ANOVA test followed by Tukey's multiple comparisons test. ** compared with Sham, P < 0.01; * compared with Sham, P < 0.05; && compared with MNx, P < 0.01; & compared with MNx, P < 0.05; ## compared with MNx + 5w INCB3344, P < 0.01; # compared with MNx + 5w INCB3344, P < 0.05. LVAW; d, diastolic LV anterior wall thickness; LVAW; s, systolic LV anterior wall thickness; LVPW; d, diastolic LV posterior wall thickness; LVPW; s, systolic ventricular posterior wall thickness; LVID; d, diastolic LV diameter; LVID; s, Systolic LV diameter; LV vol; s, left ventricle end-systolic volume; LV vol; d, left ventricle end-diastolic volume; LVEF, left ventricular ejection fraction.

Table S13. Gene expression read counts.

Available for download at
https://journals.biologists.com/dmm/article-lookup/doi/10.1242/dmm.052395#supplementary-data

Disease Models & Mechanisms • Supplementary information
